# Decision support for aquatic restoration based on species‐specific responses to disturbance

**DOI:** 10.1002/ece3.9313

**Published:** 2022-10-11

**Authors:** James E. McKenna, Catherine Riseng, Kevin Wehrly

**Affiliations:** ^1^ US Geological Survey, Great Lakes Science Center Tunison Laboratory of Aquatic Science Cortland New York USA; ^2^ School of Natural Resources and Environment University of Michigan Ann Arbor Michigan USA; ^3^ Institute for Fisheries Research Michigan Department of Natural Resources and University of Michigan Ann Arbor Michigan USA

**Keywords:** aquatic habitat, conservation, fish habitat, great lakes fish, management tool, natural resource, restoration

## Abstract

Disturbances to aquatic habitats are not uniformly distributed within the Great Lakes and acute effects can be strongest in nearshore areas where both landscape and within lake effects can have strong influence. Furthermore, different fish species respond to disturbances in different ways. A means to identify and evaluate locations and extent of disturbances that affect fish is needed throughout the Great Lakes. We used partial Canonical Correspondence Analysis to separate “natural” effects on nearshore assemblages from disturbance effects. Species‐specific quadratic models of fish abundance as functions of in‐lake disturbance or watershed‐derived disturbance were developed separately for each of 35 species and lakewide predictions mapped for Lake Erie. Most responses were unimodal and more species decreased in abundance with increasing watershed disturbance than increased. However, eight species increased in abundance with current in‐lake disturbance conditions. Optimum Yellow Perch (*Perca flavescens*) abundance occurred at in‐lake disturbance values less than the gradient mean, but decreased continuously from minimum watershed disturbance to higher values. Bands of optimum in‐lake conditions occurred throughout the eastern and western portions of the Lake Erie nearshore zone; some areas were less disturbed than desirable. However, watershed‐derived disturbance conditions were generally poor for Yellow Perch throughout the lake. In contrast, optimum Smallmouth Bass (*Micropterus dolomieu*) abundance occurred at in‐lake disturbance values greater than the gradient mean and continuously increased with increasing watershed disturbance. Smallmouth Bass responses to disturbance indicated that most of the nearshore zone was less disturbed than is desirable and were most abundant in areas that the Yellow Perch response indicated were highly disturbed. Mapping counts of species response models that agreed on the disturbance level in each spatial unit of the nearshore zone showed a fine‐scale mosaic of areas in which habitat restoration may benefit many or few species. This tool may assist managers in prioritizing conservation and restoration efforts and evaluating environmental conditions that may be improved.

## Graphical abstract

Species‐specific non‐linear responses of abundance to Great Lakes disturbance indices for multiple fish species identifies multiscale distributions of disturbed habitat perceived by each species throughout the lakes. The degree of agreement among species was mapped. The results provide decision support for population and community conservation and restoration efforts.

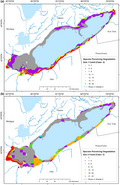

## 1 INTRODUCTION

The Laurentian Great Lakes Region is a vast system of aquatic and terrestrial habitats of widely varying conditions, supporting a diverse array of living communities, including fish which are valuable as harvestable resources, key ecological components, and indicators of environmental conditions. However, fish are threatened by numerous environmental disturbances and influenced by natural conditions (Loftus & Regier, 1972; Ryder, 1972). Disturbances are extensive in the Great Lakes and degrade biodiversity and ecological function (Allan et al., 2017; Christie et al., 1972; Johnson et al., 2016; Uzarski et al., 2017). Resources to manage and rehabilitate fish populations and fish habitats are limited, as is knowledge of where aquatic habitat does and does not support healthy fish populations, and what may be degrading conditions for fish (Kovalenko et al., 2018; Regier & Loftus, 1972). Tools that effectively identify areas where management investment is likely to benefit the most species and ecosystem services could assist with prioritization of resources (Allan et al., 2017).

Extensive environmental disturbances are caused by human activities, which are among the few factors that humans may manipulate to manage fish populations (Smith et al., 2015). These disturbances have numerous sources and are not uniformly distributed in space or time (Allan et al., 2013; Kovalenko et al., 2018; McKenna & Kocovsky, 2020; Wehrly et al., 2013). There has been extensive work to identify environmental stressors that affect fishes in the Great Lakes (e.g. Colby et al., 1972; Johnson et al., 2016; Uzarski et al., 2017). Most of these studies associate metrics and multimetric indices to measures of fish community conditions (e.g., species and guild richness) to make lake‐ or region‐wide estimates of the extent of degraded conditions. Important ecological monitoring programs are based on some of these efforts (e.g., Great Lake Coastal Wetland Monitoring program (GLCWM), Uzarski et al., 2017 and Great Lakes Ecological Indicators (GLEI) program). These programs have focused on either stressors within a watershed or those occurring within the Great Lakes proper, and a few have used both (Kovalenko et al., 2018). Two recently developed multimetric disturbance indices that address each of these realms are the Great Lakes Environmental Assessment Mapping (GLEAM) (Allan et al., 2013) and landscape watershed (Wehrly et al., 2013) indices. The GLEAM index describes a combination of factors mostly from within lake or coastline sources, while the Wehrly watershed index combines watershed‐derived disturbances that are transferred to the Great Lakes through the region's river networks, mostly to the nearshore zone. Together these indices encompass the vast majority of significant disturbance factors affecting the Great Lakes and include both US and Canadian regions.

Coastal wetlands and coastline habitats are important for Great Lakes fishes (Johnson et al., 2016; Kovalenko et al., 2018; Uzarski et al., 2017), but face numerous degrading influences. A recent study used the GLEAM and Wehrly indices, along with data from the GLCWM and GLEI programs, to evaluate anthropogenic disturbances to wetlands and coastal fish habitats (Kovalenko et al., 2018). The study showed degradation of numerous areas, based on reduced species richness and intolerant species occurrences (and other metrics), but also identified high quality areas with few anthropogenic effects. The nearshore zone is also a critical realm for Great Lakes fish life cycles (e.g. Goodyear et al., 1982; Lane et al., 1996; McKenna, 2008, for example). However, the nearshore zone is at the interface between influence from watersheds and within lake processes and little is known about how various anthropogenic factors affect fishes in this realm.

The typical expectation is that the greater the disturbance, the worse conditions are for fish. However, increasing disturbance levels are not consistently detrimental to all fishes. Measures of “tolerant species” are common metrics (Karr, 1981; Riseng et al., 2004) and some studies have noted a relatively high occurrence of intolerant species in areas experiencing high anthropogenic disturbance (Kovalenko et al., 2018). It is logical to expect that different fish species have different preferences for environmental conditions and will therefore exhibit different responses to disturbance in any given location. Differential changes to Great Lakes fishes in response to changing environmental conditions have been observed (Smith, 1972). So‐called tolerant species may actually be enhanced by increasing disturbance, not just tolerant of those conditions. Also, fish are mobile, responding to both natural and disturbed conditions and moving to different areas to satisfy their needs at different life stages (e.g. Atchison et al., 1987; Stabell, 1984). Therefore, we would not expect all fish species to be distributed uniformly in space or time; the greatest abundances are likely to be in the best available habitats. Previously developed multimetric indices of anthropogenic disturbances may not behave as expected when species responding positively to increasing disturbance are included as a measure of degradation for fish communities. Because natural resource managers focus on disturbances and have limited resources, separating the influence of “natural” conditions from anthropogenic disturbances is important. We present a process to make this separation and display the spatial distributions of the effects of human disturbances from the fishes' perspectives, statistically (sensu Magnuson et al., 1980).

In this study, we used the extensive fish and habitat datasets for the Great Lakes Region and build on the statistical approaches of previous researchers to determine the responses of Great Lakes fish abundances to disturbance indices throughout the Great Lakes nearshore zone. Given the assumption that fish can move away from areas of poor condition to accessible areas with better conditions, we can use statistical ordination approaches to control for the effects of “natural” influences and detect the response of fish abundances to multiple stressors. We quantify species‐specific responses of abundance to within‐lake and watershed‐derived disturbances and report on species' preferences along gradients from high to low disturbance conditions. We determine the fish‐disturbance relationships for all of the Great Lakes, but use Lake Erie to illustrate the spatial distribution of fish‐perceived disturbance conditions in the nearshore zone of an entire lake. We use ordination and quadratic regression to develop the relationships between each species' abundance and degree of disturbance, and a geographic information system (GIS) to map the distribution of disturbance and spatial agreement among species, providing a tool that highlights locations with habitat that may be considered for protection or restoration for multiple species.

Our objectives were to (1) separate the “natural” influences from anthropogenic disturbances affecting fish abundances in the Great Lakes, (2) develop quantitative, species‐specific abundance models of response to multimetric indices that include all of the known significant disturbances to fish and fish habitats throughout Great Lakes nearshore zones, (3) use those models and the distribution of disturbances to predict abundance responses for each species at each location (30‐m spatial cell) within the Lake Erie nearshore zone, and (4) quantify species agreement about disturbance conditions in each location by an overlay of those species‐specific maps. The resulting species‐specific disturbance distributions and degrees of species agreement can assist managers with decisions about species on which to focus and in what locations to conduct restoration or protection activities.

## 2 METHODS

### 2.1 Study area

The Great Lakes nearshore zone was defined as water of ~3–30 m depth, except in Lake Erie, where maximum nearshore zone depth was 15 m (Riseng et al., 2018; Wang et al., 2015). “Natural” environmental data (variables resistant to anthropogenic influence) and anthropogenic disturbance data used in this study were attributed to the nearshore zone at the 30‐m spatial cell resolution. The entire nearshore zone of the Laurentian Great Lakes consisted of 53,478,427 spatial cells. These spatial units are grouped within large circulation units called Aquatic Lake Units (ALUs) within each Great Lake (McKenna & Castiglione, 2010). Because the availability of disturbance indices and fish observations was greatest in Lake Erie, we use its nearshore zone to illustrate application of fish prediction models and spatial agreement of fish abundance response to disturbed conditions throughout a great lake. The Lake Erie nearshore zone consisted of 9,770,077 30‐m cells (Forsyth et al., 2016; Riseng et al., 2018).

### 2.2 Data

**FIGURE 1:**
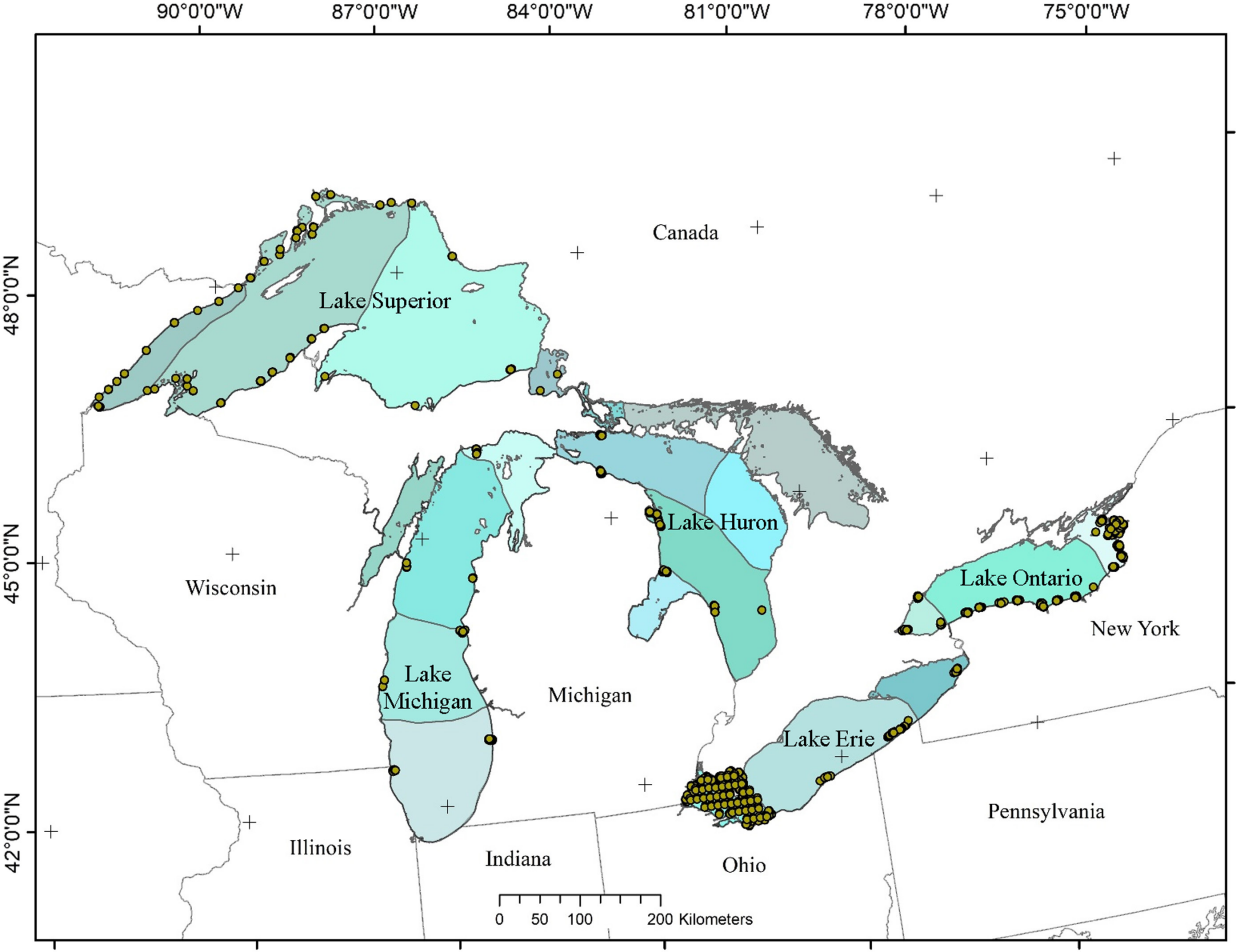
Map of fish collection within the nearshore zone of each of the Great Lakes and boundaries of the aquatic Lake units (fine gray lines subdividing the interior of each Lake). Each point represents multiple sample locations in that general vicinity that are indistinguishable at this map scale. The shaded polygons represent the aquatic Lake units (ALU) within the Great Lakes (McKenna & Castiglione, 2017).

This empirical analysis was possible because of extensive databases from throughout the Great Lakes collected by many people and agencies. Fish data were provided by the US Geological Survey and collaborators using standardized trawl collections throughout the Great Lakes (US Geological Survey, Great Lakes Science Center, 2018; Figure 1, Appendix 1). Abundance of each fish species at each trawl event location was effort‐standardized to number of fish per 1000 m^2^ of area swept by the trawl (catch per unit effort, CPUE) and ln‐transformed. There were 4332 nearshore fish assemblage samples with matching habitat and disturbance values throughout the Great Lakes (1540 from Lake Erie, 619 from Lake Huron, 293 from Lake Michigan, 1154 from Lake Ontario, and 837 from Lake Superior), and included 80 species, 35 of which occurred at least 100 times (Appendix 1). Collection station depth ranged from 0.55–15.55 m and of the 4332 collections, 1586 were from sites in depths ≤3 m. Although modeled because of their presence in other lakes, four species do not occur in Lake Erie (Cisco (*Coregonus artedi*), Bloater (*Coregonus hoyi*), Pygmy Whitefish (*Prosopium coulterii*), and Round Whitefish (*Prosopium cylindraceum*); Van Meter & Trautman, 1970; Scott & Crossman, 1973; Page & Burr, 1991); Cisco is a species of interest because of historic populations in Lake Erie (Oldenburg et al., 2007, Great Lakes Fishery Commission – Lake Erie Committee (glfc.org), September 2021). Each fish species was also classified according to its general habitat usage (pelagic vs. benthic or demersal).

Environmental and disturbance data were available for all fish observation locations throughout the Great Lakes proper and for every 30‐m spatial cell within Lake Erie. Data for 50 environmental variables, provided by the Great Lakes Aquatic Habitat Framework project (GLAHF) (Forsyth et al., 2016; Riseng et al., 2018; Wang et al., 2015), and the Great Lakes Regional Aquatic Gap Analysis Project, (McKenna et al., 2015; McKenna & Castiglione, 2010), were available to characterize “natural” habitat and aquatic conditions (Appendix 2). Anthropogenic disturbance data came from three sources (Allan et al., 2013; Hillyer, 1996; Wehrly et al., 2013) and consisted of the GLEAM index of Allan et al. (2013), the Wehrly index (Wehrly et al., 2013), and the Coastal Modification Index (Hillyer, 1996; Appendix 3). The GLEAM and Wehrly indices are composite indices of numerous stressors. The GLEAM index consists of 34 variables focused mostly on the open waters and coastline of each Great Lake. The Wehrly index consists of five synoptic variables affecting aquatic habitat within the watersheds emptying into each Great Lake and focused on nearshore stressors. The third disturbance variable was the coastline “protection” metric provided by the US Army Corps of Engineers (Hillyer, 1996), which is a measure of the extent of greatest shoreline modification projected out to each spatial cell in each lake. These disturbance measures represent the human perceptions of disturbances that are likely stressors for fish. These three stress factors were combined into composite variables in the partial canonical correspondence analysis (pCCA) described below.

### 2.3 Ordinations

**FIGURE 2:**
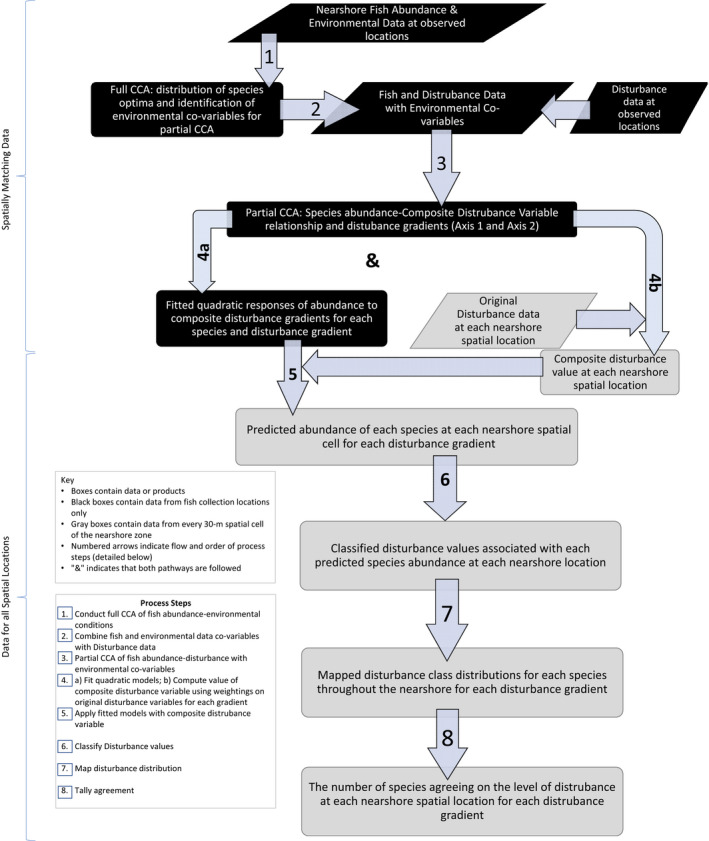
Flowchart summarizing the methodological process used to develop species‐specific models of abundance response to disturbance conditions. Each parallelogram represents data and each rectangle represents a product generated at each step of the process. Black polygons at the top of the diagram indicate steps accomplished using only data from locations where fish collections were made. Light Gray polygons at the bottom of the diagram indicate steps accomplished using disturbance data from every 30‐m spatial cell within the nearshore zone. Arrows indicate the direction of process flow and are numbered to indicate the order of process steps all flow pathways were followed and the “&” symbol indicates that both flow pathways of a given step must be executed. Information in the ovals explains the action taken at each step. CCA is canonical correspondence analysis.

**FIGURE 3:**
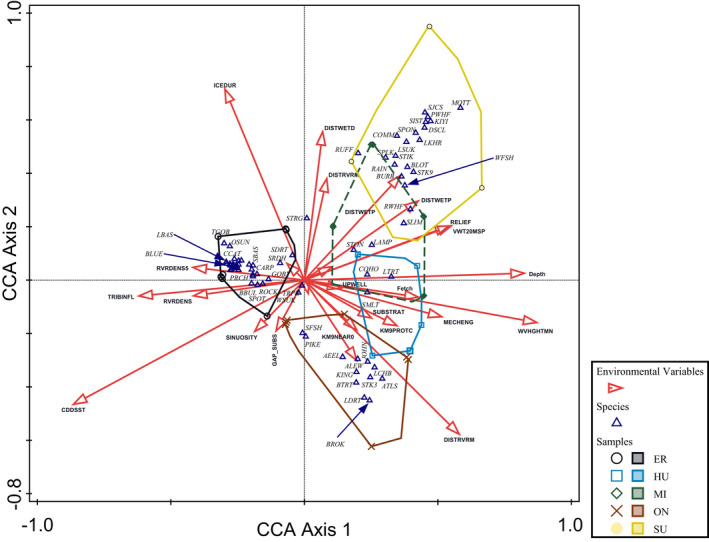
Hyperspace of the first two full canonical correspondence analysis (CCA) axes using all environmental variables identified as significant by forward selection testing. Species optima are indicated by open triangles. Samples within each great Lake are indicated by a different symbol and enclosed by color shaded polygons, Lake Erie with open black circles, Lake Huron with open green diamonds (dashed line), Lake Michigan with open blue squares, Lake Ontario with brown Xs, and Lake Superior with solid yellow circles. Red arrows show the direction in which each environmental variable increase in value within the plane depicted by the first two CCA axes (each of which is a variable that is a linear combination of all the environmental variables). Species codes are provided in Appendix 1 and environmental vector codes are provided in Appendix 2. A cluster of unlabeled species optima within the Lake Erie group include, BUFF, BULL, CCGF, CRAP, DRUM, EMRL, GIZZ, GLDF, GRED, LOGP, MIMC, MUSK, PUNK, QUIL, RHSP, SAND, SAUG, SBUF, SCHB, SHRH, SLMP, WALL, WBAS, WCRP, WHPR, and YBUL. The optima for bluegill (BLUE), brook trout (BROK), largemouth bass (LBAS), and Lake whitefish (WFSH) are indicated by blue arrows.

Our objectives were accomplished by a methodological process that began with ordination followed by regression, classification, and GIS mapping (Figure 2). Multivariate methods help reduce complex relationships among multiple species and with their environments to fewer, simpler relationships (Pielou, 1977). These provide insight into the influence of and preferences for various types of conditions by each species of a biotic community (e.g. McKenna, 2013; McKenna & Castiglione, 2010; Kovalenko et al., 2018; ter Braak, 1995). Correspondence analysis uses unimodal responses to identify important patterns of differences and similarities in species optimal conditions. In the canonical correspondence analysis (CCA) used here, the ordination was constrained to use combinations of the habitat variables to build the best composite variable that explained the variation in the fish abundance data. We used the CANOCO program to conduct CCA with forward selection of each environmental variable, using a permutation test for significance (99 permutations), to identify each species' preferred environmental conditions (ter Braak & Smilauer, 2012). The full CCA used spatially matched ln‐transformed fish CPUE and environmental data for all Great Lakes nearshore zones (ter Braak, 1995). The forward selection procedure identified those environmental variables that significantly affected fish abundance. The full CCA identified 26 of the 50 environmental variables as influential (all inflation factors were <6.6, Figure 3, Appendix 2, Table S1). Weighted linear combinations of those variables were used to construct composite environmental indices represented by each ordination axis (each axis is composed of all 26 environmental variables, but with different weightings), with the first axis explaining the most variability within the data and subsequent orthogonal axes explaining additional portions of the remaining variation. A triplot diagram shows the clustering of samples and associations of each species' optimal conditions with the environmental variable gradients and each Great Lake (Figure 3).

**FIGURE 4:**
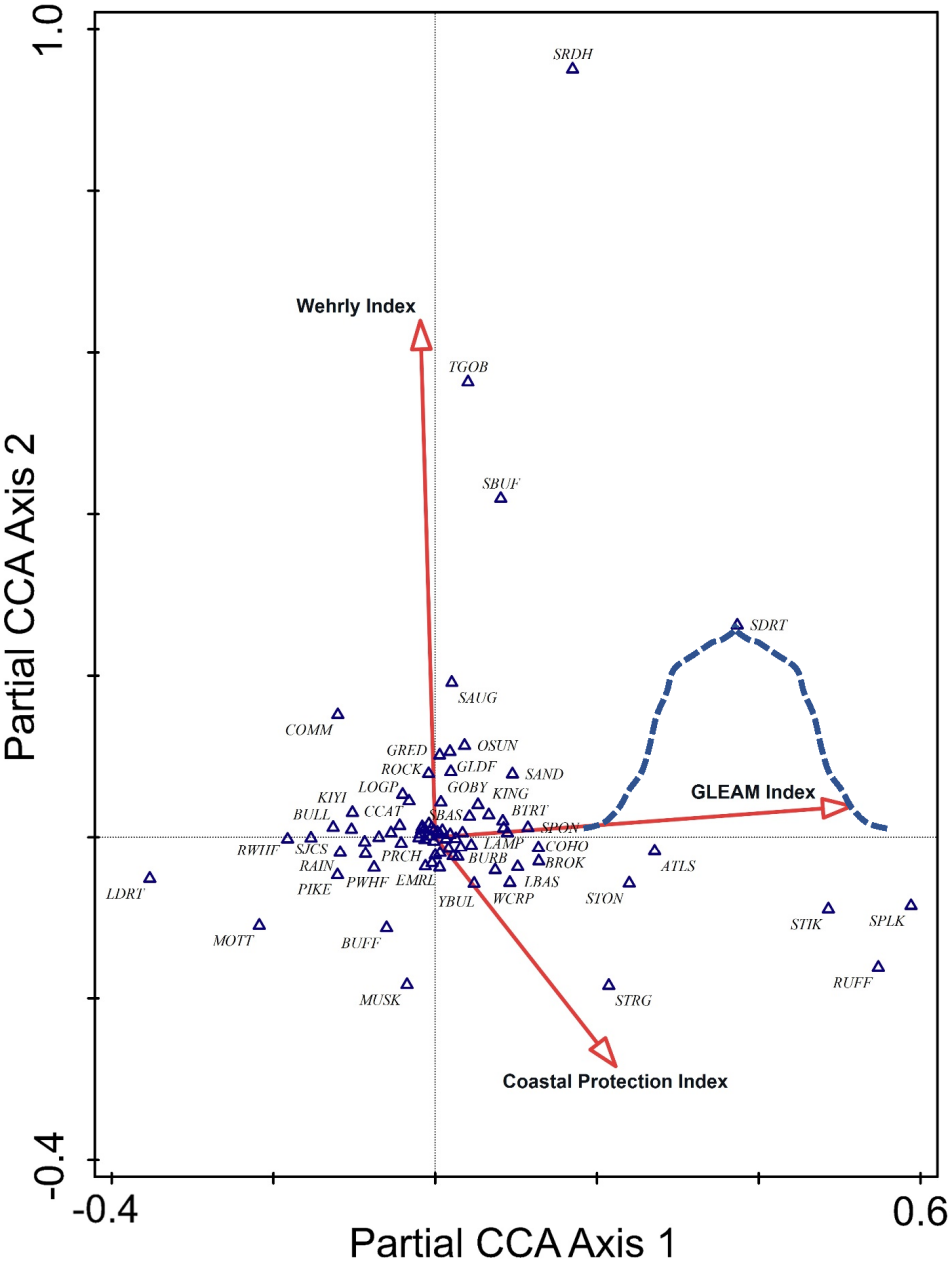
Hyperspace of partial canonical correspondence analysis (pCCA) diagram of first two ordination axes showing distribution of locations of species optima (triangles) and disturbance vectors indicating relative influence and direction of increasing effect within the hyperspace (arrows), after influence of habitat variables were removed. An example unimodal response for eastern sand darter as a function of the Axis 1 composite is shown by a dashed line. Species codes are provided in Appendix 1. The unlabeled species optima include, AEEL, ALEW, BBUL, BLOT, BLUE, CARP, CCGF, CRAP, DRUM, DSCL, GIZZ, JOHN, LCHB, LKHR, LSUK, LTRT, MIMC, PUNK, QUIL, RHSP, SCHB, SFSH, SHRH, SIST, SLIM, SLMP, SMLT, SPOT, STK3, STK9, TRPR, WALL, WBAS, WFSH, WHPR, and WSUK.

We then used partial CCA ordination to parse out the effects of the natural environmental factors and isolate the effects of the disturbance factors on fish abundances; the 26 influential environmental variables identified in the full CCA were co‐variables with the three anthropogenic disturbance variables. As with the full ordination, the partial CCA process used weighted linear combinations of the three disturbance variables to construct composite disturbance variables represented by each axis, hereafter called disturbance gradients. We used the first two axes (i.e., disturbance gradients) because they explain the most variability (Figure 4).

### 2.4 Quadratic model fit

**FIGURE 5:**
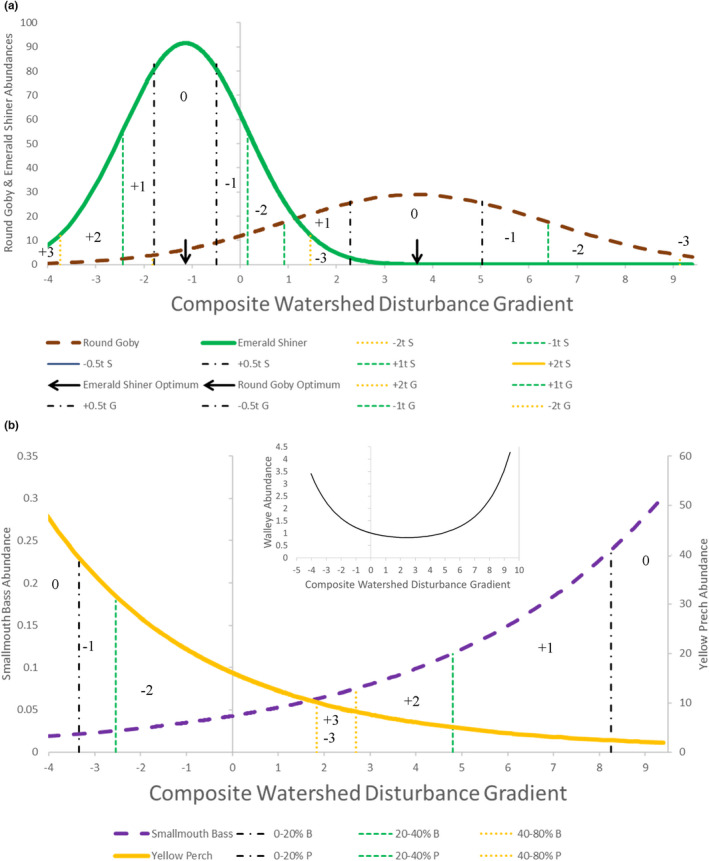
Illustration of quadratic response models and disturbance class units showing selected curves of predicted fish abundances as functions of the watershed disturbance gradient and lines marking boundaries of class units. The thin, solid vertical line with tic marks indicates the “average” disturbance gradient value of zero and is the ordinate for fish abundances, except for yellow perch (*Perca flavescens*). (a) tolerance classes of two different unimodal responses where the solid curve (green) represents predicted emerald shiner (*Notropis atherinoides*) abundance catch per unit effort (CPUE) and the dashed curve (brown) represents predicted round Goby (*Neogobius melanostomus*) abundance (CPUE). Vertical lines indicate disturbance values three tolerance units from the optimum value (yellow dotted lines), two tolerance units from the optimum (green dashed lines), and one tolerance unit from the optimum (black dash‐dot lines); legend labels for emerald shiner tolerance boundary lines are followed by an “S” and those for round Goby are followed by a “G”. Note that the −3 *t* line for round Goby is nearly coincident with the −0.5 *t* line for emerald shiner and that the labels for the round Goby +2 and +3 disturbance classes overlap with emerald shiner classes and are not shown. Optimum disturbance occurred where predicted abundance was maximum and is indicated by a black arrow. Disturbance class labels are provided within each interval (+3, +2, +1, 0, −1, −2, −3), with negative code values indicating perceived degradation and positive values indicating hyperoptimality. (b) Percentile classes of two different monotonic responses, where the solid curve (yellow) represents predicted yellow perch abundance (CPUE) and the dashed curve (purple) represents predicted smallmouth bass (*Micropterus dolomieu*) abundance (CPUE). Vertical lines indicate disturbance values at the 80‐percentile class (yellow dotted lines), the 40‐percentile (green dashed lines), and the 20‐percentile (black dash‐dot lines); legend labels for smallmouth bass percentile boundary lines are followed by a “B” and those for yellow perch are followed by a “P”. Disturbance class labels are the same as for the unimodal responses; +3 is associated with smallmouth bass and −3 is associated with yellow perch. The inset chart shows the parabolic response for walleye abundance as a function of watershed disturbance.

Predictive quadratic models were developed from these simplified multivariate relationships of species response to disturbance gradients in the two partial CCA unimodal models. All species included in the nearshore dataset (80 species) were included in the ordinations. However, most species were rare and to help ensure detectable responses to habitat and disturbance conditions, only species which occurred at least 100 times in the dataset (35 species) were used to develop the quadratic response models. We used the CANOCO program to fit those quadratic models of the response of fish abundance (ln[CPUE]) for each species, separately to each composite disturbance gradient, assuming a Poisson distribution and using an *F*‐test for model significance at the *α* = 0.05 level (see ter Braak & Smilauer, 2012, section 5.4.4). The optimum is the disturbance value associated with the maximum predicted abundance. The tolerance is a measure of the spread of a unimodal curve along the disturbance gradient. Together these describe the shape of unimodal response patterns, the same characteristics as that of a Gaussian curve (Jongman et al., 1995; ter Braak, 1995; See Figure S1). Monotonic increasing or decreasing response curves can also result from quadratic model regression (Figure 5). If the second order term was not significant or the linear form of the model (i.e., model without the second order term) contained more information than the quadratic form (i.e., greater Akaike Information Criterion [AIC] value), the linear model was selected.

These predicted species abundance patterns reflected fish response to disturbed conditions. Composite disturbance index values for each 30‐m spatial cell within Lake Erie's nearshore zone were computed by applying the weightings of the linear combinations for each disturbance gradient (i.e., partial CCA axis) to the values of the three original disturbance variables at each spatial location. These composite index values formed the independent variable data for quadratic model predictions and species‐specific abundance predictions were then made for each Lake Erie nearshore zone spatial unit (cell) for each disturbance gradient. To simplify interpretation and comparison of disturbance conditions between species and among geographic areas, predicted disturbance values were classified according to the number of relative deviation units of those values from the optimum disturbance gradient value (i.e., at maximum abundance). Unimodal responses of fish abundance were placed into associated disturbance classes of multiple tolerance units (*t*) and coded for display and quantification purposes as +3 (<−2 *t*), +2 (<−1 *t* to ≥−2 *t*), +1 (<−0.5 *t* to ≥−1 *t*), 0 (≤0.5 *t* to ≥−0.5 *t*), −1 (>0.5 *t* to ≤1 *t*), −2 (>1 *t* to ≤2 *t*), −3 (>2 *t*). This scale highlights small negative code values (e.g., −3) as highly degraded, large positive numbers as hyperoptimal (defined here as less degraded than preferred), and 0 as preferred conditions (Figure 5). For example, an abundance value within ½ tolerance unit away from the predicted maximum would be associated with a disturbance level within ½ tolerance unit from the optimum disturbance level and would be given a disturbance class label of 0. An abundance value associated with a disturbance gradient value more than 2 tolerance units greater (i.e., more disturbed) than the optimal value was labeled −3. This tolerance unit scale divides the area under a symmetric unimodal curve of predicted fish abundance into four units on either side of the optimum disturbance value (splitting the 0 class into ½ *t* units). These relative measures of deviation from the optimum disturbance value were necessary because maximum abundance values and, thus, optimal disturbance values could occur anywhere along the disturbance gradients and differed by species. For best comparison, monotonic response model predictions of abundance were assigned to four similar disturbance classes, based on quintiles of the areas under those curves from the disturbance value associated with the maximum abundance value as follows: <20%, 20%–40%, 40%–80%, and >80% (Figure 5b). These indicate degrees of degradation for species whose abundance decreased with increasing disturbance gradient values and the degree of hyperoptimality for species whose abundance increased with increasing disturbance gradient values. The associated disturbance class labels were 0, 1, 2, or 3 and were positive or negative depending on whether the curve was increasing or decreasing. These classifications provide comparable measures of the degree of disturbance for each fish species in response to each disturbance gradient in each spatial unit.

### 2.5 Disturbance distributions

Classified model predictions of disturbance at each spatial location within the Lake Erie nearshore zone were then used in a GIS to determine spatial distributions of disturbance. A separate map layer was generated for each species for each of the two disturbance gradients. Each spatial cell was colored coded according to the associated disturbance class label for a given species. Yellow Perch (*Perca flavescens*) and Smallmouth Bass (*Micropterus dolomieu*) were selected to illustrate how species may respond differently to levels and changes in anthropogenic disturbances because of their clearly opposing responses.

### 2.6 Disturbance level agreement

Overlay of these species‐specific distributions allowed us to count the number of species whose predicted abundances were classified into the same disturbance class (i.e., in agreement about the level of disturbance). All species‐specific maps of disturbance were geographically aligned such that the disturbance class value for each species at each 30‐m spatial location of the nearshore zone were stacked one on top of the other. Then, for each disturbance class, a count was made of the number of species in the stack whose predicted disturbance fell into that class at that location. Thus, the number of species associated with the most highly disturbed conditions (class −3) was recorded for a given spatial location. Then the number associated with moderately disturbed conditions (class −2) at that same location was recorded, and so forth for each disturbance class. The process was then repeated for every 30‐m spatial location. These counts of species agreement were made separately for each disturbance gradient and mapped to show the spatial distribution of high and low agreement about disturbance throughout Lake Erie's nearshore zone. The predictive models developed for the four species that do not exist in Lake Erie, but are present in other Great Lakes, were excluded from the counts of agreement for Lake Erie.

## 3 RESULTS

Environmental and anthropogenic disturbance index values were available for all of the >53 million 30‐m spatial cells throughout all of the Great Lakes nearshore zones. Anthropogenic disturbances were not uniformly distributed throughout the nearshore zones (Figure S2; see Allan et al., 2013; Riseng et al., 2018; Wehrly et al., 2013 for distribution maps of the entire Great Lakes Region). In Lake Erie, the highest values (i.e., interpreted as most disturbed) of the predominately in‐lake GLEAM disturbance index occurred in sections along the south shore and Buffalo, New York area, with many other areas having less disturbed conditions (Figure S2a). The highest values of the predominately watershed derived Wehrly disturbance index were concentrated in the westernmost portion of the lake (Figure S2b). Shoreline modifications also varied spatially, with the most extensive modifications in scattered patches along the southern coast of the lake and a few other areas (Figure S2).

### 3.1 Regionwide ordination

The full CCA ordination provided indications of the effects of “natural variability” on distributions and abundances of the fishes in the Great Lakes. The ordination explained 18.6% of total variation and >75% of fitted variation with the first two canonical axes (Table 1). Those CCA axes were dominated by the influences of cumulative degree‐days (measured from mean daily surface water temperature with base 0°C), distance to nearest tributary mouth, mean summer wave height, ice duration, and water depth (Figure 3, Appendix 2). Species optima (shown by open triangles in Figure 3) indicate the hyperspace location where environmental conditions were associated with the highest abundance values for each species, i.e., optimum conditions. For example, Largemouth Bass (*Micropterus salmoides*) and Bluegill (*Lepomis macrochirus*) have similar (but not the same) habitat optima, while Lake Whitefish (*Coregonus clupeaformis*) habitat conditions are quite different from Brook Trout (*Salvelinus fontinalis*) habitat. There was relatively strong separation by Great Lake based on distinct groups of species optima and associated conditions, with a clear gradient from Lake Ontario (negative values) to Lake Superior (positive values) along the second axis, but with Lake Erie being most distinct and located in the negative portion of the first axis and at approximately average values of the second axis.

**TABLE 1:** Summary of full and partial canonical correspondence analysis (pCCA) ordination results using all available habitat and disturbance variables and matching fish abundances throughout the Great Lakes within the nearshore zone. The pCCA ordination used explanatory environmental variables of the full CCA as co‐variables and disturbance indices as explanatory variables

Analysis	Axis	Total variation	Explained variation	Adjusted explained variation	Eigenvalues	Cumulative explained variation	Pseudo‐canonical correlation	Cumulative explained fitted variation	Axis weighting
Full CCA	All	7.77597	18.60%	18.11%					
	Axis 1				0.7062	9.08%	0.9604	48.82%	
	Axis 2				0.3813	13.99%	0.8652	75.18%	
	Axis 3				0.0662	14.84%	0.6207	79.76%	
	Axis 4				0.0561	15.56%	0.5814	83.64%	
Partial CCA	All	6.33966	0.56%	0.49%					
	Axis 1				0.019	0.3%	0.3459	53.86%	53.86%
	Axis 2				0.011	0.47%	0.2947	84.34%	30.48%
	Axis 3				0.0055	0.56%	0.2299	100%	
	Axis 4				0.2846	5.05%	0		

### 3.2 Disturbance ordination

Partial CCA filtered out the natural variability and determined how the remaining variability was affected by disturbance factors (GLEAM, Wehrly, and Coastal Modification) (Anderson & Gribble, 1998; Esselman et al., 2011), revealing the effects of anthropogenic disturbances (as defined by our disturbance indices and coastal modification metric) on fish distributions and identifying “optimal” disturbance conditions for each species (Figure 4). The first two axes of the partial CCA explained 0.56% of total remaining variation, but >84% of the association between disturbance and fish abundances (Table 1). Forward selection included all three disturbance variables (inflation factors were <4.3). Values of the 1st CCA axis were determined by,

Axis1=1.9675G–0.1488W–0.0684S,

and by

Axis2=0.1937G+1.4048W–0.3321S

for values of the 2nd CCA axis (Table 2), where *G* is the value of the GLEAM index, *W* is the value of the Wehrly index, and *S* is the value of the Coastal Modification index.

**TABLE 2:** Composite variable weightings by partial canonical correspondence analysis (pCCA)axis. GLEAM is the Great Lakes environmental assessment mapping disturbance index (Allan et al., 2013) and Wehrly is the watershed disturbance index (Wehrly et al., 2013), and coastline modification is the shoreline disturbance index (Hillyer, 1996).

Index	Axis 1	Axis 2
GLEAM	1.9675	0.1937
Wehrly	−0.1488	1.4048
Coastline Modification	−0.0684	−0.33211

As with the full CCA ordination diagram, the lengths and directions of the arrows in Figure 4 indicate that the Wehrly index (watershed effects) focused in the nearshore zone had the most influence (longest vector), but was very closely aligned with Axis 2 (which explains less variation then Axis 1), while the GLEAM index (essentially open lake and coastal effects), the second longest vector, is closely aligned with Axis 1. The coastal modification index (KM9PROTC) had the least influence, but contributed approximately equally to each CCA axis. Because the first CCA Axis was so strongly dominated by the GLEAM index and the second CCA Axis was strongly dominated by the Wehrly index (Table 2, Figure 4), we hereafter refer to these disturbance gradients as in‐lake disturbance and watershed disturbance, respectively. The clusters of sample points showed no separation by lake. The ordination used only locations where matching fish, habitat, and disturbance values existed. However, values of each of the three original disturbance variables were available for every 30‐m spatial cell within the nearshore zone, and because each disturbance gradient is a weighted combination of those three original disturbance variables, the values of disturbance were computed for each spatial cell and subsequently used in fish prediction models. Each species optimum represents the peak of a unimodal response to changes in fish abundance along each ordination axis, at which a specific combination of disturbance conditions occurs (based on the three variables; Figure 4).

#### 3.2.1 Spatial distribution of disturbance

**FIGURE 6:**
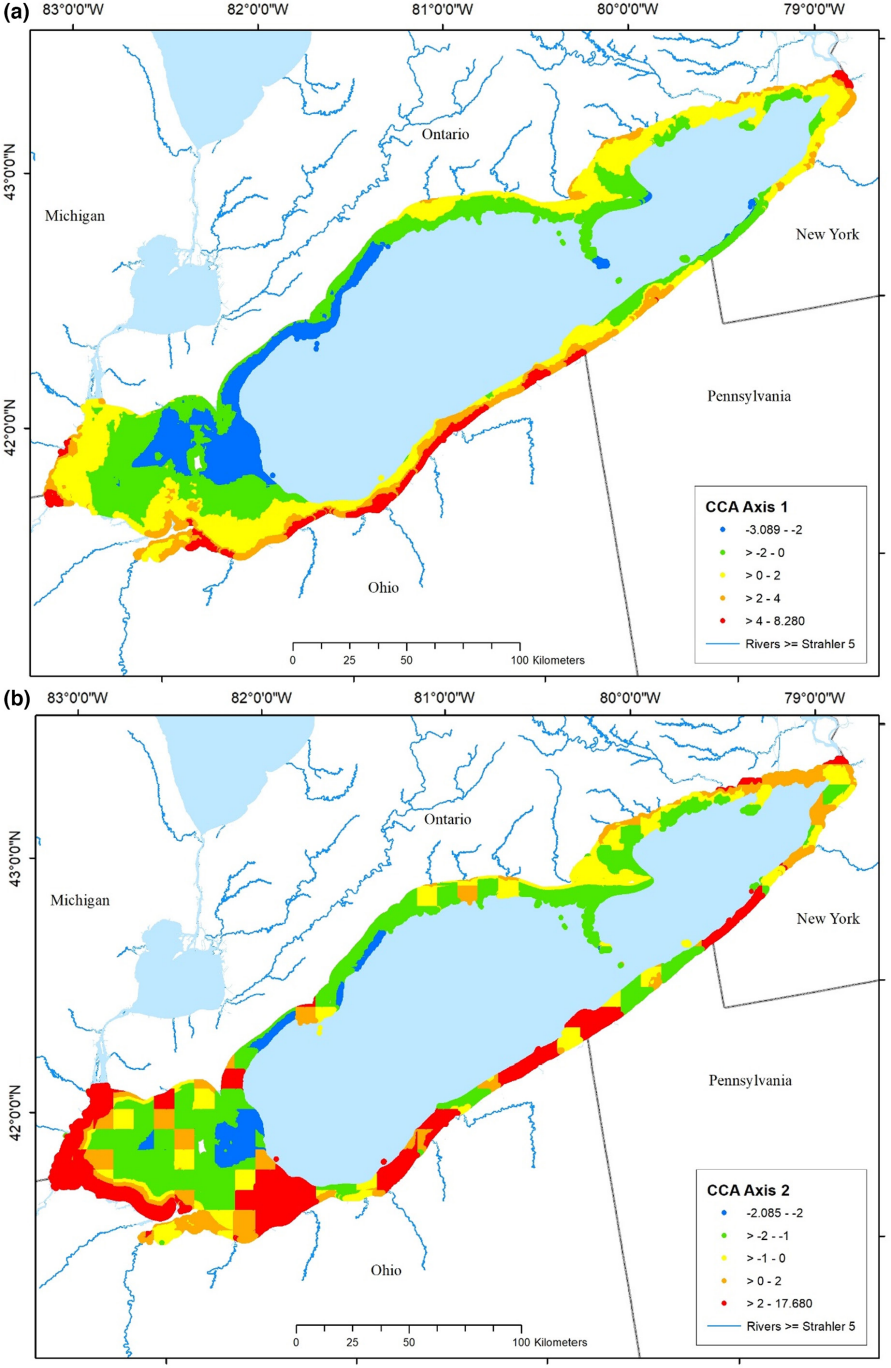
Spatial distributions of composite partial canonical correspondence analysis (pCCA) disturbance variables for (a) Axis 1 (in‐lake dominated) and (b) Axis 2 (watershed dominated) in Lake Erie.

Given the dominance of each ordination axis by one of the disturbance indices, it is no surprise that the distributions of disturbance, based on the composite ordination variables, resemble aspects of the three original disturbance variables (Figure 6). Values of the in‐lake disturbance gradient were highest closest to shore in Maumee Bay, Ohio, near Buffalo, NY, near the mouth of the Raisin River, Michigan, and in a large section of the south shore from Sandusky Bay to the Pennsylvania border with Ohio. Western sections of the north shore and offshore areas of the western ALU were least disturbed. The second CCA Axis (Watershed‐dominated gradient) showed most disturbed areas in a band along the western end of the lake from the Detroit River to Cedar Point, OH and in large patches along the south shore and a few other scattered areas; other areas experienced moderate to low disturbance.

### 3.3 Responses to disturbance

Quadratic models fit responses of fish abundance to each disturbance gradient well for most of the 35 species that occurred at least 100 time in the dataset (Table 3). Of the 70 possible models, no model could be successfully fitted to three species, Burbot (*Lota lota*) and Bluegill in response to watershed disturbance, and Common Carp (*Cyprinus carpio*) in response to in‐lake disturbance. Seven of the remaining models were significant (*p* ≤ .05) as linear, but not quadratic models; all linear models had negative slopes, except that for Smallmouth Bass (Table 4). All other models were significant (*p* ≤ .05) quadratic models.

**TABLE 3:** Quadratic model response pattern, Optimum, tolerance, and each parameter value (significance *p* values) for each species and disturbance gradient. Model column values indicate the Akaike information criterion (AIC)‐determined best model, with L representing linear and Q representing quadratic; NULL indicates that no model could be successfully fit to the data. The *F*‐stat column provides the *F*‐test statistic for significance of the associated model. A double asterisk (**) after an *F* statistic indicates an associated probability of <0.001. A single asterisk (*) after a parameter value (constant, X term, or X2 term) indicates a significant *t*‐test (DF = 4329, |*t*| > 2.81) for that parameter of the associated model. Response pattern indicates pattern manifested by the fitted model, “I” is monotonically increasing, “D” is monotonically decreasing, “O = x” is optimum with maximum within 0.05 units of the disturbance mean, “O < x” is optimum with maximum <0.05 disturbance units less than the mean, “O > x” is optimum with maximum more than 0.05 disturbance units greater than the mean, and “P” is concave up parabolic. Boldface codes indicate those species with a decreasing response pattern or an unimodal response pattern with optimum located at a disturbance gradient value <‐0.5. Underlined codes indicate those species an increasing response pattern or an unimodal response pattern with optimum located at a disturbance gradient value <‐0.5. Italic codes indicate those species with an unimodal response pattern with optimum located at a disturbance gradient value < ‐0.5 and < ‐0.5. General habitat use (B = benthic or demersal, P = pelagic, U = unknown) and native status (N = native, E = exotic, A = absent, X = extirpated, and U = unknown) for Lake Erie shown parenthetically after each species name. Species code name followed by “a” indicates unimodal model with optimum beyond the range of the axis. The b superscript indicates a non‐significant linear model, but significant quadratic model. Non‐significant *t* values are shown after each associated parameter estimate. In all cases model DF were 1, 2, or 3 (quadratic being 3) and respective residual DF were 4331, 4330, or 4329. A ^a^ after a species codes indicates that the species optimum was outside the range of the ordination axis. Species codes are defined in Appendix 1.

Disturbance gradient	Species (classes)	Model	Response pattern	*F*‐stat (*p*)	Constant1	x_term1	x^2^_term1	Optimum	Tolerance
In‐Lake	**GOBY (B, E)**	Q	Decreasing	46.68**	2.44206*	−0.164*	0.017*	–	–
	**LKHR (P, X)**	Q	Decreasing	2081.36**	−0.311699*	−0.512*	0.243*	–	–

**PWHF (U, A)**	L	Decreasing	16.29**	−2.45238*	−0.2*	–	–	–

**RWHF (U, A)**	L	Decreasing	193.73**	−3.75738*	−1.077*	–	–	–

**STK9 (P, N)**	Q	Decreasing	14.72**	2.0211*	−0.591*	0.013*	–	–

**WFSH (B, N)**	Q	Decreasing	1161.93**	−0.709247*	−0.54*	0.232*	–	–

**JOHN (B, N)**	Q	Optimum < mean	126.20**	0.491404*	−0.484*	−0.101*	−2.39	2.22

**CCAT (B, N)**	Q	Optimum < mean	18.53**	−1.55261*	−0.216*	−0.104*	−1.04	2.19

*PRCH (B, N)*	Q	Optimum < mean	15976.47**	3.11589*	−0.335*	−0.546*	−0.306	0.957

*BLOT (B, A)*	Q	Optimum < mean	2180.12**	0.788259*	−0.481*	−0.822*	−0.293	0.78

*SPOT (P, N)*	Q	Optimum < mean	3875.07**	2.4115*	−0.044*	−0.262*	−0.083	0.0134

*WALL (B, N)*	Q	Optimum = mean	606.62**	0.301618*	0.012^b^ (*t* = 0.610)	−0.321*	0.019	1.25

*QUIL (B, N)*	Q	Optimum = mean	15.84**	−2.43089*	0.025^b^ (*t* = 0.350)	−0.261*	0.048	1.38

*WBAS (P, N)*	Q	Optimum > mean	15209.92**	2.44858*	0.155*	−1.247*	0.062	0.633

*DRUM (B, N)*	Q	Optimum > mean	7587.96**	1.84445*	0.16*	−1.033*	0.077	0.696

*SCHB (P, N)*	Q	Optimum > mean	682.01**	−0.741045*	0.358*	−1.411*	0.127	0.595

*WHPR (P, E)*	Q	Optimum > mean	76169.33**	4.45914*	0.181*	−0.629*	0.144	0.892

*TRPR (B, N)*	Q	Optimum > mean	3686.84**	3.22384*	0.039*	−0.134*	0.145	1.93

*GIZZ (P, N)*	Q	Optimum > mean	2676.06**	1.96329*	0.122*	−0.263*	0.232	1.38

*BLUE (B, N)*	Q	Optimum > mean	202.37**	−2.19838*	1.026*	−1.888*	0.272	0.515

*PUNK (B, N)*	Q	Optimum > mean	665.00**	−1.07937*	1.206*	−2.22*	0.272	0.475

*EMRL (P, N)*	Q	Optimum > mean	41899.43**	4.3272*	0.238 *	−0.261*	0.455	1.38
	MIMC (P, N)	Q	Optimum > mean	575.50**	−1.5389*	1.946*	−1.66*	0.586	0.549

SBAS (B, N)	Q	Optimum > mean	14.30**	−3.06389*	0.629*	−0.167*	1.88	1.73

SPON ^a^ (B, N)	Q	Increasing	6.12 (0.013)	−2.17353*	0.885*	−0.046 (*t* = −2.406)	9.6	–

ALEW ^a^ (P, E)	Q	Increasing	100.01**	5.41749*	0.339*	−0.005*	34.52	–

LSUK (B, N)	Q	Increasing	70.77**	−2.7163*	0.051 (*t* = 1.185)	0.163*	–	–

SMLT (P, E)	Q	Increasing	31722.37**	5.0608*	0.163 *	0.087*	–	–

WSUK (B, N)	Q	Increasing	10.00 (0.002)	−2.19353*	0.265*	0.06*	–	–

BURB (B, N)	Q	Parabolic	74.04**	−3.59879*	−0.091^b^ (*t* = −1.701)	0.221*	–	–

DSCL (B, N)	Q	Parabolic	25.67**	−2.43205*	−0.223*	0.111*	–	–

LTRT (B, N)	Q	Parabolic	23.51**	−1.40258*	0.02 (*t* = 0.757)	0.067*	–	–

SLIM (B, N)	Q	Parabolic	120.82**	0.0502333*	−0.075a (*t* = −6.293)	0.098*	–	–

STK3 (P, E)	Q	Parabolic	4151.00**	1.79766*	−0.217*	0.15*	–	–

CARP (B, E)	NULL	–	–	–	–	–	–	–
Watershed	Species (Classes)	Model	Response Pattern	F‐stat (p)	constant2	x_term2	x^2^_term2	Optimum	Tolerance

**ALEW (P, E)**	L	Decreasing	2381.94**	5.468*	−0.049*	–	–	–

**BLOT (B, A)**	L	Decreasing	6909.12**	−0.011 (*t* = −0.708)	−1.014*	–	–	–

**DRUM (B, N)**	Q	Decreasing	486.48**	1.171*	−0.461*	0.05*	–	–

**PRCH (B, N)**	Q	Decreasing	5.16 (0.023)	2.777*	−0.258*	0.003 (*t* = 2.321)	–	–

**PUNK (B, N)**	L	Decreasing	40.07**	−1.829 *	−0.274*	–	–	–

**QUIL (B, N)**	L	Decreasing	11.12**	−2.664*	−0.216*	–	–	–

**WHPR (P, E)**	Q	Decreasing	3082.92**	4.006*	−0.252*	0.028*	–	–

**WSUK (B, N)**	L	Decreasing	11.34**	−2.066*	−0.159*	–	–	–

**WFSH (B, N)**	Q	Optimum < mean	300.41**	−0.393*	−1.295*	−0.21*	−3.09	1.54

**SCHB (P, N)**	Q	Optimum < mean	11.68**	−1.404*	−0.466*	−0.076*	−3.05	2.56

**EMRL (P, N)**	Q	Optimum < mean	18190.66**	4.13*	−0.679*	−0.298*	−1.14	1.3

**PWHF (U, N)**	Q	Optimum < mean	36.51**	−2.343*	−0.707*	−0.407*	−0.868	1.11

**WBAS (P, N)**	Q	Optimum < mean	1059.55**	1.911*	−0.324*	−0.197*	−0.821	1.59

**MIMC (P, N)**	Q	Optimum < mean	51.16**	−1.824*	−0.57*	−0.368*	−0.774	1.17

**LKHR (P, X)**	Q	Optimum < mean	1079.29**	0.4*	−1.056*	−0.778*	−0.679	0.802

*SMLT (P, N)*	Q	Optimum < mean	78652.40**	5.383*	−0.285 *	−0.495*	−0.287	1

*RWHF (U, N)*	Q	Optimum < mean	16.89**	−2.892*	−0.371^b^ (*t* = −2.294)	−0.829*	−0.224	0.777

*STK3 (P, N)*	Q	Optimum < mean	47186.09**	3.024*	−2.266*	−10.31*	−0.11	0.22

*DSCL (B, N)*	Q	Optimum = mean	36.71**	−1.993*	−0.057^b^ (*t* = −0.577)	−0.774*	−0.037	0.804

*STK9 (P, N)*	Q	Optimum = mean	1278.74**	2.309*	0.001 (*t* = 0.175)	−0.124*	0.005	2

*LTRT (B, N)*	Q	Optimum = mean	101.97**	−1.043*	0.077^b^ (*t* = 1.246)	−0.828*	0.047	0.777

*JOHN (B, N)*	Q	Optimum > mean	7573.60**	1.327*	1.901*	−6.954*	0.137	0.268

*SLIM (B, N)*	Q	Optimum > mean	877.17**	0.385*	0.436*	−0.611*	0.357	0.905

TRPR (B, N)	Q	Optimum > mean	4615.44**	3.154*	0.229*	−0.114*	1.01	2.09

SPON (B, N)	Q	Optimum > mean	175.09**	−1.75*	0.935*	−0.422*	1.11	1.09

LSUK (B, N)	Q	Optimum > mean	46.21**	−2.365*	0.531*	−0.237*	1.12	1.45

GOBY (B, N)	Q	Optimum > mean	3533.33**	2.471*	0.489*	−0.067*	3.66	2.74

CCAT (B, N)	Q	Increasing	7.06 (0.008)	−1.707*	0.185*	0.015 (*t* = 2.70)	–	–

SBAS (B, N)	L	Increasing	16.60**	−3.149*	0.208*	–	–	–

CARP (B, E)	Q	Parabolic	5.36 (0.005)	−3.493*	−0.179^b^ (*t* = −2.072)	0.045*	–	–

GIZZ (P, N)	Q	Parabolic	1094.84**	1.684*	−0.209*	0.039*	–	–

SPOT (P, N)	Q	Parabolic	529.56**	2.171*	−0.069*	0.022*	–	–

WALL (B, N)	Q	Parabolic	151.82**	0.002 (*t* = 0.126)	−0.166*	0.034*	–	–

BLUE (B, N)	NULL	–	–	–	–	–	–	–

BURB (B, N)	NULL	–	1.59 (0.207)	–	–	–	–	‐‐

^a^ These species have optima outside the range of the Axis.

**TABLE 4:** Type of response of each species to each disturbance gradient. Parabolic models were excluded. See Appendix 1 for species scientific names. The pCCA is partial canonical correspondence analysis.

Response type	Disturbance gradient	
	In‐lake dominated (pCCA Axis 1)	Watershed dominated (pCCA Axis 2)
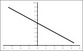 Linear decreasing	Pygmy Whitefish, Round Whitefish	Alewife, Bloater, Pumpkinseed, Quillback, White Sucker
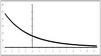 Monotonic decreasing	Round Goby, Cisco, Nine‐spine Stickleback, Lake Whitefish	Freshwater Drum, Yellow Perch, White Perch
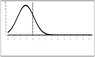 Optimum < mean	Channel Catfish, Johnny Darter	Lake Whitefish, Silver Chub, Emerald Shiner, Pygmy Whitefish, White Bass, Mimic Shiner, Cisco
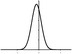 Optimum near mean	Yellow Perch, Bloater, Spottail Shiner, Walleye, Quillback, White Bass, Freshwater Drum, Silver Chub, White Perch, Trout‐perch, Gizzard Shad, Bluegill, Pumpkinseed, Emerald Shiner	Rainbow Smelt, Round Whitefish, Three‐spine Stickleback, Deepwater Sculpin, Nine‐spine Stickleback, Lake Trout, Johnny Darter, Slimy Sculpin
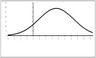 Optimum > mean	Mimic Shiner, Smallmouth Bass	Trout‐perch, Spoonhead Sculpin, Longnose Sucker, Round Goby
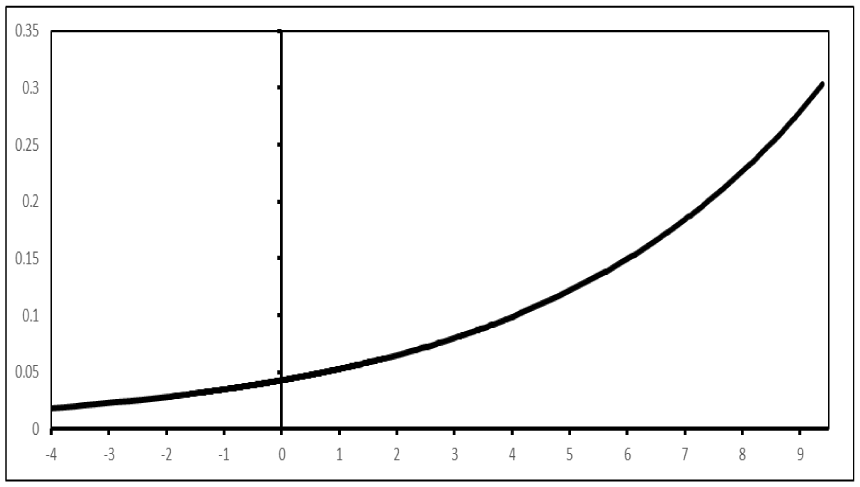 Monotonic increasing	Alewife, Longnose Sucker, Rainbow Smelt, Spoonhead Sculpin, White Sucker	Channel Catfish
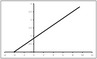 Linear increasing		Smallmouth Bass

Quadratic models produced four patterns of fish abundance response to the two disturbance gradients, monotonic decreasing, monotonic increasing, unimodal, and concave up parabolic (Figure 5, Table 4). Monotonic decreasing models indicated that maximum fish abundance occurred at the smallest observed disturbance gradient value (or less) and decreased with increasing disturbance values. Monotonic increasing models indicated that maximum fish abundance occurred at the largest observed disturbance gradient value and decreased with decreasing disturbance values. The unimodal models indicate that fish abundance was maximal at intermediate disturbance values. Thus, lower abundances occurred at both higher and lower disturbance values than that associated with the optimum. Parabolic models may be truncated bimodal curves and could suggest ecological displacement from optimum conditions due to competition (Fresco, 1982), but that investigation is beyond the scope of this study; species with parabolic response models for a given disturbance gradient were excluded from further analysis (Table 3).

The majority of response patterns were unimodal within each disturbance gradient (62% of responses to in‐lake disturbance and 65% of responses to watershed disturbance; Table 3). Those models were divided into three classes for this analysis, optima near the mean (within ½ disturbance gradient unit) disturbance gradient value (defined here as a disturbance value of zero), those with optima less than the mean class, and those with optima greater than the mean class (Table 4).

**FIGURE 7:**
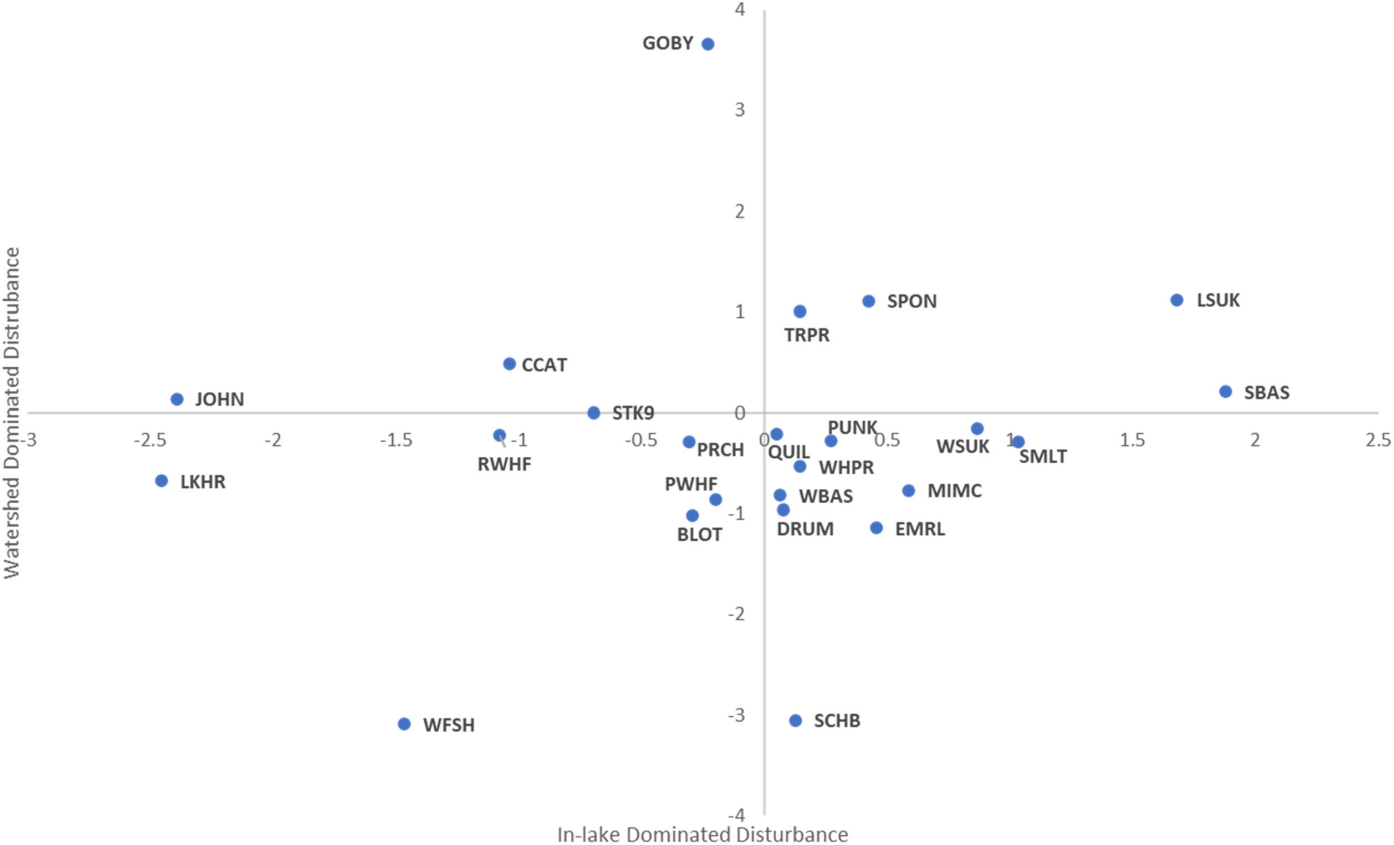
Relative disturbance preferences, based on optimal (unimodal model) or maximal slope (monotonic) disturbance value associated with each species for which a successful non‐parabolic model was available for both disturbance gradients. Species codes are defined in Appendix 1.

Species were listed in order of sensitivity to each disturbance gradient, with relative sensitivity defined by location of maximum predicted abundance along the disturbance gradient and more sensitive species having maxima associated with lower disturbance values than less sensitive species (Figure 7, Tables 3 and 4); more steeply sloped monotonic curves were ranked as more sensitive than those with shallower slopes. Six species clearly showed decreasing abundance with increasing in‐lake disturbance, while eight species clearly showed decreasing abundance with increasing watershed disturbance. Among those species with an optimal response to in‐lake disturbance, two species optima were less than the mean in‐lake disturbance (by more than 0.5 units), 14 were near the mean, and two optima were greater than the mean disturbance. Among those species with an optimal response to watershed disturbance, seven species optima were less than the mean watershed disturbance, eight were near the mean, and four optima were greater than the mean disturbance. Five species preferred high levels of in‐lake disturbance, while two species preferred high levels of watershed disturbance. Coregonine species and Yellow Perch were notably classified as most sensitive (or nearly so, more than 0.3 below the mean) to both types of disturbance. More species were sensitive to watershed disturbances (52%) than to in‐lake disturbances (28%). Conversely, defining “malphilic” species as either those with increasing abundances with increasing disturbance or those with optima more than the mean disturbance level, there were seven malphilic species associated with in‐lake disturbance and six malphilic species associated with watershed disturbance, including Smallmouth Bass, which responded positively to both disturbance gradients (Table 4). Several species clearly responded differently to the two types of disturbance. For example, Alewife (*Alosa pseudoharengus*) and White Sucker (*Catostomus commersonii*) preferred high levels of in‐lake disturbance, but were among the most sensitive species to watershed disturbance. Round Goby (*Neogobius melanostomus*) and Channel Catfish (*Ictalurus punctatus*) were among the most sensitive species to in‐lake disturbance, but preferred high levels of watershed disturbance. None of the species were classified into the same response class for both types of disturbance.

### 3.4 Distributions of disturbance

Predictions from all successful models were used to map the spatial distributions of disturbance for each species (Figures S2–S13). In general, the distribution of the most disturbed conditions manifested as patches or extensive bands of habitat along the western and southern shores of Lake Erie and at the eastern end in the Buffalo, NY area, while offshore areas and parts of the western portion of the north shore in the Central ALU (Figure 1) had patches of better habitat (Figure S2). However, the extent and specific locations of degraded, optimal, and hyperoptimal conditions varied by species and differed for each type of disturbance gradient. Several of the species (e.g., Alewife and Smallmouth Bass) that were associated with increasing disturbance had optimal conditions along the western and southern shores and at the eastern end of the lake, but hyperoptimal conditions in other areas.

**FIGURE 8:**
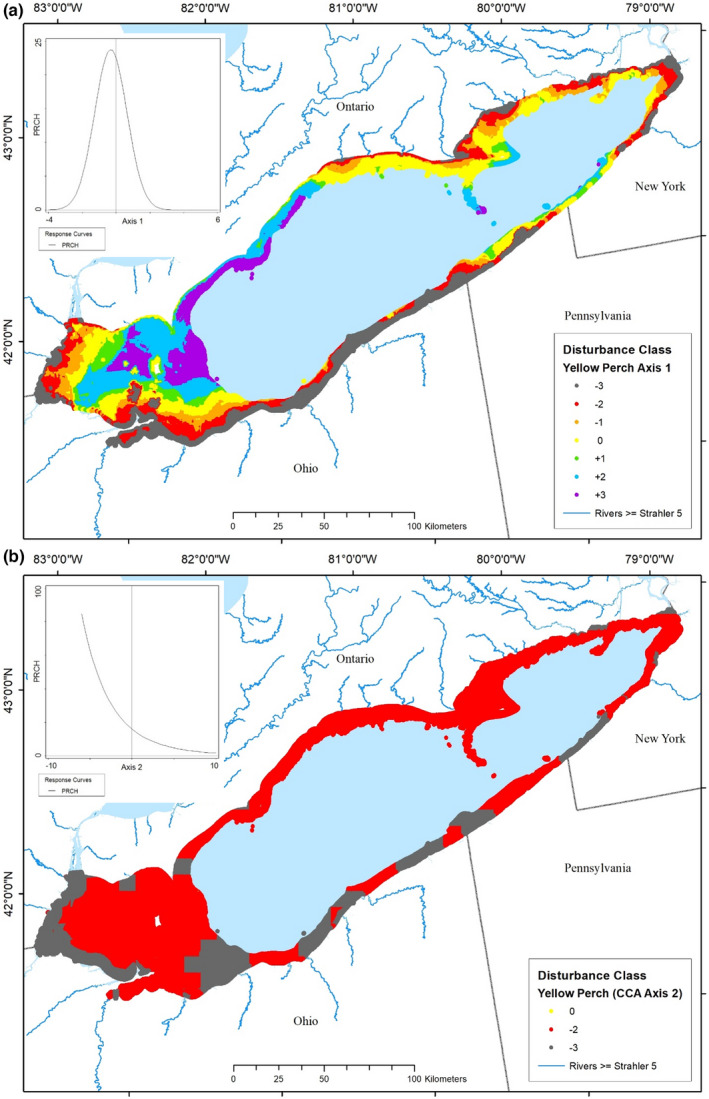
Maps of predicted yellow perch (*Perca flavescens*) perception of disturbance throughout the nearshore zone of Lake Erie based on quadratic models of abundance as a function of (a) in‐lake disturbance and, (b) watershed‐derived disturbance. Disturbance classes range from the most degraded (−3) through optimal (0) to the most hyperoptimal (+3). The palest blue area was outside of the nearshore zone and not considered here. The inset shows the associated quadratic model of yellow perch abundance as a function of the disturbance gradient.

We focus on two common and important fishery species from Lake Erie, Yellow Perch and Smallmouth Bass, and one extirpated species (Cisco) to illustrate differences in the association with disturbance conditions by different fish species in the nearshore zone. The Yellow Perch optimum (PRCH) in the partial CCA ordination space was located in the quadrant where values of each type of disturbance were less than the average (origin) (Figures 5 and 7). The Yellow Perch response to in‐lake disturbance showed a typical unimodal response, increasing in abundance to a maximum at just a little less than average disturbance conditions (−0.306, “optimum” disturbance) and then decreasing with greater disturbance (Table 3, Figure 8a inset). Yellow Perch responded to watershed disturbances with a monotonic curve, decreasing as disturbance increased (Figure 8b inset). Maps of these classified disturbance values (using the −3 through +3 scale) at each spatial location displayed the distribution of disturbance to Yellow Perch habitat conditions throughout the nearshore zone of Lake Erie (Figure 8). The map of in‐lake disturbance showed the highest disturbances (class −3) in bands along the lake shore at the western end, along the south shore from Cedar Point, OH east to Presque Isle, PA, in the Buffalo, NY area, and scattered among other areas of the eastern ALU (14.3% of the nearshore zone; Figure 8a). Less disturbed habitats occurred in bands moving offshore (29.5% of zone) and optimal disturbance conditions occurred in two large bands in the western ALU and around some of the islands, as well as in the eastern half of the lake (19.4% of zone). Several relatively large patches of habitat farthest offshore in the western ALU and western half of the north shore in the central ALU, and scattered in a few other places, were considered to be less disturbed than preferred by Yellow Perch (36.7% of zone). The map of watershed‐derived disturbance indicated that nearly the entire lake was either highly (class −3) or moderately (class −2) disturbed for Yellow Perch, with <1% of the nearshore having suitable conditions (Figure 8b).

**FIGURE 9:**
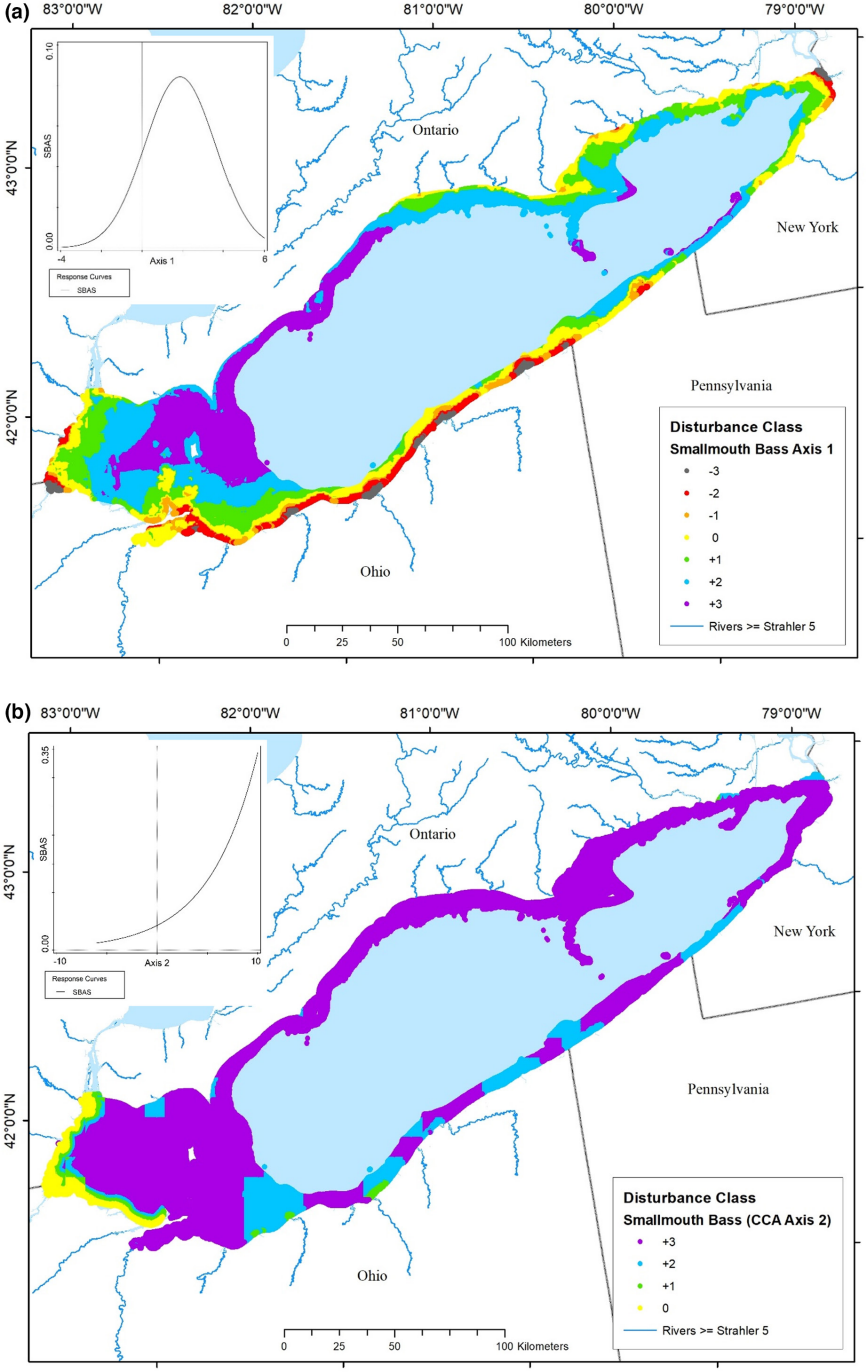
Maps of predicted smallmouth bass (*Micropterus dolomieu*) perception of disturbance throughout the nearshore zone of Lake Erie based on quadratic models of abundance as a function of (a) in‐lake disturbance and, (b) watershed‐derived disturbance. Disturbance classes range from the most degraded (−3) through optimal (0) to the most hyperoptimal (+3). The palest blue area was outside of the nearshore zone and not considered here. The inset shows the associated quadratic model of yellow perch abundance as a function of the disturbance gradient.

The Smallmouth Bass optimum (SBAS) in the partial CCA ordination space was located in the quadrant where values of each type of disturbance were greater than the averages (Figures 5 and 7). The Smallmouth Bass response to in‐lake disturbance showed a unimodal response, increasing in abundance to a maximum at a disturbance value much greater than that of average conditions (1.88, optimum disturbance) and then decreasing with greater disturbance (Table 3, Figure 9a inset). The map of classified in‐lake disturbance values for Smallmouth Bass in the Lake Erie nearshore zone was characterized by the most disturbed conditions (−3) in patches along the south shore in Ohio, including Maumee Bay, and near Buffalo, NY (Figure 9a). Narrow bands of less disturbed conditions occurred in the same areas. Optimal habitat occurred throughout most of Sandusky Bay, OH, and narrow bands offshore of degraded conditions along most of the lake's coast, except for the western half of the north shore. All remaining areas were considerably less disturbed than preferred (hyperoptimal). Smallmouth Bass was one of only two species that responded positively to watershed‐derived disturbances with a monotonic curve increasing as disturbance increased (Figure 9b inset). The map of those “disturbance conditions” showed a band of optimal habitat along the western shore of the lake from the Detroit River to Cedar Point, OH (Figure 9b). All other areas of the lake were less disturbed than preferred by Smallmouth Bass. The preferences of other species for disturbance throughout the nearshore zones of Lake Erie tended to grade between these two examples (Figures S2–S13 species maps).

Interest in Cisco restoration to Lake Erie makes identifying relationships between Cisco abundance and disturbance conditions highly desirable. The Cisco optimum (LKHR) in the partial CCA ordination space was also located in the quadrant with lower than average disturbances (Figure 7). Its quadratic model in response to in‐lake disturbance was monotonically decreasing and its response model to watershed disturbance was unimodal with an optimum at −0.679, indicating that it was sensitive to both types of disturbance. Highly or moderately degraded Cisco habitat conditions were associated with in‐lake disturbance throughout the Lake Erie nearshore zone (Figure S3a). Watershed‐derived disturbance was patchy with optimal conditions for Cisco in narrow bands or patches throughout the nearshore zone; the most extensive optimal conditions were predicted be along the northeastern shore (S10g.). Highly degraded conditions were generally closer to shore, with the most degraded conditions along the western and southern shores, and hyperoptimal conditions generally offshore. A fine‐scale mixture of predicted disturbance conditions occurred in several places, including Sandusky Bay, OH, Presque Isle, PA, and Buffalo, NY.

A cursory examination of general habitat use (pelagic vs benthic) showed a broad mixture of habitat use across the different responses to in‐lake disturbance. However, those species classified as generally pelagic were most sensitive (or within the average disturbance class, but with optima ≤0.0) to watershed disturbance.

### 3.5 Disturbance agreement

**FIGURE 10:**
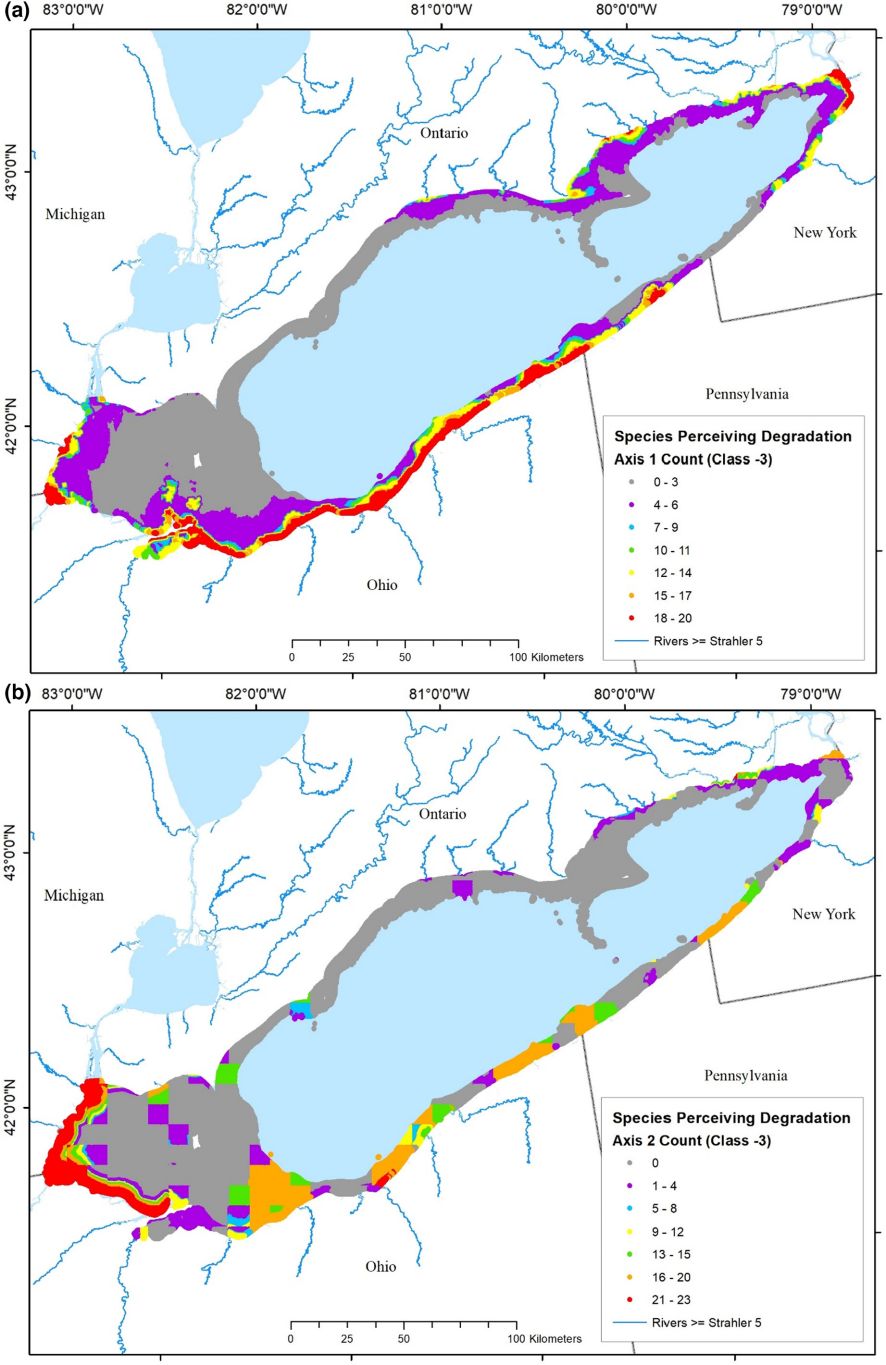
Maps of the distribution of agreement among species that disturbance conditions are perceived as the most degraded (−3) in each 30‐m spatial cell of the Lake Erie nearshore zone due to (a) in‐lake dominated disturbance or (b) watershed dominated disturbance. Ranges of the number of species in agreement compose each color‐coded class, with the greatest agreement shown in red and no agreement shown in gray.

At any given location, predictions of species' abundance‐disturbance relationships will differ. Summing the number of species within a particular disturbance class at a particular location gives a measure of agreement among species about disturbance level. Twenty‐five of the 35 species that occurred >100 times in the dataset were native to Lake Erie, six were established exotics, one was extirpated (Cisco), and three never occurred in Lake Erie (Bloater, Pygmy Whitefish, and Round Whitefish). Discarding species with parabolic models and those absent or extirpated, 25 species models remained for each of the disturbance gradients. Thus, if all species' responses indicated the same level of disturbance at a particular location, the agreement measure would be 25, the maximum number of species that could agree. Counts of species' agreement were determined for each of the seven disturbance classes for each disturbance gradient. For example, if 20 species' model predictions indicated that the most degraded watershed disturbance conditions existed within a spatial cell near Buffalo, NY (orange patches on Figure 10b), and five indicated less degraded conditions, then the agreement measure is 20 and 80% of the species indicate that conditions are highly degraded. Computation of agreement was completed for each of the nearly 10 million 30‐m spatial cells composing the Lake Erie nearshore zone for each disturbance class for each disturbance gradient, and mapped. This created 14 maps of the spatial distribution of fish disturbance throughout Lake Erie's nearshore zone (Figures 9 and 10, and Figures S15–S19).

The greatest degree of agreement among species occurred for the most highly degraded disturbance class (−3). Agreement among species about where in‐lake disturbances were worst, ranged from 0 to 20 (Figure 10a). Between 18 and 20 species indicated that conditions were highly disturbed in Maumee Bay, off the mouth of the Raisin River, MI, and large sections along the south shore of the lake from Cedar Point, OH, to Presque Isle, Pennsylvania, as well as in areas near Buffalo, NY; agreement was greatest closest to the shoreline and decreased moving offshore. Fewer than four species' predictions indicated highly disturbed conditions throughout most of the open waters of the western ALU, western half of the Canadian nearshore zone, and sections along the PA and western NY coasts.

Agreement among species about where watershed‐derived disturbances were worst (−3) ranged from 0 to 23 (Figure 10b). Nearly all species' predictions indicated highly disturbed conditions along the western end of the lake from Cedar Point, OH to the mouth of the Detroit River and in a small area adjacent to Cleveland, OH. More than 17 species indicated that conditions were highly disturbed in large sections along the south shore of the lake, as well as in areas near Buffalo, NY; agreement was greatest closest to the shoreline and decreased rapidly moving offshore from the western end.

**FIGURE 11:**
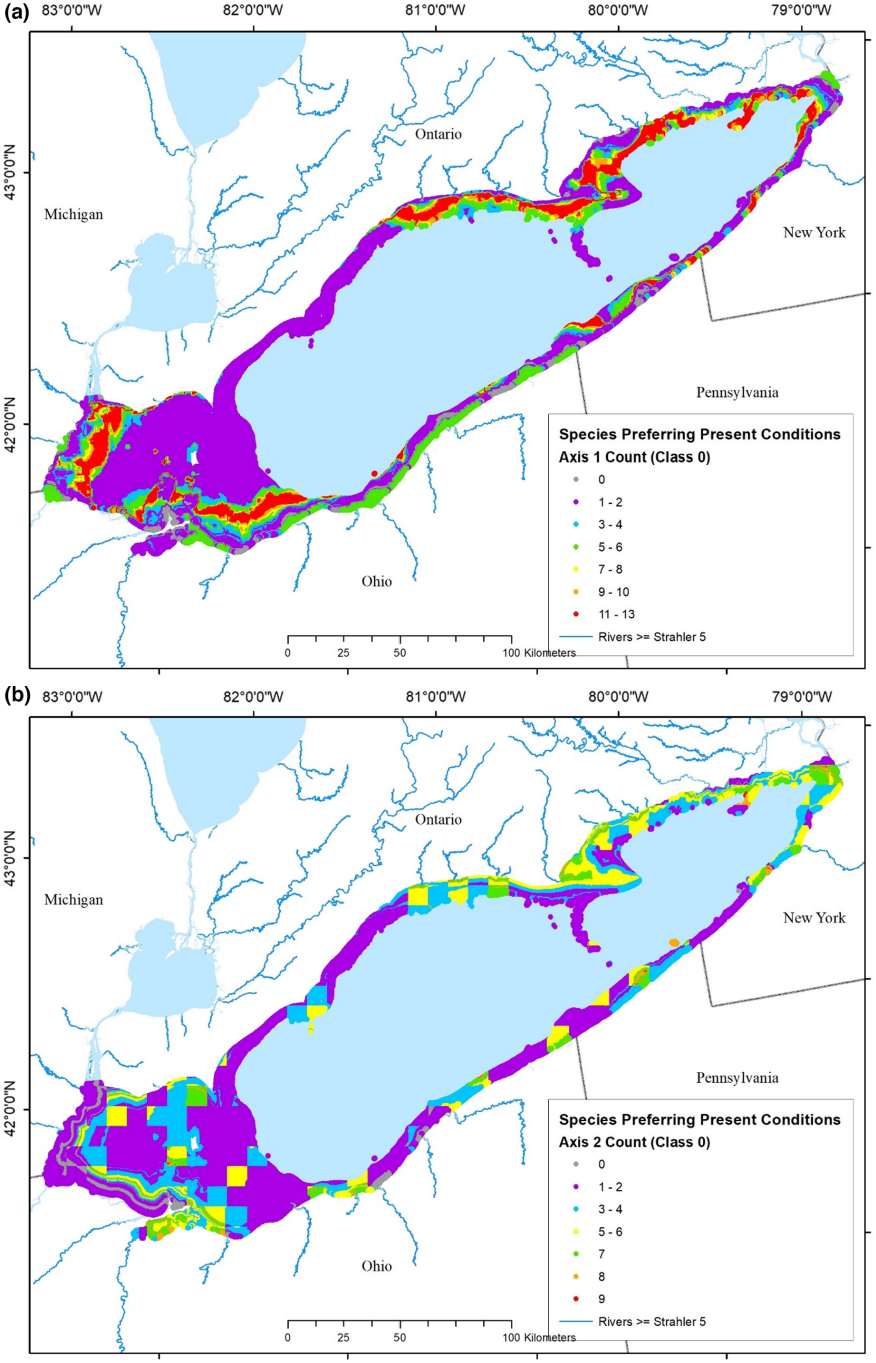
Maps of the distribution of agreement among species that disturbance conditions are perceived as optimal (0) in each 30‐m spatial cell of the Lake Erie nearshore zone due to (a) in‐lake dominated disturbance or (b) watershed dominated disturbance. Ranges of the number of species in agreement compose each color‐coded class, with the greatest agreement shown in red and no agreement shown in gray.

Maps of species agreement counts for lesser degrees of disturbance (classes −2 and −1) showed similar patterns, but with different areal extents for different classes of species agreement (Figures S9 and S15–S16). A general reversal of patterns was observed for agreement among species about where habitat was hyperoptimal (+1, +2, and +3). As many as 19 species' models agreed that conditions offshore in the western ALU were much less disturbed than preferred. Some species' model results identified present conditions in parts of the lake to be appropriate (Figures S5 and S11), but there was less agreement among model results where disturbance levels were optimal (Figure 11). Agreement about optimal in‐lake disturbances ranged from 0 to 13, with >10 species' models agreeing on areas within bands somewhat offshore in the western ALU, along the eastern half of the north shore, and in small areas scattered along the south shore (Figure 11a). There was even less agreement among species' models about where watershed‐derived disturbances were optimal (0), ranging from 0 to 9, with >6 species agreeing on only relatively few, small areas scattered throughout the nearshore zone of the lake (Figure 11b).

## 4 DISCUSSION

That fish populations respond to both natural conditions and anthropogenic disturbance factors is well known. An extensive body of research shows that fish have organs to sense their environments and use that ability to select preferred conditions. Therefore, fish are expected to occur more frequently and in greater relative abundances in environments they perceive as desirable, among those that are available and accessible (Magnuson et al., 1980). The preferred conditions will be in habitat to which they are best adapted, for a given life cycle stage, with possible restrictions due to competition or predation risk, and those best conditions vary by species (Magnuson et al., 1979, 1980; Sharp, 1995; Werner & Hall, 1976). Magnuson et al. (1980) highlight the importance of fishes' perceptions of their environment to their distributions, emphasizing each species' different perception of their world and the human biases that hinder our understanding of those perceptions. They also suggest the use of ordination to standardize the effects of multiple variables on fish habitat selection, as we have done here with CCA. Anthropogenic disturbances are the primary focus of fishery managers, because they are typically both the causes of ecological problems that affect fish populations and the controllable aspects of the environment. To best identify the disturbances that significantly affect fish populations, one must first separate out natural effects. Examination of extensive databases of fish abundances, habitat, and anthropogenic disturbance conditions revealed differences in Great Lakes fish sensitivities and preferences for various levels of human‐perceived disturbances, allowing us to develop predictive models of fish abundances as functions of in‐lake‐ or watershed‐derived disturbance. As a result of differential responses to disturbance and prevalence of multispecies fish assemblages that share habitat, a spatial mosaic of agreement on species‐specific responses to the degree of disturbance existed within the nearshore zones of the Great Lakes.

### 4.1 Regionwide fish communities

The regionwide ordination (full CCA) of nearshore Great Lakes fish assemblages, constrained by natural environmental conditions, confirms that there are relatively distinct fish assemblages in each of the Great Lakes (Riseng et al., 2010). That is not to say that there is not overlap of ranges and wide distributions of many species, but that conditions within each lake are optimal for identifiable subsets of the Great Lakes fish fauna. The five most influential environmental variables (cumulative degree‐days, distance to nearest tributary mouth, mean summer wave height, ice duration, and water depth) were sufficient to explain this general gradient, although more than 20 additional variables contributed to fish distributions. Conditions optimal for Lake Superior (the largest, most northern lake) fishes were associated with the lowest annual heating, and the greatest distance to rivers, wave heights, ice cover, and depth, while the southern and smallest (by area) Lake Ontario, was warm and at the opposite end of the ice and river distance gradients. Lake Erie was moderate for many environmental factors, but is shallowest and smallest in volume, and was set apart along the depth, wave height, and river distance gradients (among others). While White Sucker and Trout‐perch are found throughout the region (their optima were near the ordination origin, indicating preference for average conditions), most other species were more clearly associated with conditions in a particular lake. Fish optima associated with Lake Superior were dominated by cold‐loving species, including the native Coregonine species. Non‐native and stocked species (e.g., Alewife, Chinook Salmon (*Oncorhynchus tshawytscha*), and Brown Trout (*Salmo trutta*)) were prominent in the Lake Ontario assemblage, which also included American Eel (*Anguilla rostrata*), a native species unique within the Great Lakes to Lake Ontario. Conditions in Lake Erie were associated with the most diverse assemblage of species optima (29).

In the Great Lakes, the many different types of disturbances largely come from two sources, within the Great Lakes proper (in‐lake) and adjacent watersheds. Partial CCA filtered out the effects of “natural” habitat conditions and revealed the influences of anthropogenic disturbances derived from integrated variables of in‐lake, watershed, and shoreline perturbations on fish abundances. While the composite variables (CCA constructed) each combine all three original disturbance indices, the dominance of in‐lake (GLEAM) factors explaining most variation (Axis 1) and the greatest influence (longest environmental vector) by watershed (Wehrly index) factors, with a lesser universal influence by shoreline alterations, was clear. The overlap of sample points and species optima, regardless of the lake from which they were collected, suggests that disturbance effects on fish abundances were similar in all lakes (Figure 4). Species optima in the ordination space indicate conditions associated with maximum abundance and peak of a unimodal response to disturbance. Optima were widely scattered along the two primary disturbance gradients, with a dense cluster near the center of the hyperspace, where disturbance conditions are essentially average.

Unimodal responses to environmental conditions are common in nature (Gause, 1930; ter Braak, 1995; Whittaker, 1956) and quadratic models represented the responses of fish abundances to disturbance variables well for the vast majority of species modeled. Because this is an empirical study, based on direct observations, the response models are statistical representations of the correlation between sampled abundance and disturbance gradients, a surrogate for fish perception of conditions. The four different forms of quadratic responses (and the linear responses) highlight differences in perception of disturbance by each species, with some species having optimal conditions within a disturbance gradient (unimodal) and others having optima at (or beyond) one disturbance extreme or the other. The studies producing the original disturbance indices, and others examining effects of stressors, typically take the human perspective of more disturbance is more detrimental to fish. However, our results clearly show that not all fishes are adversely affected by increases in anthropogenic disturbances and other disturbance studies note this in one form or another, e.g., reports of high abundances or species richness in areas of relatively high disturbance (Danz et al., 2007; Kovalenko et al., 2018). Monotonically decreasing fish abundance with increasing disturbance matches the general human perception of the effects of anthropogenic degradation on fish populations. However, both linear and monotonically increasing patterns and the increasing component of unimodal responses clearly indicate preference for more, rather than less, disturbed conditions by some species. Unimodal responses indicate a perception of optimal conditions within a disturbance gradient, with “poorer” conditions at both lower and higher disturbance values. Thus, our results suggest that the human assumption of universal decline of fish abundance with increasing anthropogenic disturbance is too simplistic. Numerous aspects of each species' adaptations could explain these differences and our analysis revealed a weak preference by pelagic species for less disturbed conditions. As Magnuson et al. (1980) recommend, laboratory experimentation could clarify species' responses to these and other disturbance factors (or at least some of them).

Clearly, each species has a different perception of ideal habitat conditions and what constitutes disturbance, and because fish populations occur in multispecies assemblages sharing habitat at particular times, the fish community perception of disturbance will vary from place to place. There were different patchy mosaics of disturbance conditions throughout the nearshore zone of Lake Erie for each species. However, some broad trends occurred. Disturbance was generally greater in shallow waters near the shorelines and was less offshore, which has been previously noted in the Great Lakes (Beeton & Edmondson, 1972). The distribution of highly disturbed conditions in the western ALU and along parts of the southern shore and generally least disturbed conditions along the northern shore (Figures S7, S8, and S3–S14) was reflected in the perceptions of many of the common species modeled here. A minority of species perceived nearly opposite conditions. However, there was wide variability in those perceived distributions and the extent of patches of various quality habitat. Some species were clearly more sensitive to high levels of disturbance and perceived much of the lake to be unsuitable (e.g., Lake Whitefish and Nine‐spine Stickleback (*Pungitius pungitius*)), while others perceived large areas of the lake to be far too pristine to be suitable (e.g., Channel Catfish and Smallmouth Bass). The distributions of those perceived conditions were also different between in‐lake‐ and watershed‐derived disturbances for most species. More species appear to be sensitive to elevated levels of watershed disturbance than those sensitive to in‐lake disturbance. A few species (e.g., Alewife [in‐lake response] and Channel Catfish [watershed response], Figures S7 and S13) preferred high anthropogenic disturbance conditions and their spatial distribution of perceived disturbance was generally opposite to that displayed by most other species. However, even these species showed different perceptions of the effects of disturbance from the two different sources. Yellow Perch and Smallmouth Bass provide illustrations of contrasting responses to disturbances with Yellow Perch being sensitive to both types of disturbance and Smallmouth Bass preferring disturbed conditions.

Combining these species‐specific estimates of perceived disturbance, produced indications of agreement among species about the degree of disturbance at any nearshore location and the extent of those areas. Maps of that agreement provide visual estimates of how many species may benefit (or suffer) from conservation of present conditions or rehabilitation of “degraded” conditions in specific locations or regions. For example, more than a dozen species indicated that conditions were highly degraded along the western and southern coasts of Lake Erie, again generally reflective of the distribution of known anthropogenic disturbance conditions. Concomitantly, few species perceived highly degraded conditions along the northern shore or open water areas of the western ALU. However, fine‐scale details of these distributions show differences between human and fish perceptions of disturbance.

### 4.2 Environmental drivers and land‐lake connections

Anthropogenic changes to the landscape affect physical structure, water quality, hydrology, thermal conditions, and sediment load of receiving waters (Paul & Meyer, 2001; Stanfield & Kilgour, 2006; Wang & Lyons, 2003). These changes have been shown to alter the structure and function of aquatic communities (e.g., Karr & Chu, 1999; Stanfield & Kilgour, 2006; Wang et al., 2003; Wang et al., 2006). The increasing development of the landscape highlights the need to better understand how landscape change influences the ecology of the Great Lakes and the implications for effective conservation and management of fish and fisheries (Wehrly et al., 2013). Approaches to landscape assessment and influences of landscape change on large water bodies like the Great Lakes, assume that the intensity of anthropogenic activities in watersheds and open waters will relate in a definable way to local habitat degradation levels and the biological communities dependent upon those habitats (Allan, 2004; Esselman et al., 2011). Our results support this supposition, but also show that it is not a simple one‐to‐one relationship.

There is extensive research into natural environmental influences and anthropogenic stressors on fish and fish assemblages of the Great Lakes (e.g. Fetzer et al., 2017; Johnson et al., 2016; Kovalenko et al., 2018; McKenna & Castiglione, 2010; Uzarski et al., 2017; Wehrly et al., 2012; Wehrly et al., 2013). Those studies highlight the adverse effects of anthropogenic disturbance on fish, but also the dominant effects of differences in natural conditions. For example, Riseng et al. (2010) conducted ecological assessment of aquatic systems of the Great Lakes within Michigan identifying conditions and sources of ecological impairment that cut across taxonomic groups. Danz et al. (2007) showed that increasing amounts of anthropogenic stress were strongly related to increasing concentrations of water pollutants, and associated with shifts in lentic fish community composition towards non‐native, turbidity‐tolerant species, and to increasing proportions of urban landuse. Wehrly et al. (2012) found that fish species patterns and landscape‐scale environmental data in Michigan lakes were related, and distinct assemblage types associated with climatic conditions. Recently, Kovalenko et al. (2018) used GLAHF, GLEI, and GLCWM data (and programs upon which they are built) to identify key habitat factors in the Great Lakes associated with several coastal fish species, and community metrics. The diversity of responses is notable.

A practical definition of disturbance is change to any aspect of habitat that results in a state that is less suitable for the persistence of a healthy native species community or population of particular management interest (Esselman et al., 2011). While change to a single aspect of habitat may render it unsuitable, multiple interacting environmental stresses are common in aquatic and marine systems, and in combination with effects of natural conditions, complicate management (e.g. Allan et al., 2013; Magnuson et al., 1980; Sherman, 2017; Smith et al., 2015). A landscape (or lakescape) perspective provides an understanding of the effects of broad‐scale and local disturbances on Great Lakes habitat and biotic communities (Wehrly et al., 2013). Previous studies detecting changes in Great Lakes fish assemblages associated with natural and/or disturbed conditions have produced numerous indices of disturbance variables that affect fish. The multiple stressors of the GLEAM and Wehrly indices capture overall disturbance from open lake and watershed sources, respectively (Allan et al., 2013; Wehrly et al., 2013). Disentangling the effects of natural or anthropogenic influences is challenging and ordination has been widely used for that purpose in studies of aquatic habitat stressors (e.g. Brazner et al., 2007; Croft & Chow‐Fraser, 2007; Kovalenko et al., 2018; Seilheimer & Chow‐Fraser, 2007). Partial CCA has been used to separate local vs. watershed influences (Esselman et al., 2011) and anthropogenic stressors from general habitat type and ecoprovence by lake (Danz et al., 2007). In this study, we explicitly accounted for effects of natural influences and were able to focus on the specific effects of anthropogenic disturbances (as represented by the composite index of the GLEAM, Wehrly, and coastal modification indices) on fish abundances, revealing the diversity of species‐specific responses to the same disturbance gradients.

Quantitative expression of anthropogenic disturbance over large geographic areas provides important tools for both research and management (Danz et al., 2007). Among previous Great Lakes studies, the focus has typically been lakewide or regional throughout all the lakes. Several studies have focused on fish assemblages of the coastal zone, which is at the interface between upland and lake processes (Johnson et al., 2016; Kovalenko et al., 2018; Uzarski et al., 2017). However, the nearshore zone is used by 80% of Great Lakes fishes for some or all of their life cycles and anthropogenic disturbance can have major impacts on these nearshore fishes (Lane et al., 1996; Wehrly et al., 2013); cumulative stress can be greatest in nearshore habitats and may threaten ecosystem services (Allan et al., 2013). Our findings show the sensitivity of nearshore zone fishes to anthropogenic disturbances from both watershed and within the Great Lakes proper. While applicable throughout the Great Lakes, we focused on Lake Erie, with its wealth of data, and because several studies have shown it suffers from the greatest disturbance (Allan et al., 2013; Hartman, 1972; Wehrly et al., 2013); changes in Yellow Perch populations in Lake Erie have been attributed to alterations of the prey base associated tributary loadings of nutrients (Hayward & Margraf, 1987; Tyson & Knight, 2001). Our species‐specific response models provide the capability to predict habitat conditions that are most likely to support common species within Lake Erie's nearshore zone and allows modeling of changes in abundances as a function of disturbance remediation. Also, our multiscale representation (30‐m cell to region) of fish responses to disturbance improves on previous broad‐scale tools and provides managers with location specific indications of protection and restoration needs.

### 4.3 Statistical fish perception and data limitations

We speak figuratively here about the “perception” of disturbance by fish. Our observations of apparent perception are based on statistical responses of fish abundances to different levels of anthropogenic disturbance, and the assumption that fish can perceive their environment and select the best available habitats (e.g., Magnuson et al., 1980). Fish are mobile creatures and have multiple sense organ. Numerous experiments have shown that fish can select (or avoid) certain conditions (e.g. Atchison et al., 1987; Magnuson et al., 1979; McKim, 1977; Werner & Hall, 1976). Fish distributions have been linked to temperature and oxygen conditions and these have been used to describe niche dimensions (e.g. Magnuson et al., 1979). Salmon are famous for homing based on olfaction (and other cues) (Bett & Hinch, 2016; Stabell, 1984). Thus, we assume that preferred environments will be the highest quality habitat (perceived by a given species), and that fish will be more abundant in locations with those conditions than in locations with other conditions. We are confident that these statistical representations reflect differential ecological relationships of fish abundances to anthropogenic disturbance, given limitations of sampling gear. The fish were collected with trawls, which may not accurately represent the relative abundances of the various species in Great Lakes nearshore habitats. For example, those species preferring the shallowest waters or areas of rugged bottom may not be as effectively collected as those from smoother bottom areas.

Sampling in many aquatic habitats is difficult and many previous studies of fish community responses to anthropogenic stresses rely upon fish presence and absence data, especially when focused on changes in community metrics, like diversity (i.e., species richness). That approach detects effects of disturbance on assemblage richness and is particularly good for examining losses or persistence of rare species (e.g. Danz et al., 2007; Esselman et al., 2011; Infante & Allan, 2010; Kovalenko et al., 2018). However, where available, fish abundance data have the great advantage of being able to detect optimal and marginal habitats, as well as unsuitable habitats, and can be associated with continuous trends in habitat conditions (McKenna et al., 2013; McKenna & Castiglione, 2014; McKenna & Kocovsky, 2020). The use of standardized abundance data extends that capability to detecting changes in species‐specific populations in response to both natural and anthropogenic disturbances. Studies such as McKenna and Castiglione (2010) used abundance data to identify environmental conditions associated with various fish assemblages in the Western Lake Erie ALU. McKenna and Kocovsky (2020) showed the association of Silver Chub abundance with natural environmental conditions and the effects of selected disturbance factors in Western Lake Erie. We extend previous work by examining the associations of high and low abundances of fishes within Great Lakes assemblages with both “natural” (anthropogenic resistant) conditions and anthropogenic disturbances, and determining species‐specific responses of abundance to disturbances.

The full ordination used data for all species from all of the Great Lakes, but limitations of the disturbance dataset and computational capabilities dictated the present focus on Lake Erie. However, our methods may be applied to all of the Great Lakes and other habitat zones. Using only species with at least 100 occurrences in the data set ensured that statistical responses would be detectable and included the species responsible for most of the biomass and mass and energy flows within the Great Lakes community. However, rare species often constitute a substantial portion of species diversity. Our approach can be expanded to include less common species, but model responses will become weaker as species occurrences decrease. Thus, the present analysis has limited application to biodiversity questions about the relatively common species of Great Lakes fish communities. Also, life stages were lumped together in this analysis and results may differ greatly by life stage. Addressing questions about specific life stages would require more fish data by both location and stage and disturbance data on a seasonal basis.

The fish species examined clearly responded to changes in the multimetric disturbance index values. Most, but not all quadratic models were unimodal, identifying optimal conditions at specific points along the multimetric disturbance gradients; monotonic models identified here might have optima beyond the extent of the observed disturbance gradients or may be monotonic throughout. However, we believe that the disturbance gradients used here represent the range of anthropogenic disturbance conditions in the Great Lakes well (Allan et al., 2013; Hillyer, 1996; Wehrly et al., 2013). Association of specific disturbance factors with each species' response was beyond the scope of this research and dissection of the indices may not be very helpful. Managers are likely to have the best understanding of the important anthropogenic disturbances in their districts and which may feasibly be manipulated to improve conditions.

### 4.4 Potential applications

Recognizing the species‐specific differences in responses to anthropogenic disturbances is critical to effective management of fish habitat. Clearly, while some species are sensitive to different types of anthropogenic disturbances, not all species are adversely affected by those disturbances and a few appear to require (or prefer) disturbed conditions (at least within the range of disturbance conditions observed for this study). This species‐specific response has far reaching implications for managers attempting to “improve” habitat conditions for enhancement or recovery of selected species. Not all species will respond positively to reduced disturbance.

The species‐specific aspects of this research also provide managers with information relevant to species‐specific management plans for fisheries and/or species of concern. Notable among the particularly sensitive species are the native Coregonine species (Loftus & Regier, 1972). Only Lake Whitefish exists in Lake Erie, but Cisco once supported an enormous commercial fishery (Hartman, 1972; Oldenburg et al., 2007). Our results indicate that optimal watershed‐derived disturbance conditions perceived by Cisco existed in narrow bands and patches throughout the nearshore zone. However, Cisco perception of in‐lake disturbance indicated that the entire nearshore zone was degraded.

Estimating the number of species likely to benefit or suffer from changes to habitat conditions can help managers evaluate the costs and benefits of changing habitat conditions in particular areas and to what degree changes must be made (Regier & Loftus, 1972). The perceived degree of disturbance and agreement among species about the degree of disturbance clearly varies within the nearshore zone of Lake Erie and the spatial distribution of that agreement gives an indication of where conditions are best, worst, or moderate and their spatial extents. Areas with the worst conditions might seem to be the best targets for rehabilitation and they are frequently the focus of current restoration efforts (Allan et al., 2013). However, focus on those worst areas might require unreasonable investment of restoration resources and might not benefit many species, or the most desirable fisheries species. In some cases, moderately degraded areas might be better restoration options and indicators of marginal disturbance (in addition to greatly and weakly disturbed conditions) to multiple species may be valuable decision‐support to mangers (Figures S14–S17). Optimal targeting of restoration efforts involves consideration of the range of stressors and their differential effects, the species intended to benefit from a given action and their perception of disturbed conditions, and the number of species that may benefit (or suffer) from the action(s). Fine scale predictions and complete spatial distributions of both in‐lake and watershed anthropogenic disturbance levels perceived by each species provides a powerful tool to managers.

The extent of “natural” conditions suitable for each species may also be limited. The full CCA showed the great difference in optimal conditions for many species and the best combination of those natural factors may not exist in all parts of nearshore zones. Thus, even areas indicated as having optimal disturbance conditions might not have ideal (or even suitable) natural conditions for a given species. Thus, comparisons between the distributions of naturally suitable habitat conditions and perceived disturbance conditions identify overlap between optimal natural and disturbance conditions (McKenna, 2022. *in press*).

#### 4.4.1 Conclusions

By examining the response of each species' relative abundance to increasing values of composite disturbance indices, we extend the work of previous researchers and provide an improved understanding of the perception of anthropogenic disturbances by fish and the diversity of their responses to such disturbances (e.g., Loftus & Regier, 1972). Managers are faced with allocating limited resources (which vary among political districts) to protection or restoration of habitats supporting critical life stages of fishery species, forage species, or other species of concern, as well as ecological issues concerning ecosystem services (Allan et al., 2017). They may ask, how many species will benefit from applying those resources in a particular area or region? Our quadratic models, particularly those with a unimodal response, reveal two important pieces of information, (1) the fact that human understanding of disturbance is at times different from fish population response (Magnuson et al., 1980), and (2) species‐specific information about “ideal” (or best available) versus both degraded and hyperoptimal habitat conditions can be useful for managers to identify the best locations for protection or restoration activities. Overlay of species‐specific maps of fish perceptions of disturbance in any particular location indicates where habitat restoration or protection may benefit the most species, and which species are most strongly affected by multiple disturbances in those areas. The species agreement measures can help provide answers to the above management question at multiple spatial scales. For example, counts within the most degraded disturbance class (−3) showed where numerous species agreed that disturbance conditions were their worst. However, as mentioned above, that estimate may include habitats that are beyond the ability of available resources to rehabilitate and the distribution of slightly or moderately disturbed conditions for many species may provide more practical guidance. Also, the agreement maps provide an indication of how many species may be adversely affected by habitat changes intended to improve conditions for other species in restoration areas, this includes decisions to increase “disturbance” in an area to enhance conditions for some species. The species adversely affected were revealed by the maps, as are nuisance or invasive species that may benefit from the changes.

Our approach may be expanded to other aquatic and marine systems, with appropriate data. More investigation at particular locations and comparison with our results would test model prediction reliability and the broader applicability of this method to natural conditions. Application of our findings will work best in comparison with predictions of the distributions of natural conditions likely to support each species or assemblage (McKenna, 2022 *in press*).

## AUTHOR CONTRIBUTIONS

**James E. McKenna, Jr.:** Conceptualization (lead); data curation (supporting); formal analysis (lead); methodology (lead); project administration (equal); resources (equal); software (lead); visualization (equal); writing – original draft (lead); writing – review and editing (equal). **Catherine Riseng:** Conceptualization (supporting); data curation (lead); formal analysis (supporting); funding acquisition (lead); methodology (supporting); project administration (supporting); resources (equal); software (supporting); visualization (equal); writing – original draft (supporting); writing – review and editing (equal). **Kevin Wehrly:** Conceptualization (supporting); data curation (equal); formal analysis (supporting); funding acquisition (equal); methodology (supporting); project administration (equal); resources (equal); software (supporting); visualization (equal); writing – original draft (supporting); writing – review and editing (equal).

## CONFLICT OF INTEREST

None.

## Supporting information

Supplementary Materials

## Data Availability

Observation data are available from authors of cited references.

## APPENDIX 1 Fish species code names, common names, scientific names, and frequencies of occurrence in the Great Lakes nearshore fish dataset

**Table:**

Species code	Common name	*Scientific name*	Frequency of occurrence
**SMLT**	**Rainbow Smelt**	***Osmerus mordax***	**2672**
**TRPR**	**Trout‐Perch**	***Percopsis omiscomaycus***	**1836**
**PRCH**	**Yellow Perch**	***Perca flavescens***	**1765**
**ALEW**	**Alewife**	***Alosa pseudoharengus***	**1507**
**WHPR**	**White Perch**	***Morone americana***	**1477**
**GOBY**	**Round Goby**	***Neogobius melanostomus***	**1308**
**SPOT**	**Spottail Shiner**	***Notropis hudsonius***	**1110**
**EMRL**	**Emerald Shiner**	***Notropis atherinoides***	**1026**
**SLIM**	**Slimy Sculpin**	***Cottus cognatus***	**913**
**WBAS**	**White Bass**	***Morone chrysops***	**909**
**WALL**	**Walleye**	***Sander vitreus***	**901**
**DRUM**	**Freshwater Drum**	***Aplodinotus grunniens***	**880**
**STK9**	**Nine‐spine Stickleback**	***Pungitius***	**733**
**GIZZ**	**Gizzard Shad**	***Dorosoma cepedianum***	**719**
**LTRT**	**Lake Trout**	***Salvelinus namaycush***	**665**
**WFSH**	**Lake Whitefish**	***Coregonus clupeaformis***	**455**
**JOHN**	**Johnny Darter**	***Etheostoma nigrum***	**379**
**WSUK**	**White Sucker**	***Catostomus commersonii***	**375**
**CCAT**	**Channel Catfish**	***Ictalurus punctatus***	**361**
**LKHR**	**Lake Herring**	***Coregonus artedi***	**361**
**STK3**	**Threespine stickleback**	***Gasterosteus aculeatus***	**308**
**BLOT**	**Bloater**	***Coregonus hoyi***	**283**
**SCHB**	**Silver Chub**	***Macrhybopsis storeriana***	**258**
**SPON**	**Spoonhead Sculpin**	***Cottus ricei***	**256**
**DSCL**	**Deepwater Sculpin**	***Myoxocephalus thompsonii***	**253**
**PUNK**	**Pumpkinseed**	***Lepomis gibbosus***	**182**
**QUIL**	**Quillback**	***Carpiodes cyprinus***	**177**
**LSUK**	**Longnose Sucker**	***Catostomus***	**173**
**BURB**	**Burbot**	***Lota***	**168**
**PWHF**	**Pygmy Whitefish**	***Prosopium coulterii***	**150**
**CARP**	**Common Carp**	***Cyprinus carpio***	**133**
**BLUE**	**Bluegill**	***Lepomis macrochirus***	**126**
**SBAS**	**Smallmouth Bass**	***Micropterus dolomieu***	**108**
**RWHF**	**Round Whitefish**	***Prosopium cylindraceum***	**105**
**MIMC**	**Mimic shiner**	***Notropis volucellus***	**100**
LOGP	Logperch	*Percina caprodes*	99
ROCK	Rock Bass	*Ambloplites rupestris*	96
BBUL	Brown Bullhead	*Ameiurus nebulosus*	83
KING	Chinook Salmon	*Oncorhynchus tshawytscha*	71
MINI	Minnow spp.	*Cyprinidae spp*.	69
AEEL	American Eel	*Anguilla rostrata*	56
SIST	Siscowet (fat lake trout)	*Salvelinus namaycush*	40
BTRT	Brown Trout	*Salmo trutta*	39
SHRH	Shorthead Redhorse	*Moxostoma macrolepidotum*	37
KIYI	Kiyi	*Coregonus kiyi*	35
RUFF	Ruffe	*Gymnocephalus cernua*	18
SJCS	Shortjaw Cisco	*Coregonus zenithicus*	15
LAMP	Sea Lamprey	*Petromyzon marinus*	14
YBUL	Yellow Bullhead	*Ameiurus natalis*	14
CRAP	Black Crappie	*Pomoxis nigromaculatus*	9
GLDF	Goldfish	*Carassius auratus*	7
LBAS	Largemouth Bass	*Micropterus salmoides*	7
STRG	Lake Sturgeon	*Acipenser fulvescens*	7
WCRP	White Crappie	*Pomoxis annularis*	6
LCHB	Lake Chub	*Couesius plumbeus*	5
BROK	Brook Trout	*Salvelinus fontinalis*	4
GRED	Golden Redhorse	*Moxostoma erythrurum*	4
SBUF	Smallmouth Buffalo	*Ictiobus bubalus*	4
COHO	Coho Salmon	*Oncorhynchus kisutch*	3
SPLK	Splake	*BrookTrout x Lake Trout*	3
TGOB	Tubenose Goby	*Proterorhinus marmoratus*	3
CCGF	Common Carp x Goldfish hybrid	*Cyprinus carpio x Carassius auratus*	2
SLMP	Silver Lamprey	*Ichthyomyzon unicuspis*	2
SRDH	Silver Redhorse	*Moxostoma anisurum*	2
STON	Stonecat	*Noturus flavus*	2
ATLS	Atlantic Salmon	*Salmo salar*	1
BUFF	Bigmouth Buffalo	*Ictiobus cyprinellus*	1
BULL	Black Bullhead	*Ameiurus melas*	1
COMM	Common Shiner	*Luxilus cornutus*	1
LDRT	Least Darter	*Etheostoma microperca*	1
MOTT	Mottled Sculpin	*Cottus bairdii*	1
MUSK	Muskellunge	*Esox masquinongy*	1
OSUN	Orangespotted Sunfish	*Lepomis humilis*	1
PIKE	Northern Pike	*Esox lucius*	1
RAIN	Rainbow Trout	*Oncorhynchus mykiss*	1
SAND	Sand Shiner	*Notropis stramineus*	1
SAUG	Sauger	*Sander canadensis*	1
SDRT	Eastern Sand Darter	*Ammocrypta pellucida*	1
SFSH	Spotfin Shiner	*Cyprinella spiloptera*	1
STIK	Brook Stickleback	*Culaea inconstans*	1

*Note*: Boldface species occurred at least 100 times and were used in the evaluation of disturbance effects on Great Lakes nearshore zone fishes.

## APPENDIX 2 Habitat and disturbance variable code names used in ordination figures

**Table:**

Code	Definition
**CDDSST**	**Cumulative degree‐days from mean daily surface water temperature, base 0C.**
**Depth**	**bathymetry depth (m) standardized to 30 m grid in meters.**
DIRWETDELTA	Direction to delta wetland
DIRWETOPEN	Direction to open wetland
DIRWETPROTECT	Direction to protected wetland
**DISTRVRMTH**	**Distance to nearest GLHD pour point, in meters**
**DISTRVRMTH5**	**Distance to nearest river mouth with a Strahler order > = 5, in meters.**
**DISTWETDELTA**	**Distance to delta wetland (m)**
**DISTWETOPEN**	**Distance to open wetland (m)**
**DISTWETPROTECT**	**distance to protected wetland (m)**
**Fetch_**	**GLAHF calculated fetch in meters weighted by direction of wind frequency. Coastal Margin (CM) and Nearshore (NS) zones only.**
GAP_Depth	GLGAP depth (m)
GAP_Fetch	GLGAP fetch distance (m)
GAP_gl_sst_jun	GLGAP mean surface water temperature for June
GAP_RivDens	GLGAP river density
GAP_RIVDIR	GLGAP direction to rivermouth
GAP_RIVDIST	GLGAP distance to rivermouth
GAP_SINUOSITY	GLGAP sinuosity
**GAP_SUBST**	**GLGAP substrate**
GAP_WETDELDIR	GLGAP direction to delta wetland
GAP_WetDeltaDist	GLGAP distance to delta wetland
GAP_WETOPDIR	GLGAP direction to open wetland
GAP_WetOpenDist	GLGAP distance to open wetland
GAP_WETPRODIR	GLGAP direction to protected wetland
GAP_WetProtDist	GLGAP distance to protected wetland
**ICEDUR**	**Ice duration in days, where the ice concentration > = 10%.**
**KM9NEAR03**	**% of shoreline nearshore type “03 (sand/gravel lag over clay) in the KM9 the point falls inside. Shoreline geomorphology layer from the EC/ACOE ~ 1990s dataset.**
**KM9NEAR05**	**% of shoreline nearshore type “05” (bedrock [non‐resistant]) in the KM9 the point falls inside. Shoreline geomorphology layer from the EC/ACOE ~ 1990s dataset.**
KM9NEAR06	% of shoreline nearshore type “06” (unclassified) in the KM9 the point falls inside. Shoreline geomorphology layer from the EC/ACOE ~1990s dataset.
**KM9PROTECT04**	**% of shoreline protected type “4” (no protection: <15% of reach/segment is protected) in the KM9 the point falls inside. Shoreline geomorphology layer from the EC/ACOE ~ 1990s dataset.**
**KM9SHOREGEOMORPH02**	**% of shoreline geomorph type “2” (high [>15 m] bluff with beach) in the KM9 the point falls inside. Shoreline geomorphology layer from the EC/ACOE ~ 1990s dataset.**
**KM9SHOREGEOMORPH13**	**% of shoreline geomorph type “13” (open shoreline wetlands) in the KM9 the point falls inside. Shoreline geomorphology layer from the EC/ACOE ~ 1990s dataset.**
**KM9SHOREGEOMORPH14**	**% of shoreline geomorph type “14” (semi‐protected‐wetlands) in the KM9 the point falls inside. Shoreline geomorphology layer from the EC/ACOE ~ 1990s dataset.**
KM9SHOREGEOMORPH16	% of shoreline geomorph type “16” (unclassified) in the KM9 the point falls inside. Shoreline geomorphology layer from the EC/ACOE ~1990s dataset. Canada shoreline was recoded.
KM9SHOREGEOMORPH99	% of shoreline geomorph type “99” (Unclassified [coded by compiler]) in the KM9 the point falls inside. Shoreline geomorphology layer from the EC/ACOE ~1990s dataset.
**MECHENG**	**GLAHF classification mechanical energy variable (1 = low REI, 2 = moderate REI, 3 = high REI, 4 =)**
REI	Lake bottom relief for a 3 cell by 3 cell area, in meters, derived from bathymetry.
**RELIEF**	**Relief calculated from bathymetry**
**RVRDENS**	**River density for # of closest river mouths from the GLHD.**
**RVRDENS5**	**River density for # of closest river mouths with a Strahler order > =5. Units are “# of points per sq. kilometer”.**
**SINUOSITY**	**GLAHF shoreline sinuosity in values scaled from 1 (straight) to 0 (extremely sinuous). Shoreline used was the GLAHF compiled high resolution shoreline and divided into 1 km segments and then calculated for each line segment.**
SSTSPRCV	Spring surface water temperature CV (between years 1995–2008).
SSTSPRMN	Mean spring vertical water temperature for the 0–20 m water column, in degrees Celsius.
**SUBSTRATE**	**GLAHF compiled substrate from multiple data sources and USACEs 2012 and EC 1990s shoreline material classifications extended to the nearshore zone line. Substrate types are 1 = clay; 2 = mud; 3 = sand; 4 = hard.**
**TRIBINFL3**	**GLAHF classification tributary influence variable, classes are 1 = minimal influence; 2 = moderate influence; and 3 = high influence. Coastal Margin (CM) and Nearshore (NS) zones only.**
**UPWELL**	**Upwelling from surface temperature, an annual index, units are in days.**
**VWT20MSPRCV**	**Spring vertical water temperature CV for the 0–20 m water column.**
VWT20MSPRMN	Mean spring vertical water temperature for the 0–20 m water column, in degrees Celsius.
VWT20MSUMMN	Mean summer vertical water temperature for the 0–20 m water column, in degrees Celsius.
**WVHGHTMNSUM**	**Mean summer wave height in meters.**

*Note*: Boldfaced variables were identified by forward Selection as having significant influence of fish abundances in the nearshore zone of the Great Lakes and were used as co‐variables in the partial CCA of disturbance effects on Great Lakes nearshore fishes.

## APPENDIX 3 Disturbance variables composing the GLEAM, Wehrly, and Coastal Protection indices. See Allan et al. (2013) for complete descriptions of each GLEAM variable, Wehrly et al. (2013) for each Wehrly Index variable, and Hillyer (1996) for Coastal Protection Index variables

**Table:**

Source	Disturbance variable
GLEAM Index	Hypoxia
	Industrial ports and harbors
	Light pollution
	Marinas/boating
	Shipping lanes
	Shoreline extensions
	Shoreline hardening
	Dams (altered flow, nutrient, and sediment regimes)
	Changing water level
	Decreasing ice cover
	Warming water temperature
	Coastal development
	Coastal mines
	Coastal power plants
	Coastal recreational use
	Coastal road density
	Aquaculture
	Commercial fishing
	Native fish stocking
	Non‐native fish stocking
	Recreational fishing – charter
	Ballast risk
	Invasive fish
	Invasive mussels
	Invasive wetland plants
	Sea lampreys
	CSOs
	N loading
	P loading
	Sediment loading
	AOCs
	Metals – biomagnifying
	Metals – non‐biomagnifying
	Organics – biomagnifying
Wehrly Index	Amount of land in agricultural land uses
	Amount of land in urban land uses
	Human population numbers
	Length of roads
	Number of dams
Coastal Protection Index	% of shoreline highly protected type “1” (highly protected: 70%–100% of reach/segment protected)
