# Supplementary material for: Decision support for aquatic restoration based on species‐specific responses to disturbance
Source: Ecol Evol. 2022 Oct 11;12(10):e9313. doi: 10.1002/ece3.9313 (PMC9552897; doi:10.1002/ece3.9313)
Supplement: Supplementary file 1 — Supplementary Materials [file ECE3-12-e9313-s001.docx]

Supplementary Materials for:

**Decision support for aquatic restoration based on species-specific responses to disturbance**

James E. McKenna, Jr.^1^, Catherine Riseng^2^, and Kevin Wehrly^3^

^1^ US Geological Survey, Great Lakes Science Center, Tunison Laboratory of Aquatic Science, 3075 Gracie Road, Cortland, NY, [jemckenna@usgs.gov](mailto:jemckenna@usgs.gov)

^2^ School of Natural Resources and Environment, University of Michigan, Ann Arbor, Michigan 48109, [criseng@umich.edu](mailto:criseng@umich.edu)

^3^ Institute for Fisheries Research, Michigan Department of Natural Resources and University

of Michigan, 212 Museums Annex, 1109 North University Avenue, Ann Arbor, Michigan, 48109, [wehrlyk@michigan.gov](mailto:wehrlyk@michigan.gov)

Any use of trade, product, or firm names is for descriptive purposes only and does not imply endorsement by the U.S. Government.

Table S1. Environmental and disturbance variables used in full CCA.

| **GLAHF observed fish and habitat data with disturbances: Key to variable labels** | |
| --- | --- |
| UniqueID | unique point identifier. TrawlID + 2 letter lake id |
| TrawlID | Trawl identifier (often OP_ID but a combination of IDs for Lake Erie) |
| Agency | Agency data was obtained from |
| LAKE | 2 letter lake identifier, where ER = Erie, HU = Huron, MI = Michigan, ON = Ontario, SU = Superior |
| Year | Year in which trawl occurred |
| OP_DATE | Date on which trawl occurred |
| Port | Port (if reported and applicable) from which vessel left to perform trawl |
| Location | Location where trawl occurred (applies only to trawls in Lake Superior) |
| STATION_DEPTH | Station Depth recorded in RVCAT or other database |
| FishDepth | Fishing Depth recorded in RVCAT or other database |
| BeginDepth | Field recorded depth at beginning of trawl |
| EndDepth | Field recorded depth at end of trawl |
| SUBBASIN | GLAHF subbasin code |
| GLAHFZone | GLAHF spatial framework zone 2 = coastal margin, 3 = nearshore, 4 = offshore |
| AHA | GLGAP unique AHA code |
| UID_9000m | GLAHF unique identifier for each 9000m (9km) grid cell sampling location falls within |
| NNGroupID | Group used for averaging during neural network model development (varies by lake but often AHA, Port_Depth, or Location) |
| LL_location | Where on trawl the reported LL is from (i.e. start, midpoint, or end) |
| FinalLat | Final Latitude location in decimal degrees (after location corrections) |
| FinalLong | Final Longitude location in decimal degrees (after location corrections) |
| Depth | bathymetry depth (m) standardized to 30m grid in meters. |
| Fetch_ | GLAHF calculated fetch in meters weighted by direction of wind frequency. CM and NS zones only. |
| RELIEF | relief calculated from bathymetry |
| MECHENG | GLAHF classification mechanical energy variable (1 = low REI, 2 = moderate REI, 3 = high REI, 4 = |
| REI | Lake bottom relief for a 3 cell by 3 cell area, in meters, derived from bathymetry. |
| DIRWETDELTA | direction to delta wetland |
| DIRWETOPEN | direction to open wetland |
| DIRWETPROTECT | direction to protected wetland |
| DISTWETDELTA | distance to delta wetland (m) |
| DISTWETOPEN | distance to open wetland (m) |
| DISTWETPROTECT | distance to protected wetland (m) |
| DISTRVRMTH5 | Distance to nearest river mouth with a Strahler order >= 5, in meters. |
| DISTRVRMTH | Distance to nearest GLHD pour point, in meters |
| RVRDENS | River density for # of closest river mouths from the GLHD. |
| RVRDENS5 | River density for # of closest river mouths with a Strahler order >= 5. Units are "# of points per sq. kilometer". |
| SINUOSITY | GLAHF shoreline sinuosity in values scaled from 1 (straight) to 0 (extremely sinuous). Shoreline used was the GLAHF compiled high resolution shoreline and divided into 1 km segments and then calculated for each line segment. |
| TRIBINFL3 | GLAHF classification tributary influence variable, classes are 1 = minimal influence; 2 = moderate influence; and 3 = high influence. CM and NS zones only. |
| SUBSTRATE | GLAHF compiled substrate from multiple data sources and USACEs 2012 and EC 1990s shoreline material classifications extended to the nearshore zone line. Substrate types are 1 = clay; 2 = mud; 3 = sand; 4 = hard. |
| CDDSST | Cumulative degree-days from mean daily surface water temperature, base 0C. |
| ICEDUR | Ice duration in days, where the ice concentration >= 10%. |
| SSTSPRCV | Spring surface water temperature CV (between years 1995-2008). |
| SSTSPRMN | Mean spring vertical water temperature for the 0-20m water column, in degrees Celsius. |
| UPWELL | Upwelling from surface temperature, an annual index, units are in days. |
| VWT20MSPRCV | Spring vertical water temperature CV for the 0-20m water column. |
| VWT20MSPRMN | Mean spring vertical water temperature for the 0-20m water column, in degrees Celsius. |
| VWT20MSUMMN | Mean summer vertical water temperature for the 0-20m water column, in degrees Celsius. |
| KM9NEAR03 | % of shoreline nearshore type "03 (sand/gravel lag over clay) in the KM9 the point falls inside. Shoreline geomorphology layer from the EC/ACOE ~1990s dataset. |
| KM9NEAR05 | % of shoreline nearshore type "05" (bedrock [non-resistant]) in the KM9 the point falls inside. Shoreline geomorphology layer from the EC/ACOE ~1990s dataset. |
| KM9NEAR06 | % of shoreline nearshore type "06" (unclassified) in the KM9 the point falls inside. Shoreline geomorphology layer from the EC/ACOE ~1990s dataset. |
| KM9PROTECT01 | % of shoreline protected type "1" (highly protected: 70-100% of reach/segment protected) in the KM9 the point falls inside. Shoreline geomorphology layer from the EC/ACOE ~1990s dataset. |
| KM9PROTECT04 | % of shoreline protected type "4" (no protection: <15% of reach/segment is protected) in the KM9 the point falls inside. Shoreline geomorphology layer from the EC/ACOE ~1990s dataset. |
| KM9SHOREGEOMORPH02 | % of shoreline geomorph type "2" (high [>15m] bluff with beach) in the KM9 the point falls inside. Shoreline geomorphology layer from the EC/ACOE ~1990s dataset. |
| KM9SHOREGEOMORPH13 | % of shoreline geomorph type "13" (open shoreline wetlands) in the KM9 the point falls inside. Shoreline geomorphology layer from the EC/ACOE ~1990s dataset. |
| KM9SHOREGEOMORPH14 | % of shoreline geomorph type "14" (semi-protected-wetlands) in the KM9 the point falls inside. Shoreline geomorphology layer from the EC/ACOE ~1990s dataset. |
| KM9SHOREGEOMORPH16 | % of shoreline geomorph type "16" (unclassified) in the KM9 the point falls inside. Shoreline geomorphology layer from the EC/ACOE ~1990s dataset. Canada shoreline was recoded. |
| KM9SHOREGEOMORPH99 | % of shoreline geomorph type "99" (Unclassified [coded by compiler]) in the KM9 the point falls inside. Shoreline geomorphology layer from the EC/ACOE ~1990s dataset. |
| WVHGHTMNSUM | Mean summer wave height in meters. |
| GAP_Depth | GLGAP depth (m) |
| GAP_Fetch | GLGAP fetch distance (m) |
| GAP_WETDELDIR | GLGAP direction to delta wetland |
| GAP_WETOPDIR | GLGAP direction to open wetland |
| GAP_WETPRODIR | GLGAP direction to protected wetland |
| GAP_WetOpenDist | GLGAP distance to open wetland |
| GAP_WetProtDist | GLGAP distance to protected wetland |
| GAP_WetDeltaDist | GLGAP distance to delta wetland |
| GAP_RIVDIR | GLGAP direction to rivermouth |
| GAP_RIVDIST | GLGAP distance to rivermouth |
| GAP_RivDens | GLGAP river density |
| GAP_SINUOSITY | GLGAP sinuosity |
| GAP_SUBST | GLGAP substrate |
| GAP_gl_sst_jun | GLGAP mean surface water temperature for June |
| gleamCoastalRI_5Brks | GLEAM Coastal risk index set as 5 breaks (1 = very low, 5 = very high), scaled by lake, used for nearshore assessment |
| gleamCoastalRI_continuous | GLEAM Coastal risk index continuous values from GLEAM project staff |
| wehrlyRI_5logBrks | Wehrly et al. (2012) watershed risk index values set as 5 breaks (1 = very low, 5 = very high), scaled by lake, log breaks, used for nearshore assessment |
| wehrlyRI_continuous | Wehrly et al. (2012) watershed risk index values attributed (and decayed) into coastal margin and nearshore zones as continuous values |

Fig. S1. Quadratic model predictions for species with optima within the watershed disturbance gradient. Species abundances scaled to the primary ordinate (0 – 30): Round Goby (GOBY), Nine-spine Stickleback (STK9), White Bass (WBAS), Lake Whitefish (WFSH), Johnny Darter (JOHN), Slimy Sculpin (SLIM), Longnose Sucker (LSUK), Silver Chub (SCHB), Pygmy Whitefish (PWHF), Mimic Shiner (MIMC) , Cisco (LKHR), Round Whitefish (RWHF), Deepwater Sculpin (DSCL), Lake Trout (LTRT), and Spoonhead Sculpin (SPON); Species abundances scaled to the secondary ordinate (0 – 225): Rainbow Smelt (SMLT), Emerald Shiner (EMRL), Trout-perch (TRPR), and Three-spine Stickleback (STK3). Species codes used in the legend are defined in Appendix I.

Fig. S2. Spatial distributions of disturbance indices within Lake Erie based on a.) GLEAM composite index, b.) Wehrly composite index, and c.) Coastal Modification index

Fig. S3. Map of in-lake dominated disturbance (represented as predicted fish abundances) throughout the nearshore zone of Lake Erie perceived as monotonically decreasing by a.) Cisco (*Coregonus artedi*), b.) Round Whitefish (*Prosopium cylindraceum*), c.) Lake Whitefish (*Coregonus* *clupeaformis*), d.) Nine-spine Stickleback (*Pungitius* *pungitius*), e.) Pygmy Whitefish (*Prosopium* *coulterii*), and f.) Round Goby (*Neogobius* *melanostomus*).

Fig. S4. Map of in-lake dominated disturbance (represented as predicted fish abundances) throughout the nearshore zone of Lake Erie perceived as being optimal at a value less than the mean disturbance value by a.) Johnny Darter (*Etheostoma* *nigrum*), and b.) Channel Catfish (*Ictalurus* *punctatus*).

Fig. S5. Map of in-lake dominated disturbance (represented as predicted fish abundances) throughout the nearshore zone of Lake Erie perceived as being optimal at a value near the mean disturbance value by a.) Yellow Perch (*Perca* *flavescens*), and b.) Bloater (*Coregonus* *hoyi*), c.) Spottail Shiner (*Notropis* *hudsonius*), d.) Walleye (*Sander* *vitreus*), e.) Quilback (*Carpiodes* *cyprinus*), f.) White Bass (*Morone* *chrysops*), g.) Freshwater Drum (*Aplodinotus* *grunniens*), h.) Silver Chub (*Macrhybopsis* *storeriana*), i.) White Perch (*Morone* *americana*), j.) Trout-perch (*Percopsis* *omiscomaycus*), k.) Gizzard Shad (*Dorosoma* *cepedianum*), l.) Bluegill (*Lepomis* *macrochirus*), m.) Pumpkinseed (*Lepomis* *gibbosus*), and n.) Emerald Shiner (*Notropis* *atherinoides*).

Fig. S6. Map of in-lake dominated disturbance (represented as predicted fish abundances) throughout the nearshore zone of Lake Erie perceived as being optimal at a value greater than the mean disturbance value by a.) Mimic Shiner (*Notropis* *volucellus*), and b.) Smallmouth Bass (*Micropterus* *dolomieu*).

Fig. S7. Map of in-lake dominated disturbance (represented as predicted fish abundances) throughout the nearshore zone of Lake Erie perceived as monotonically increasing by a.) Alewife (*Alosa* *pseudoharengus*), b.) Longnose Sucker (*Catostomus* *catostomus*), c.) Rainbow Smelt (*Osmerus* *mordax*), and d.) Spoonhead Sculpin (*Cottus* *ricei*).

Fig. S8. Map of predicted species abundance to in-lake dominated disturbance throughout the nearshore zone of Lake Erie generated from a concave up parabolic response for a.) Burbot (Lota lota), b.) Deepwater Sculpin (*Myoxocephalus* *thompsonii*), c.) Lake Trout (*Salvelinus* *namaycush*), d.) Slimy Sculpin (*Cottus* *cognatus*), and e.) Three-spine Stickleback (*Gasterosteus* *aculeatus*).

Fig. S9. Map of watershed dominated disturbance (represented as predicted fish abundances) throughout the nearshore zone of Lake Erie perceived as monotonically decreasing by a.) Bloater (*Coregonus hoyi*), b.) Freshwater Drum (*Aplodinotus* *grunniens*), c.) White Perch (*Morone* *americana*), d.) Pumpkinseed (*Lepomis* *gibbosus*), e.) Yellow Perch (*Perca* *flavescens*), f.) Quillback (*Carpiodes* *cyprinus*), g.) White Sucker (*Catostomus* *commersonii*), and h.) Alewife (*Alosa* *pseudoharengus*).

Fig. S10. Map of watershed dominated disturbance (represented as predicted fish abundances) throughout the nearshore zone of Lake Erie perceived as being optimal at a value less than the mean disturbance value by a.) Lakes Whitefish (*Coregonus* *clupeaformis*), b.) Silver Chub (*Macrhybopsis* *storeriana*), c.) Emerald Shiner (*Notropis* *atherinoides*), d.) Pygmy Whitefish (*Prosopium* *coulterii*), e.) White Bass (*Morone* *chrysops*), f.) Mimic Shiner (*Notropis* *volucellus*), and g.) Cisco (*Coregonus* *artedi*).

Fig. S11. Map of watershed dominated disturbance (represented as predicted fish abundances) throughout the nearshore zone of Lake Erie perceived as being optimal at a value near the mean disturbance value by a.) Rainbow Smelt (*Osmerus* *mordax*), and b.) Round Whitefish (*Prosopium* *cylindraceum*), c.) Three-spine Stickleback (*Gasterosteus* *aculeatus*), d.) Deepwater Sculpin (*Myoxocephalus* *thompsonii*), e.) Nine-spine Stickleback (*Pungitius* *pungitius*), f.) Lake Trout (*Salvelinus* *namaycush*), g.) Johnny Darter (*Etheostoma* *nigrum*), and h.) Slimy Sculpin (*Cottus* *cognatus*).

Fig. S12. Map of watershed dominated disturbance (represented as predicted fish abundances) throughout the nearshore zone of Lake Erie perceived as being optimal at a value greater than the mean disturbance value by a.) Trout-perch (*Percopsis* *omiscomaycus*), b.) Spoonhead Sculpin (*Cottus* *ricei*), c.) Longnose Sucker (*Catostomus* *catostomus*), and d.) Round Goby (*Neogobius* *melanostomus*).

Fig. S13. Map of watershed dominated disturbance (represented as predicted fish abundances) throughout the nearshore zone of Lake Erie perceived as monotonically increasing by a.) Channel Catfish (*Ictalurus* *punctatus*), and b.) Smallmouth Bass (*Micropterus* *dolomieu*).

Fig. S14. Map of predicted species abundance to watershed dominated disturbance throughout the nearshore zone of Lake Erie generated from a concave up parabolic response for a.) Common Carp (*Cyprinus* carpio), and b.) Gizzard Shad (*Dorosoma* *cepedianum*).

Fig. S15. Maps of agreement that disturbance level is perceived as moderately degraded (-2) due to a) in-lake disturbance or b) watershed disturbance.

Fig. S16. Maps of agreement that disturbance level is perceived as somewhat degraded (-1) due to a) in-lake disturbance or b) watershed disturbance.

Fig. S17. Maps of agreement that disturbance level is perceived as somewhat hyperoptimal (+1) due to a) in-lake disturbance or b) watershed disturbance.

Fig. S18. Maps of agreement that disturbance level is perceived as moderately hyperoptimal (+2) due to a) in-lake disturbance or b) watershed disturbance.

Fig. S19. Maps of agreement that disturbance level is perceived as highly hyperoptimal (+3) due to a) in-lake disturbance or b) watershed disturbance.

Supplementary Figures


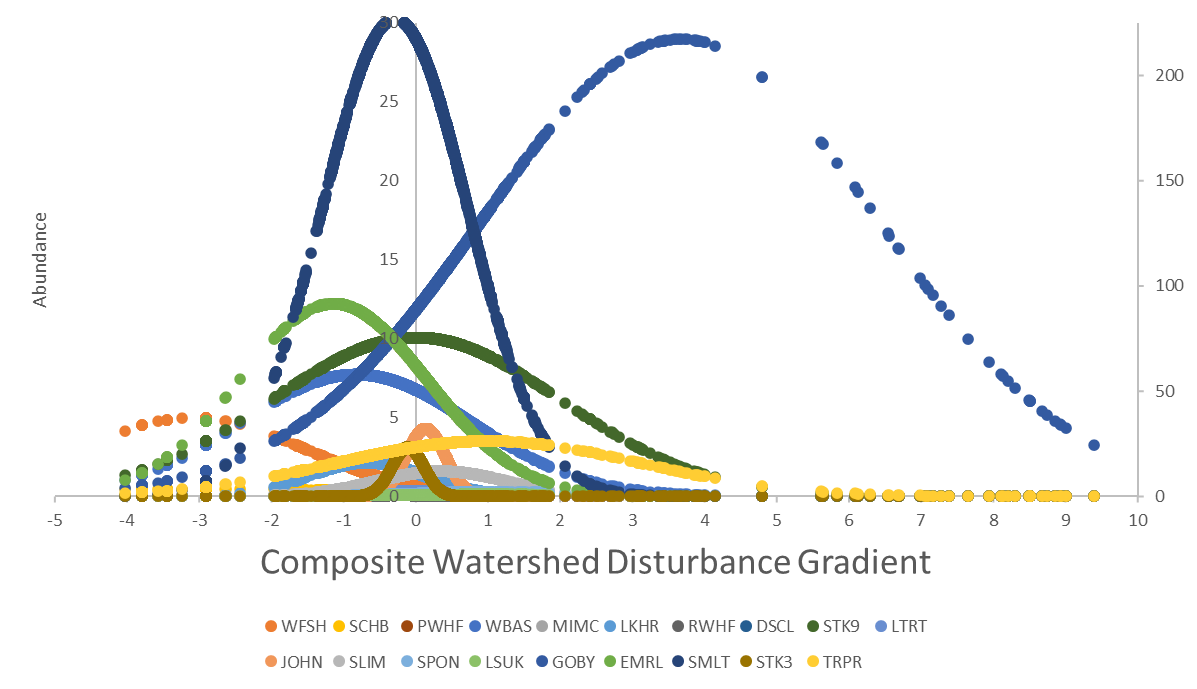
Fig. S1.

Fig. S2.


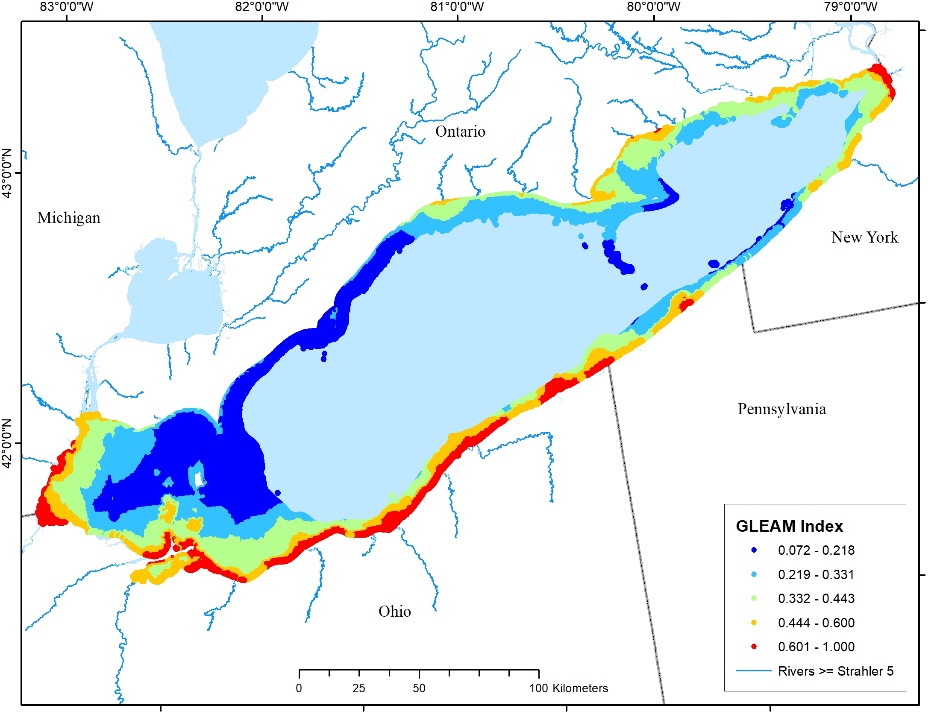
a.)

Buffalo


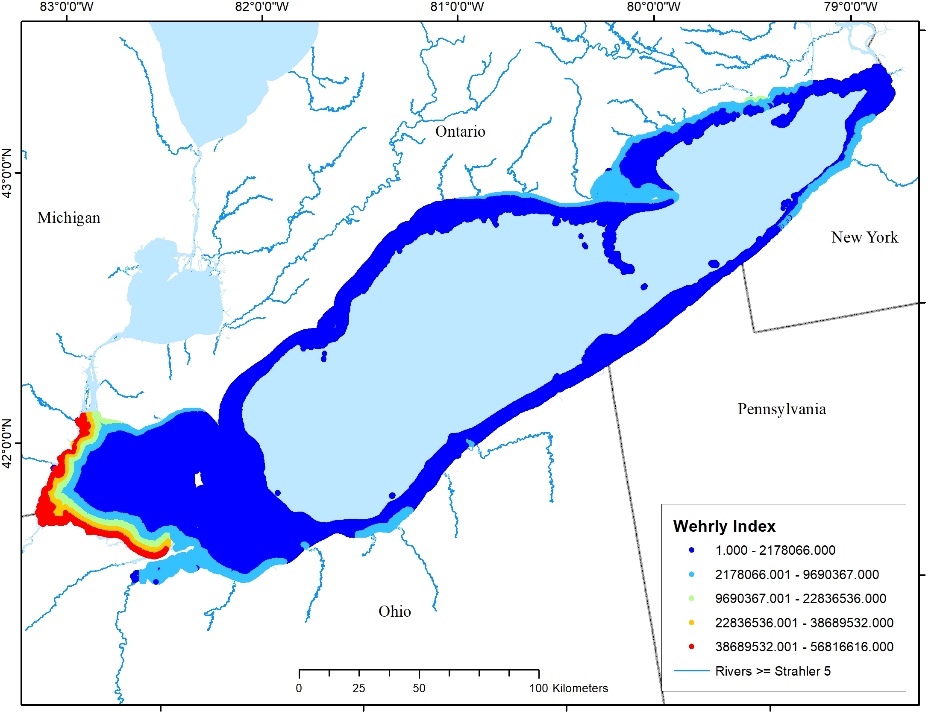


b.)


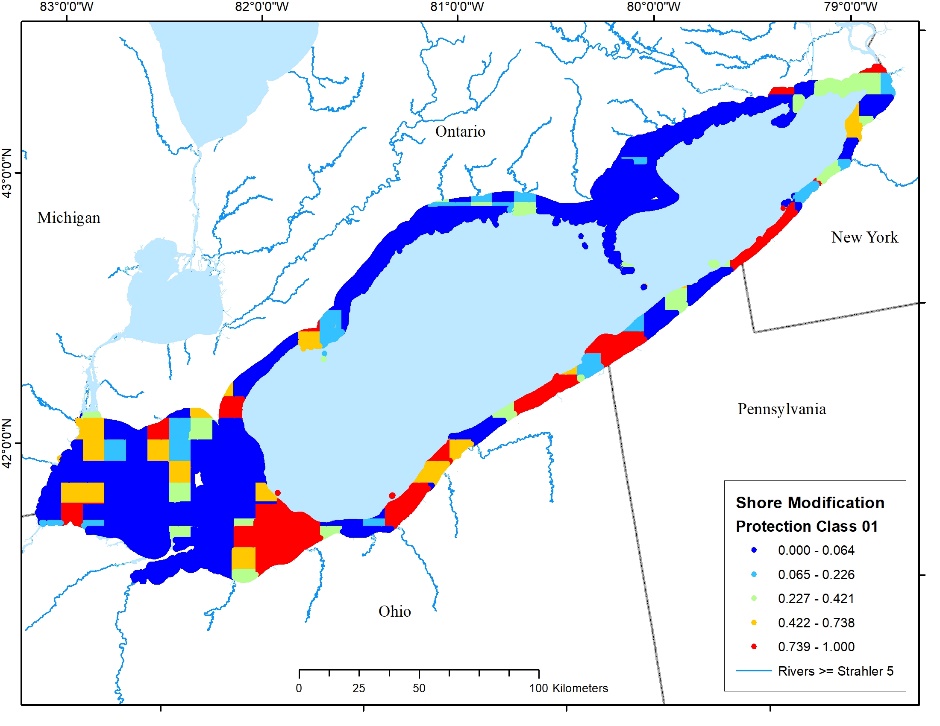


c.)

Fig. S3.a


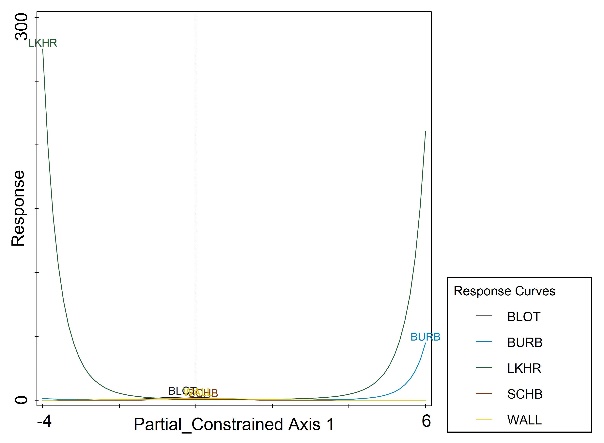

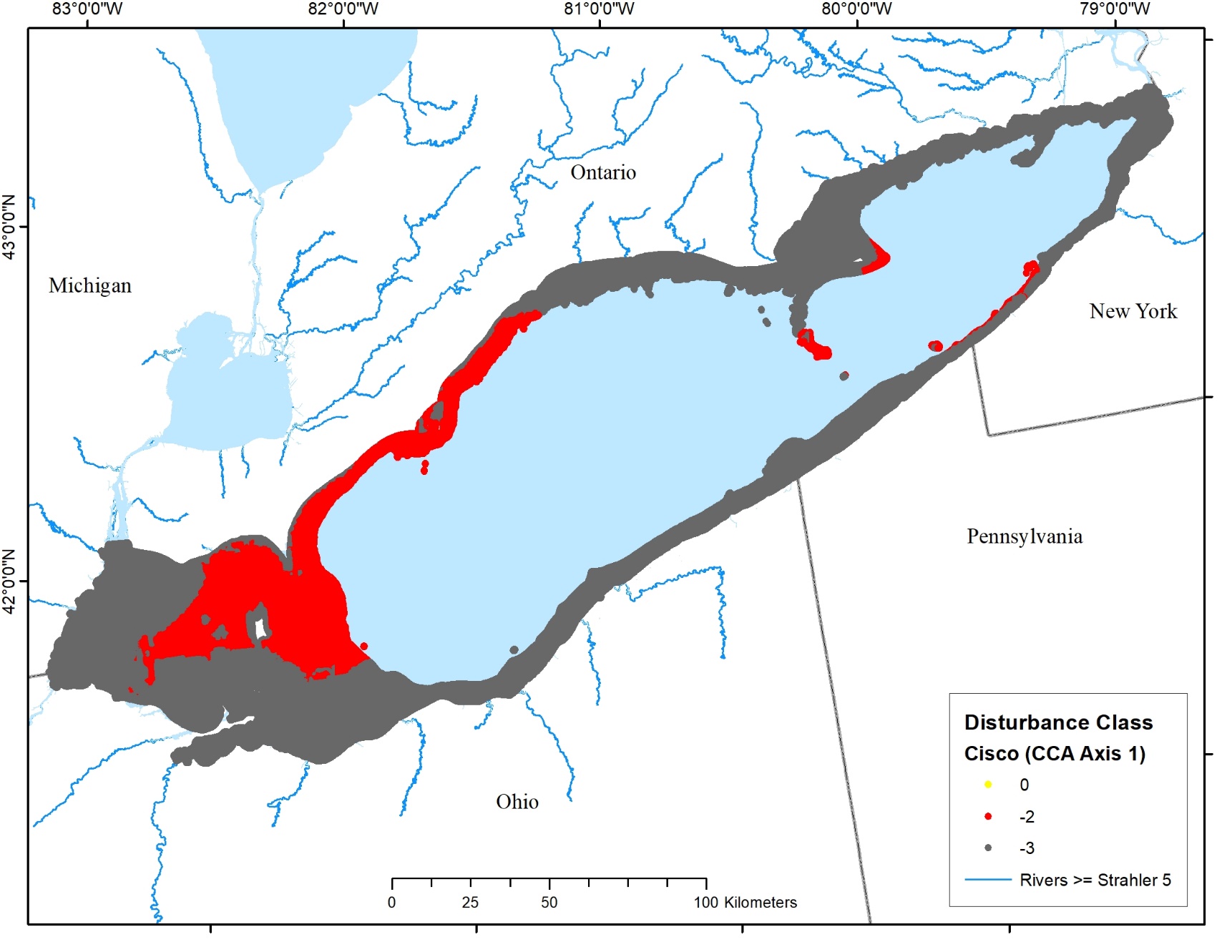


Fig. 3. b.


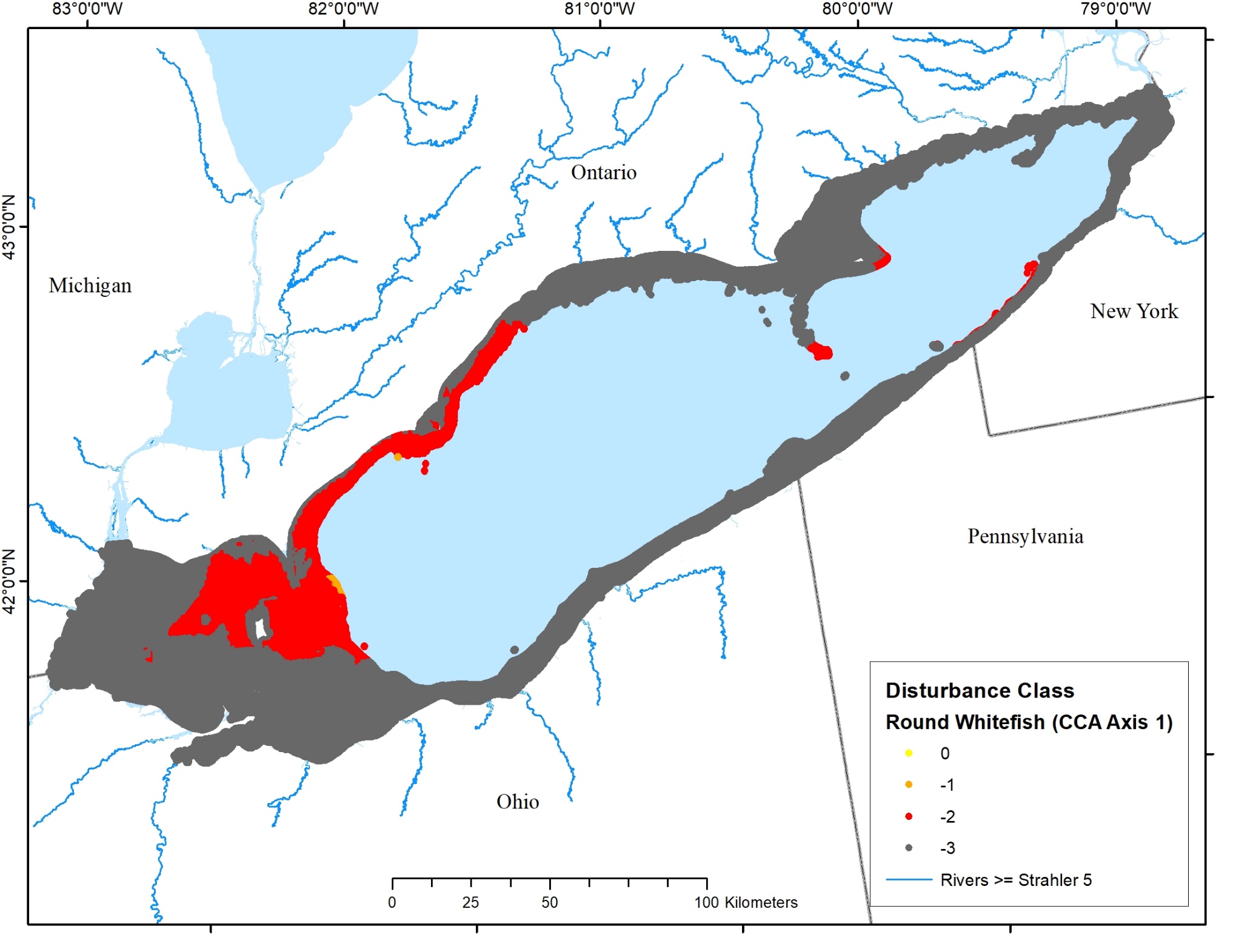


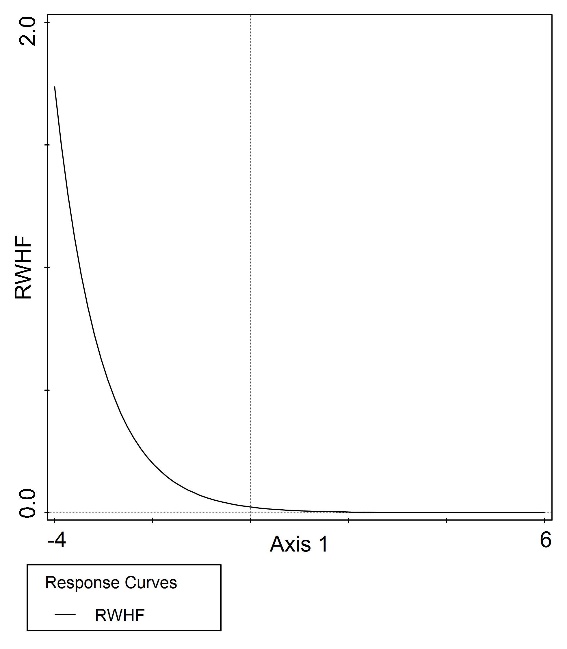


Fig. S3. c.


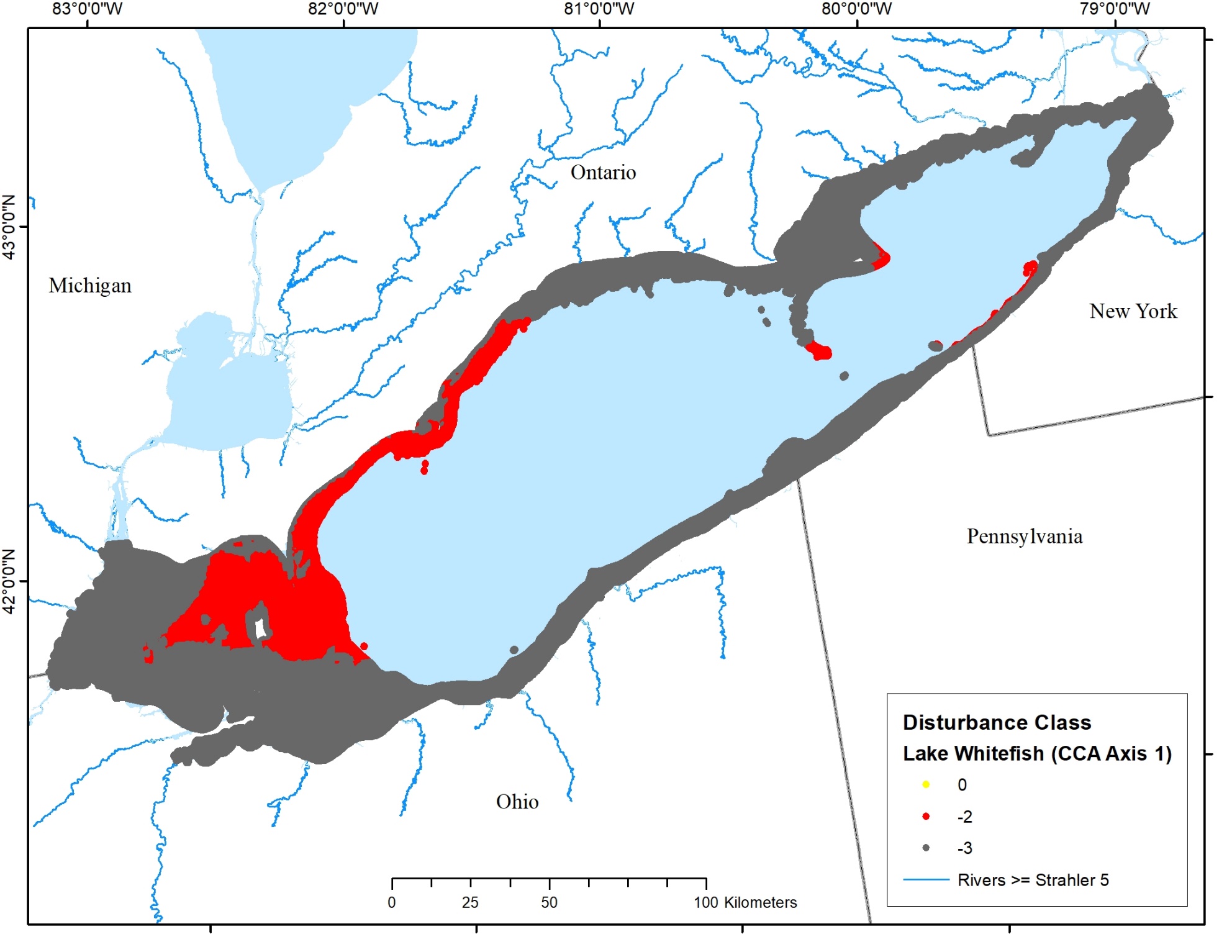


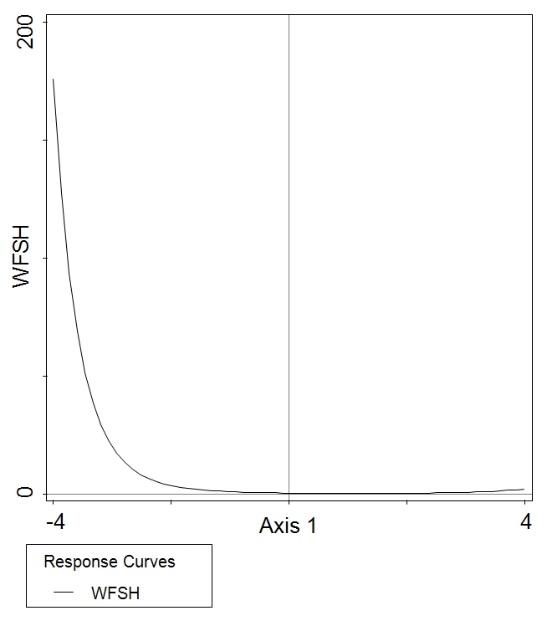


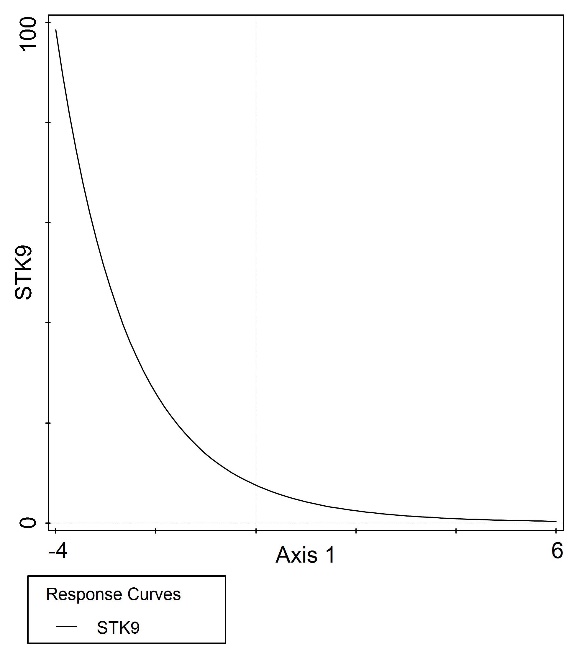

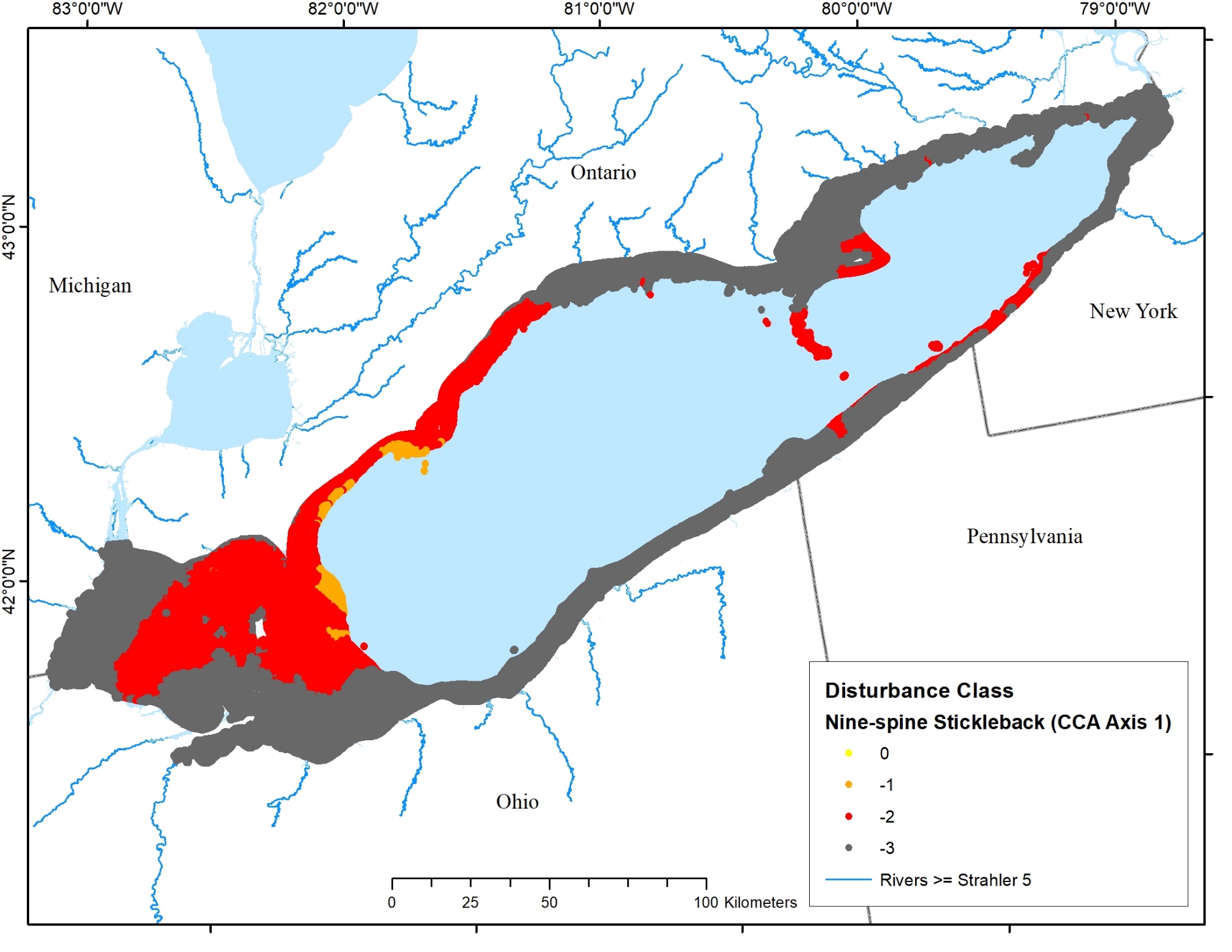
Fig. 3. d.


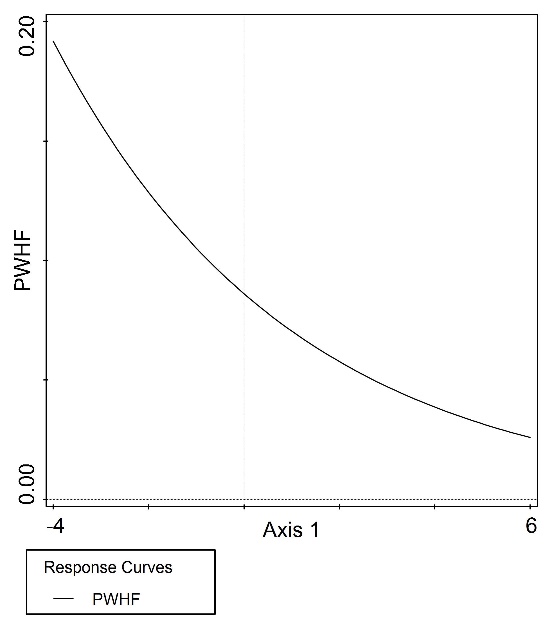

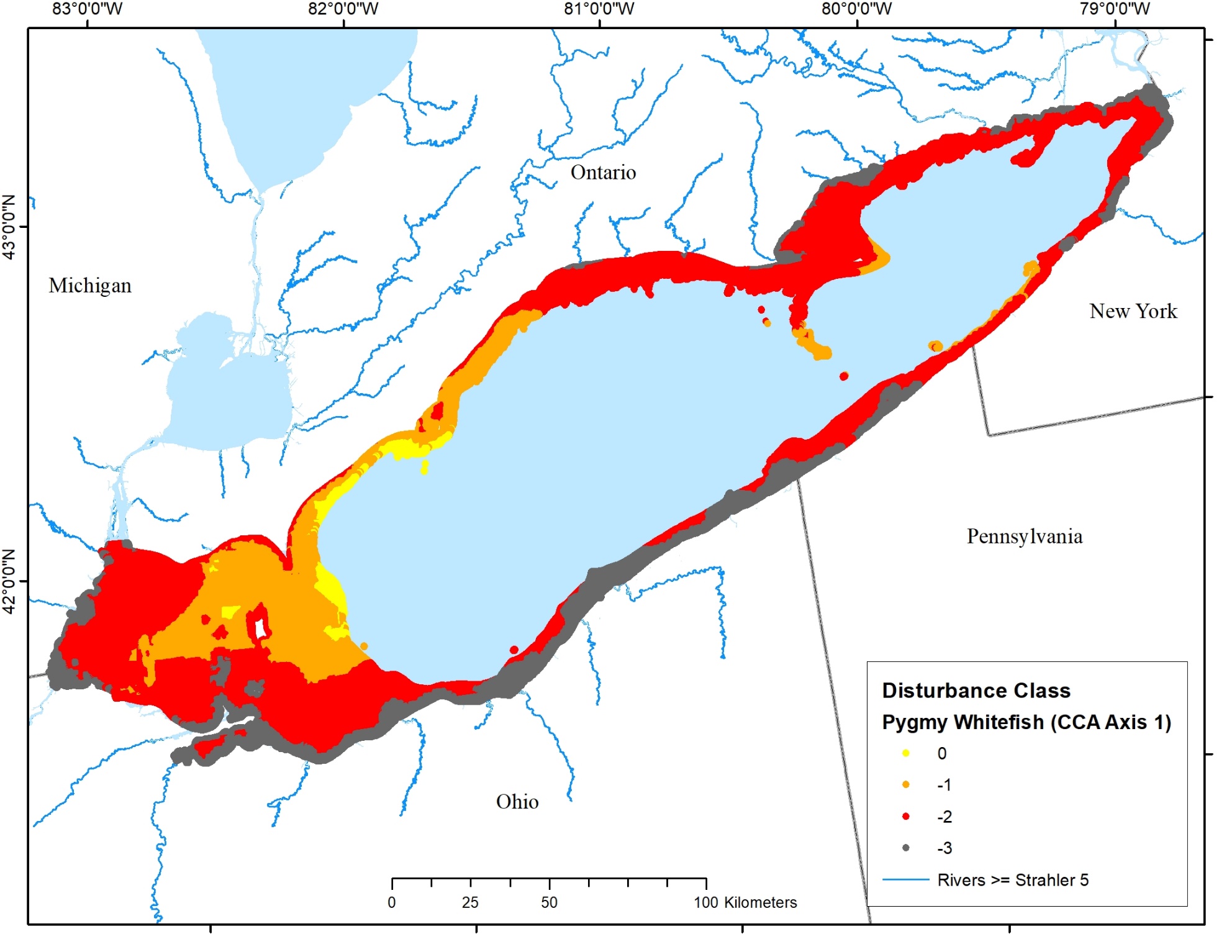
Fig. 3. e.

Fig. 3. f.


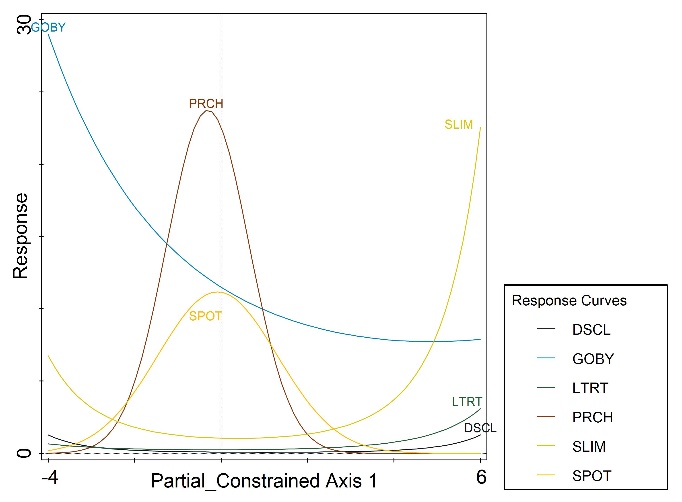

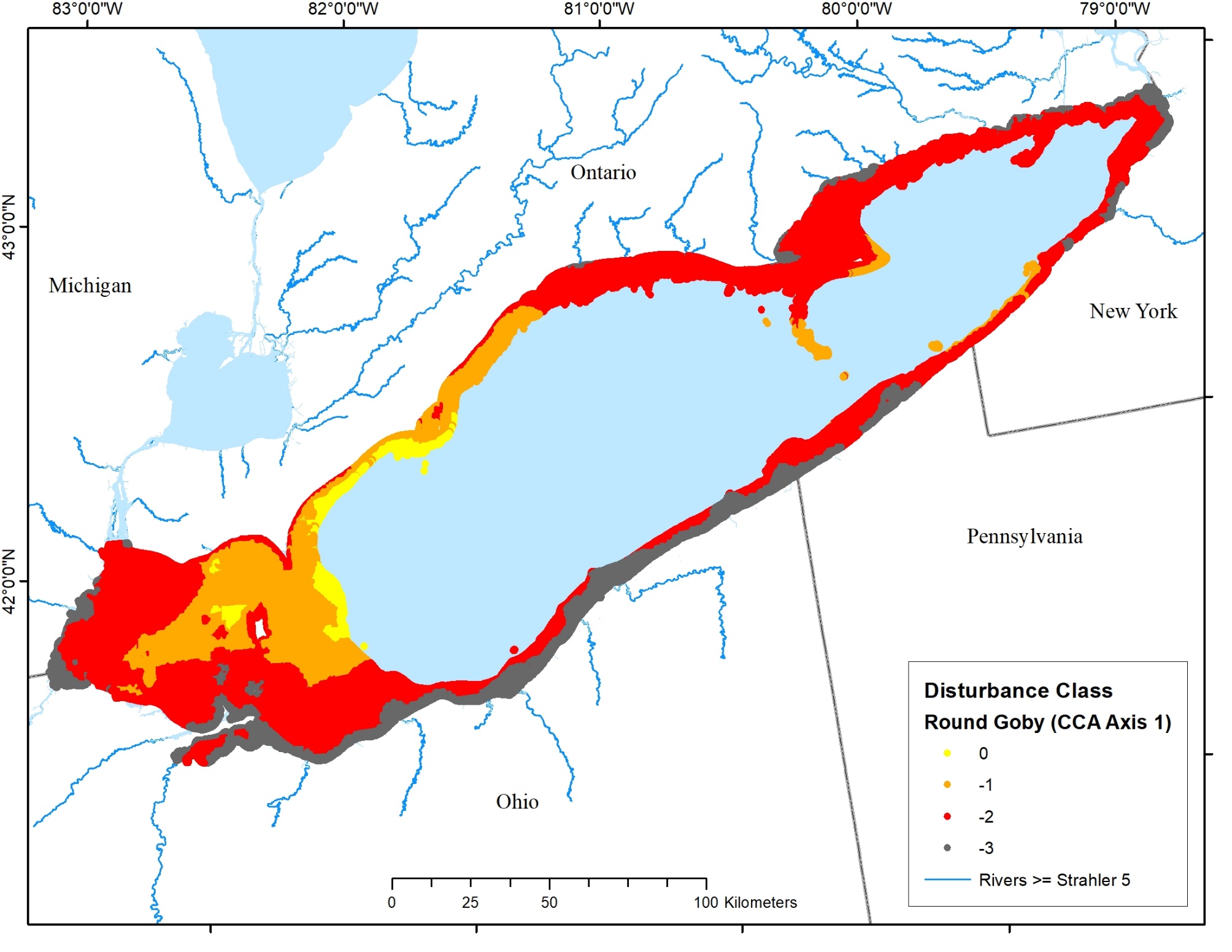


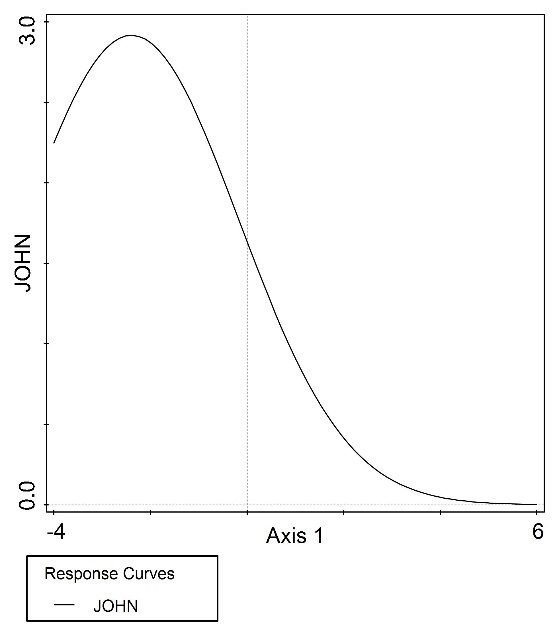

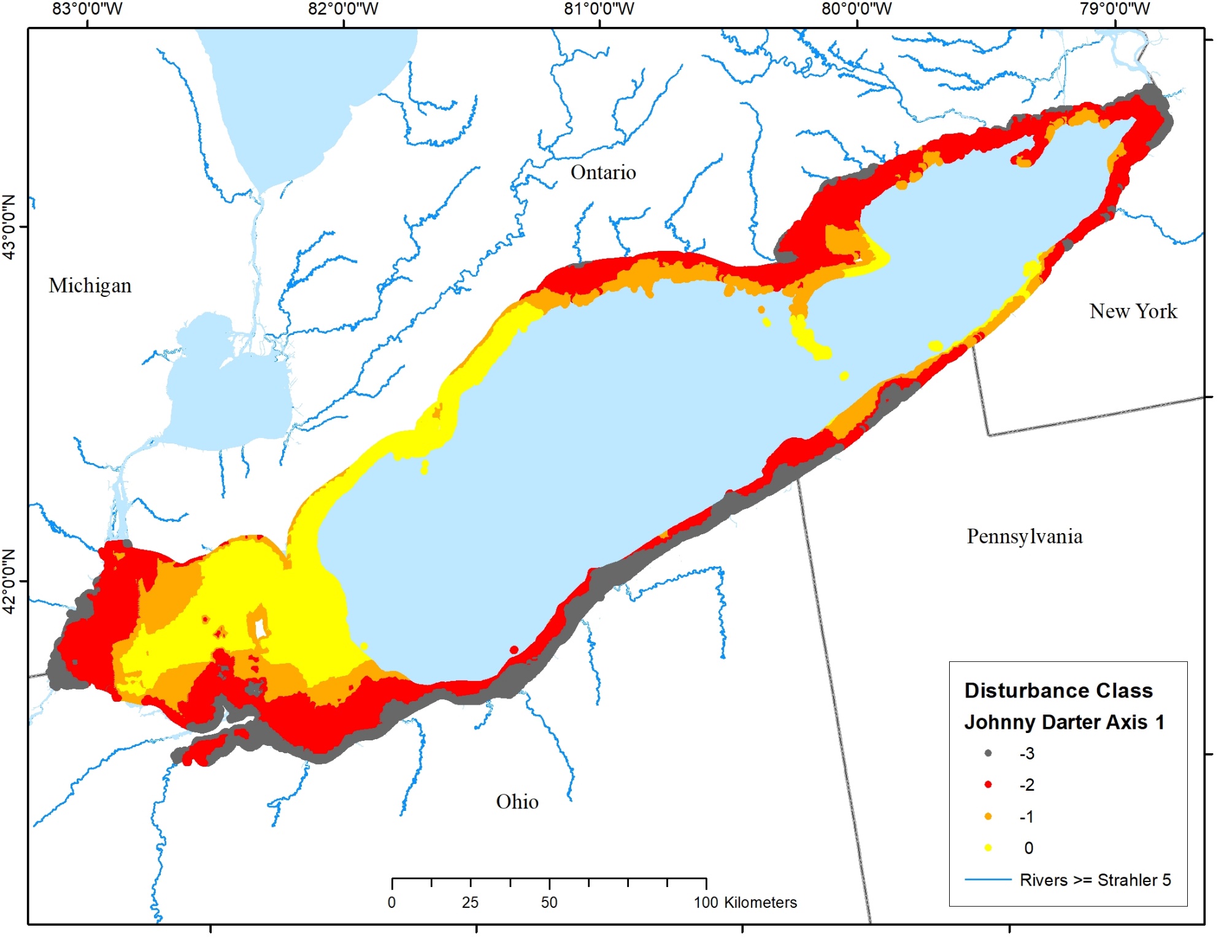
Fig. 4. a.

Fig. 4.b.


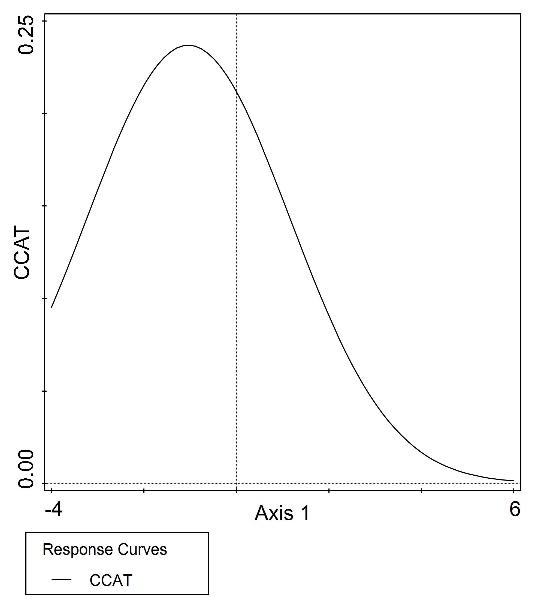

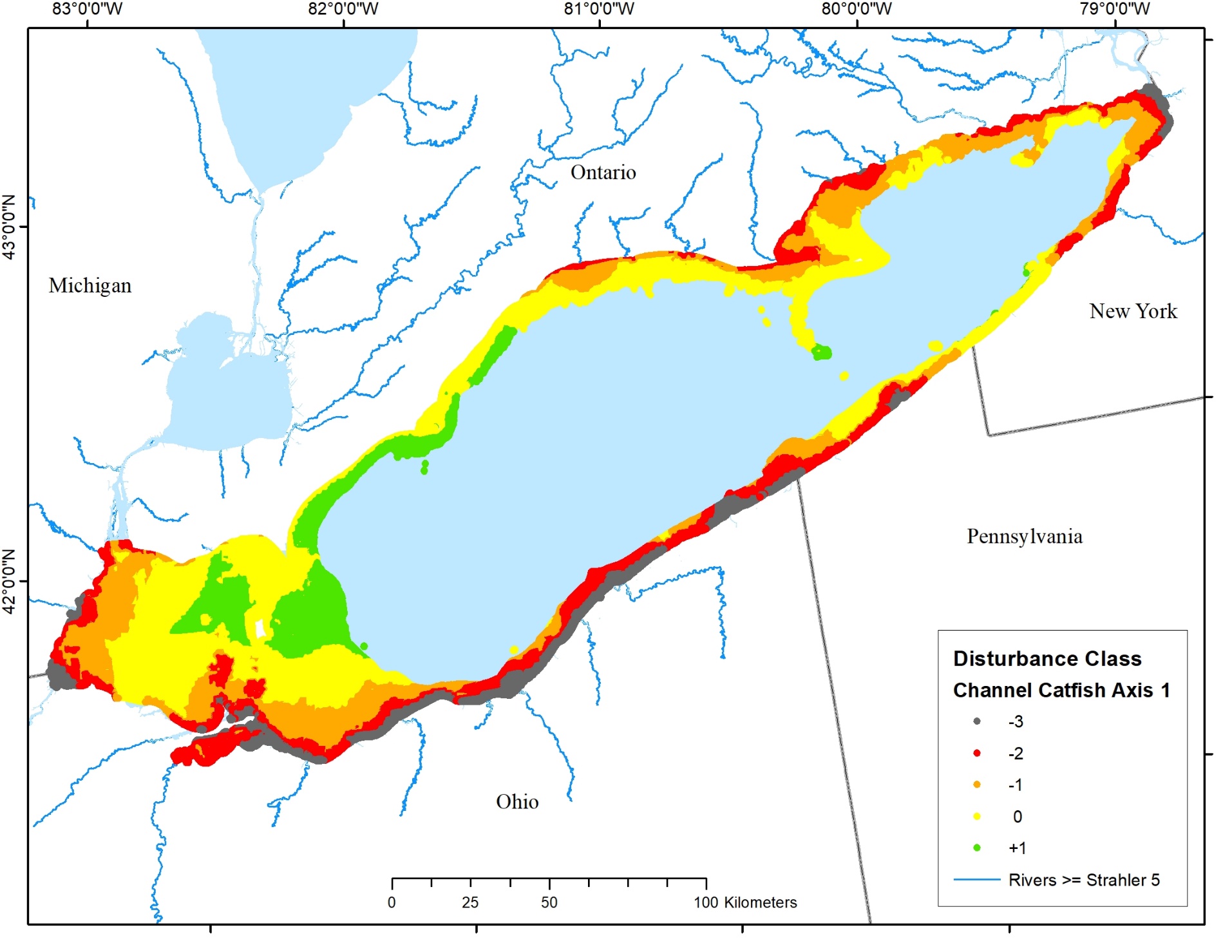


Fig. S5.a


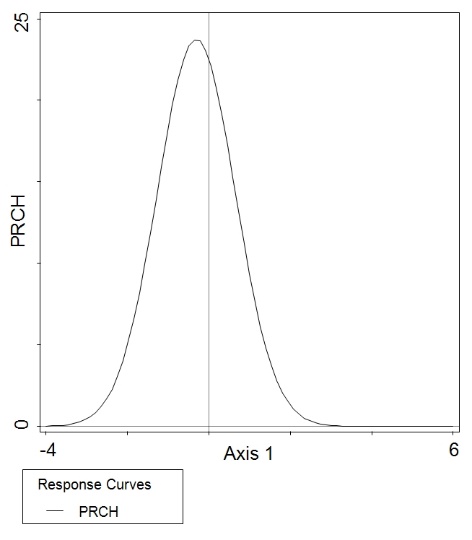

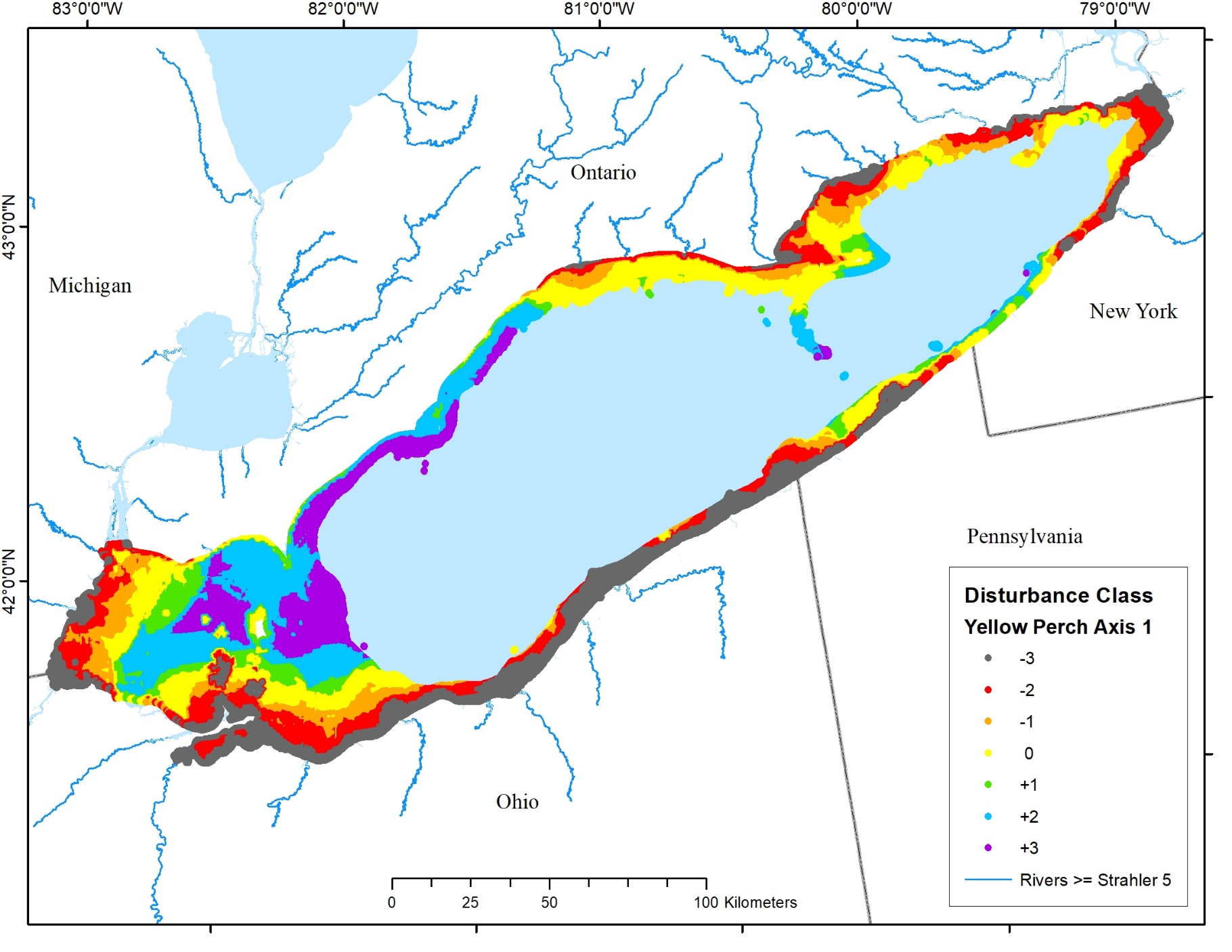


Fig. 5. b.


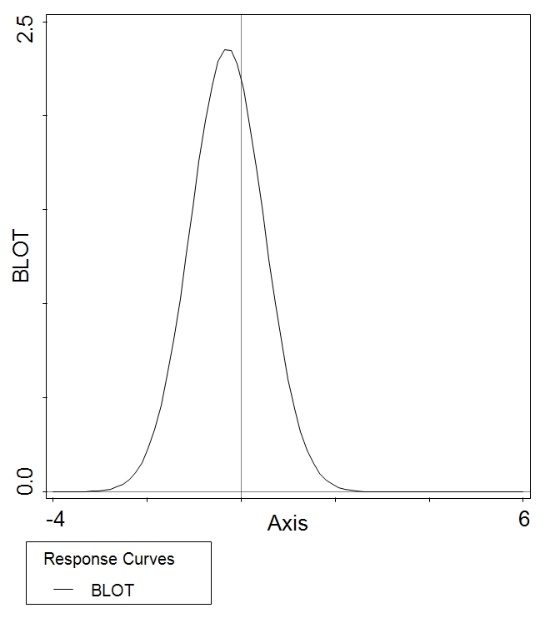

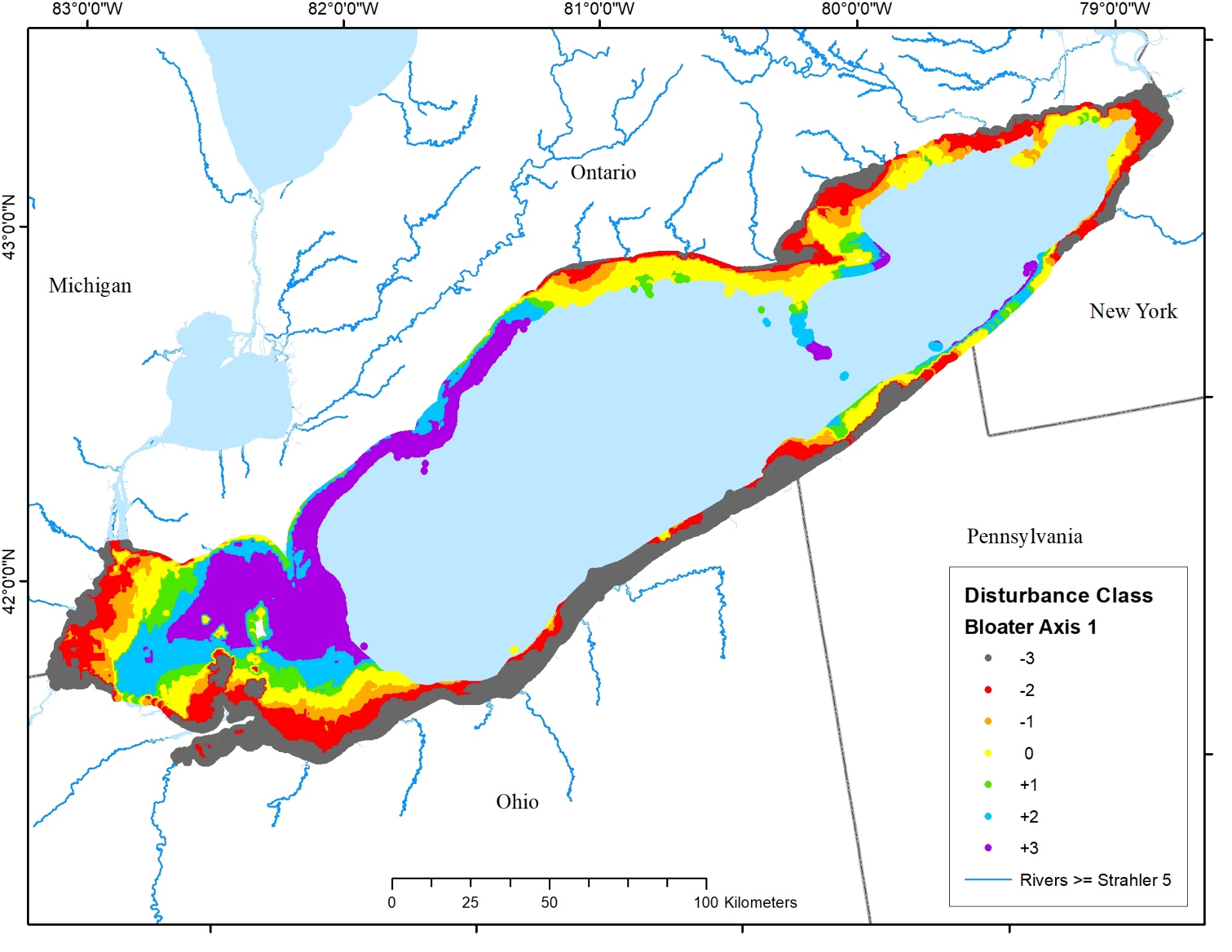

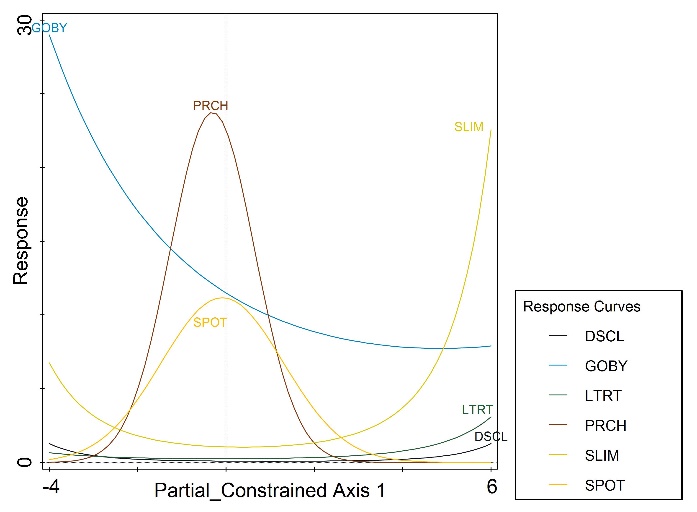

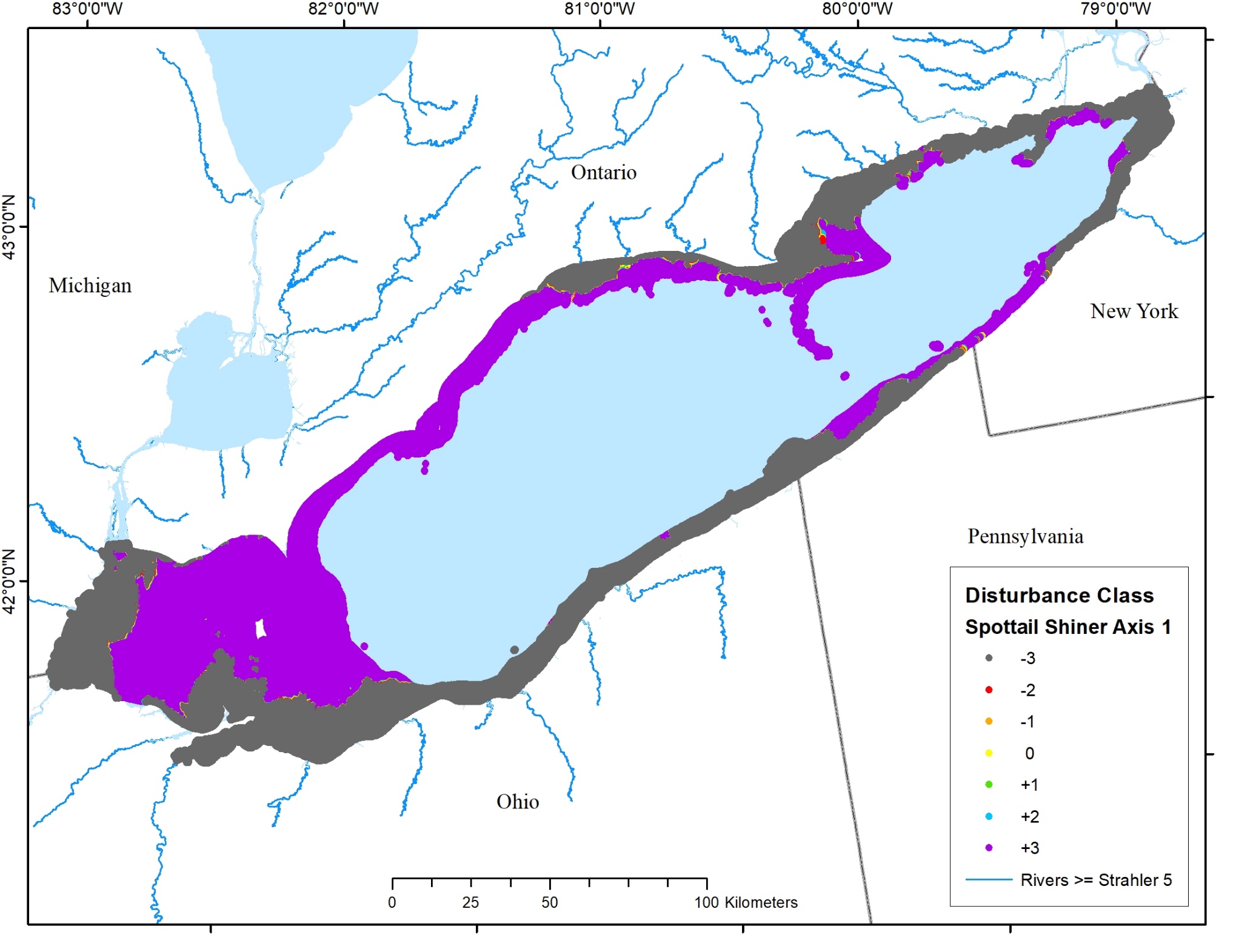
Fig. S5. c.


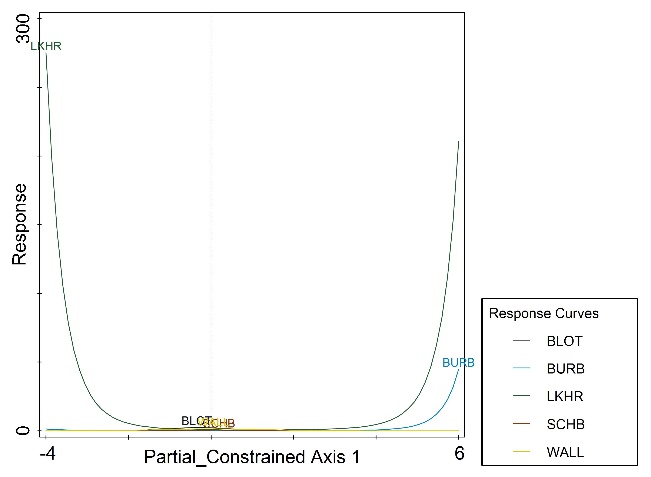

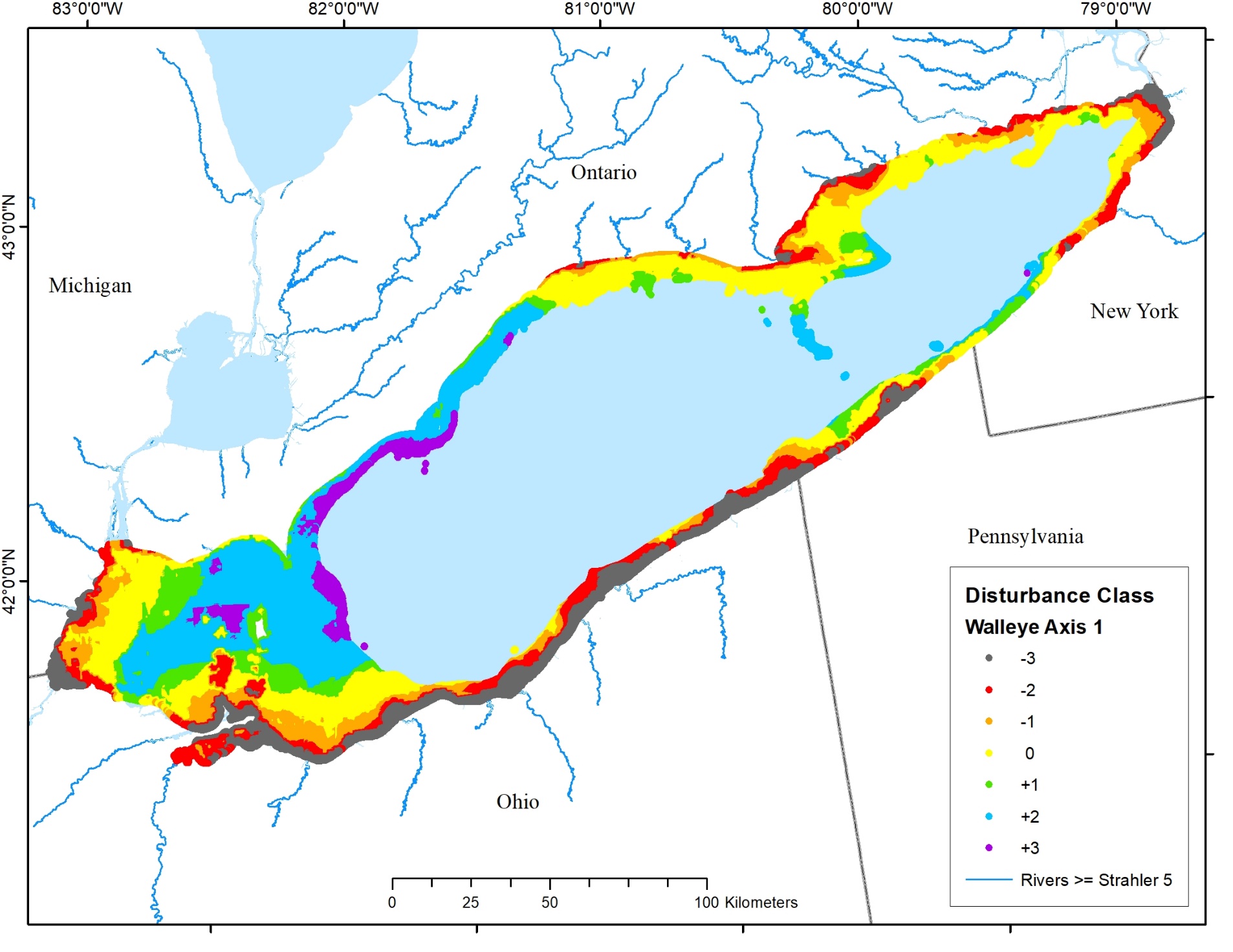
Fig. 5. d.

Fig. 5. e.


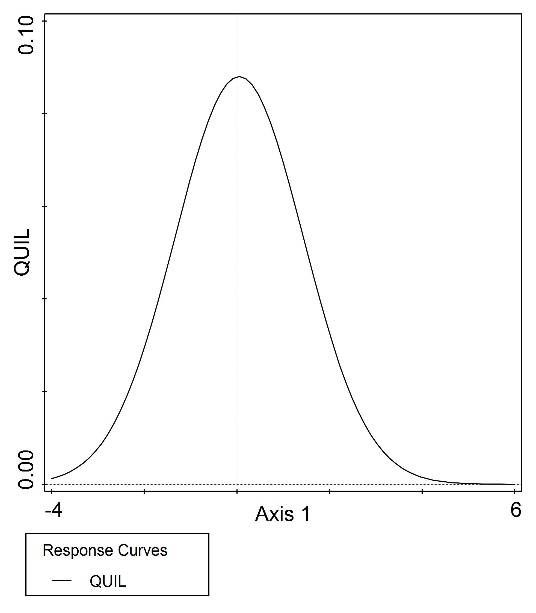

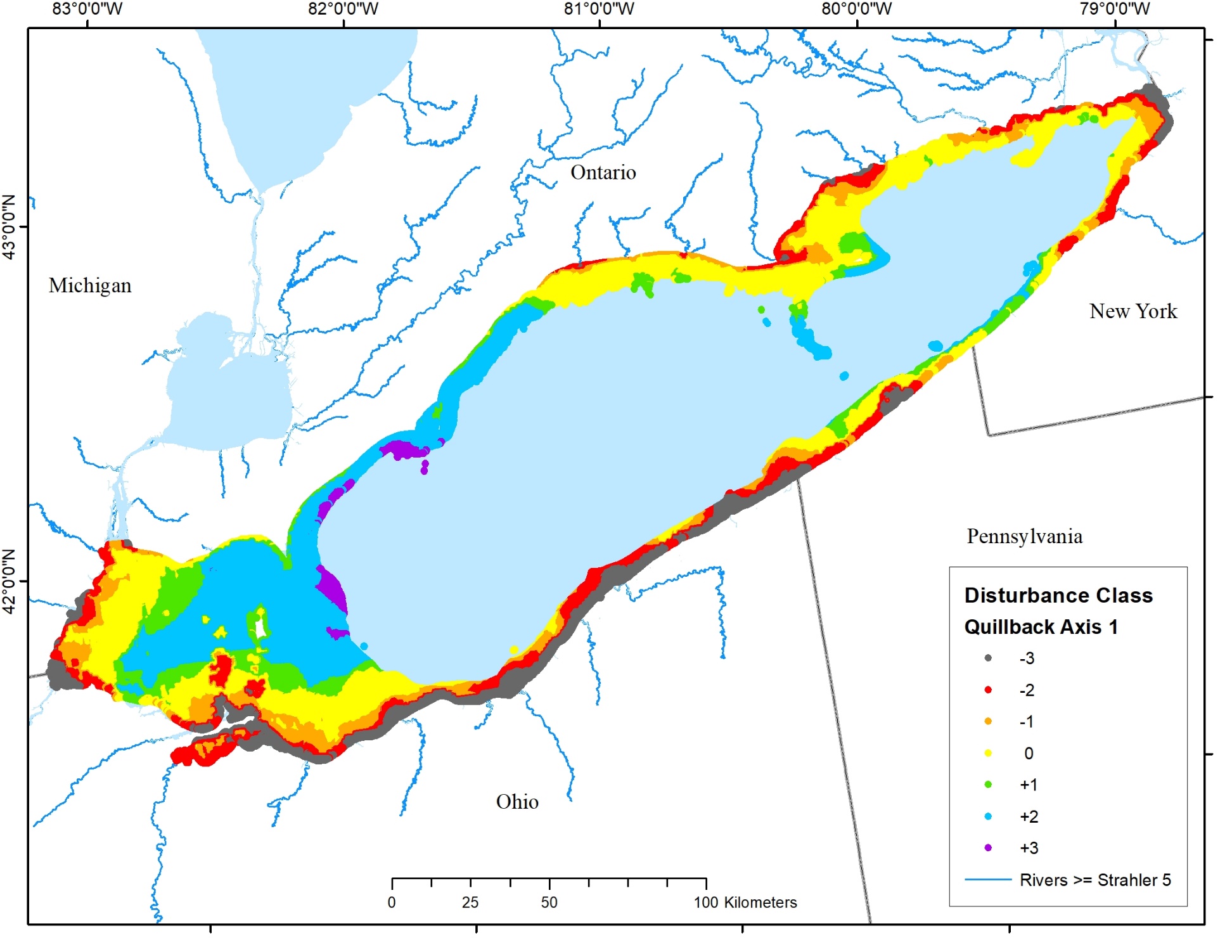


Fig. 5. f.


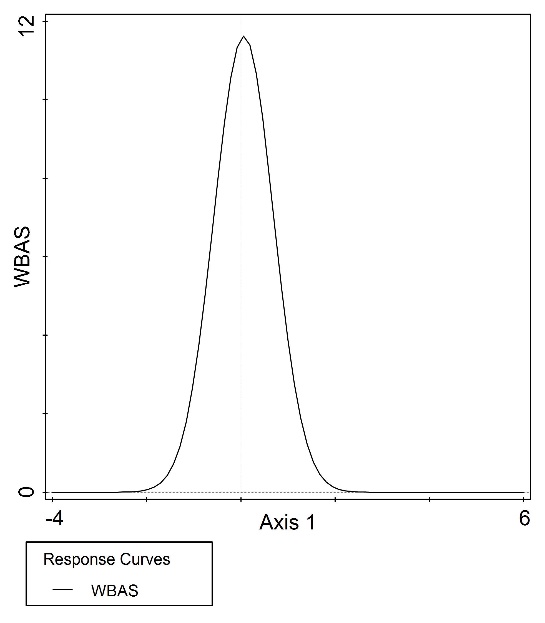

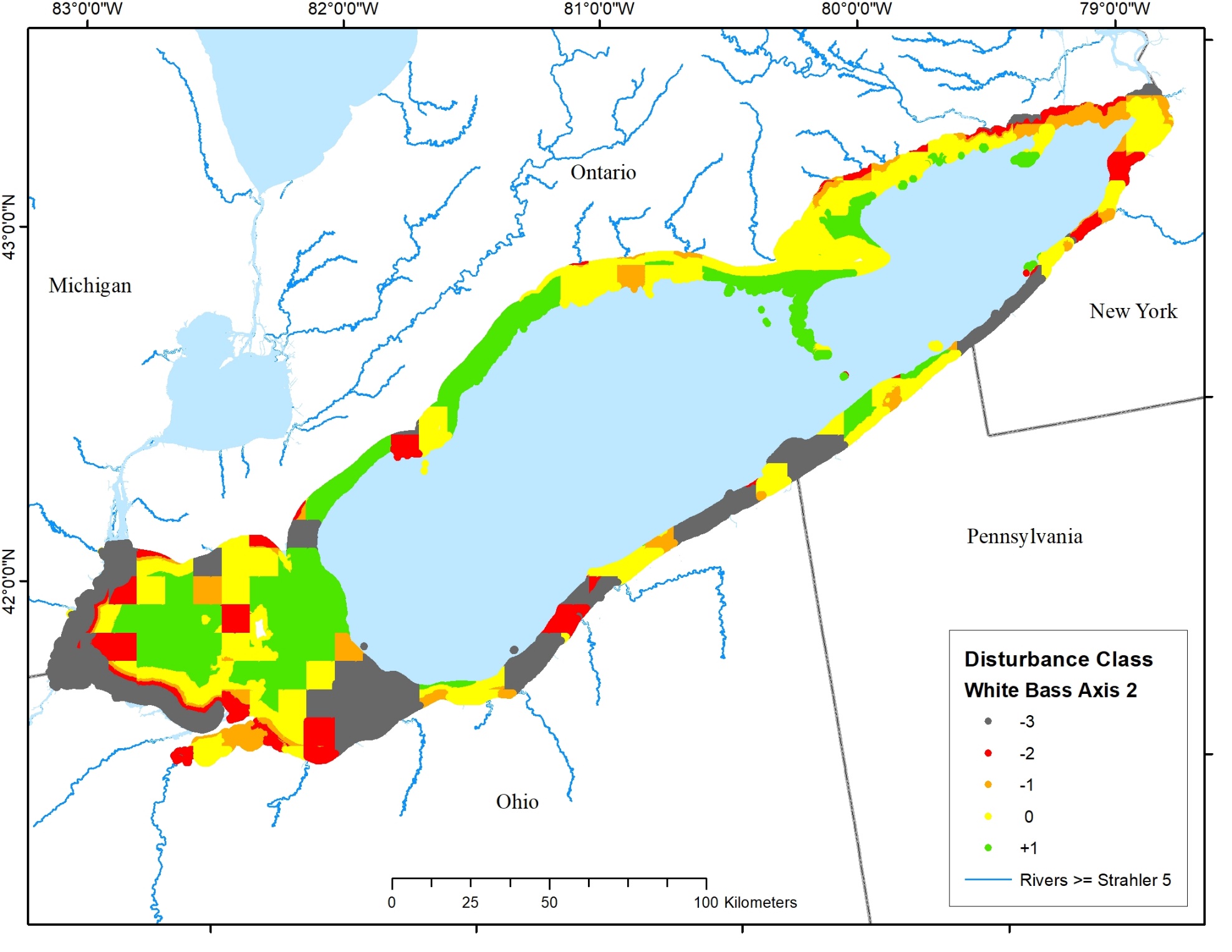
Fig. 5.g.


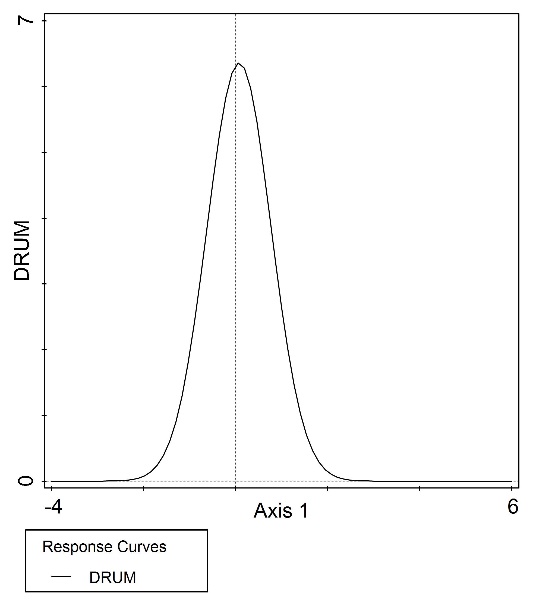

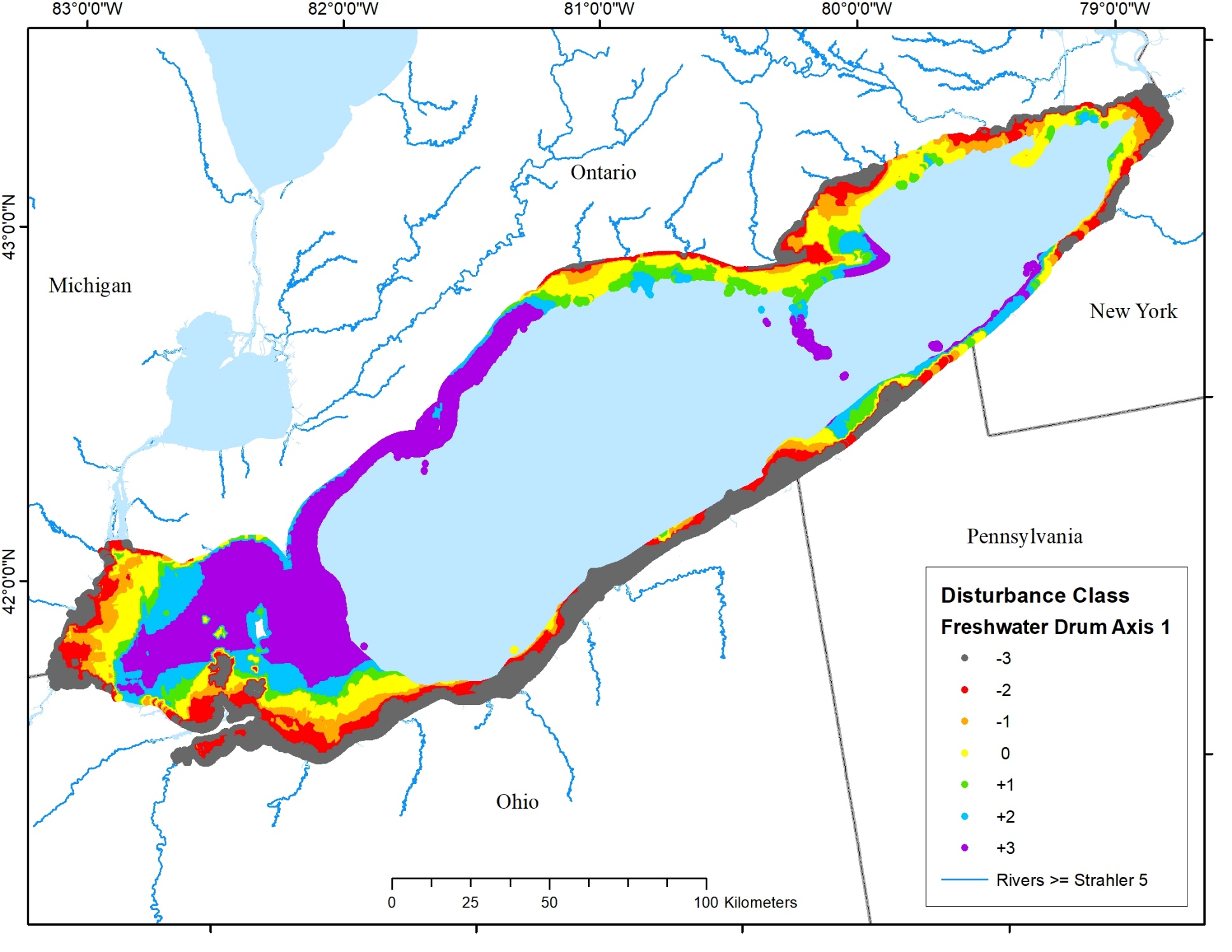


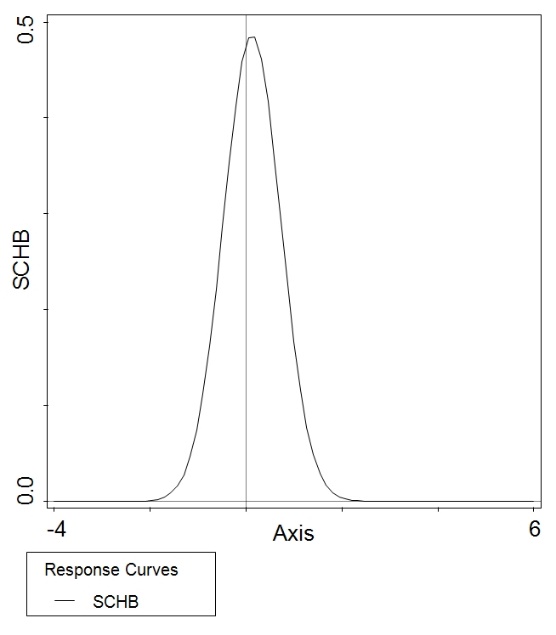

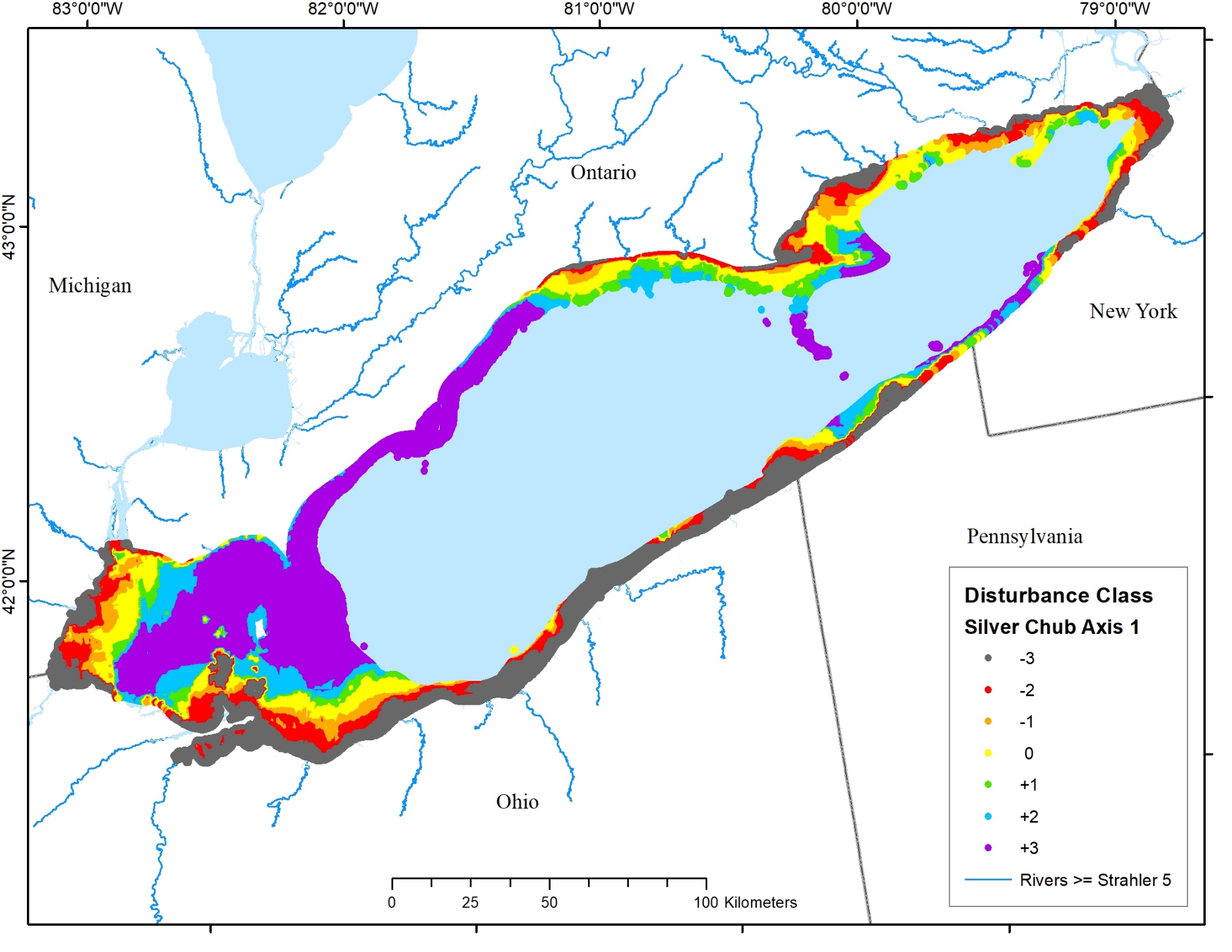
Fig. 5.h.


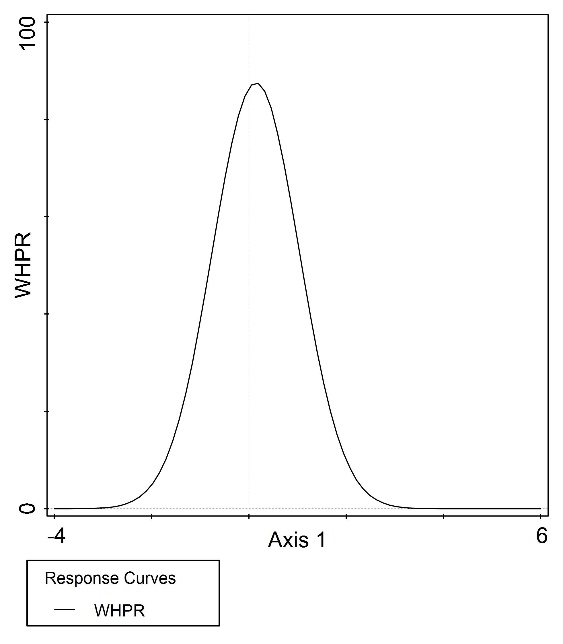

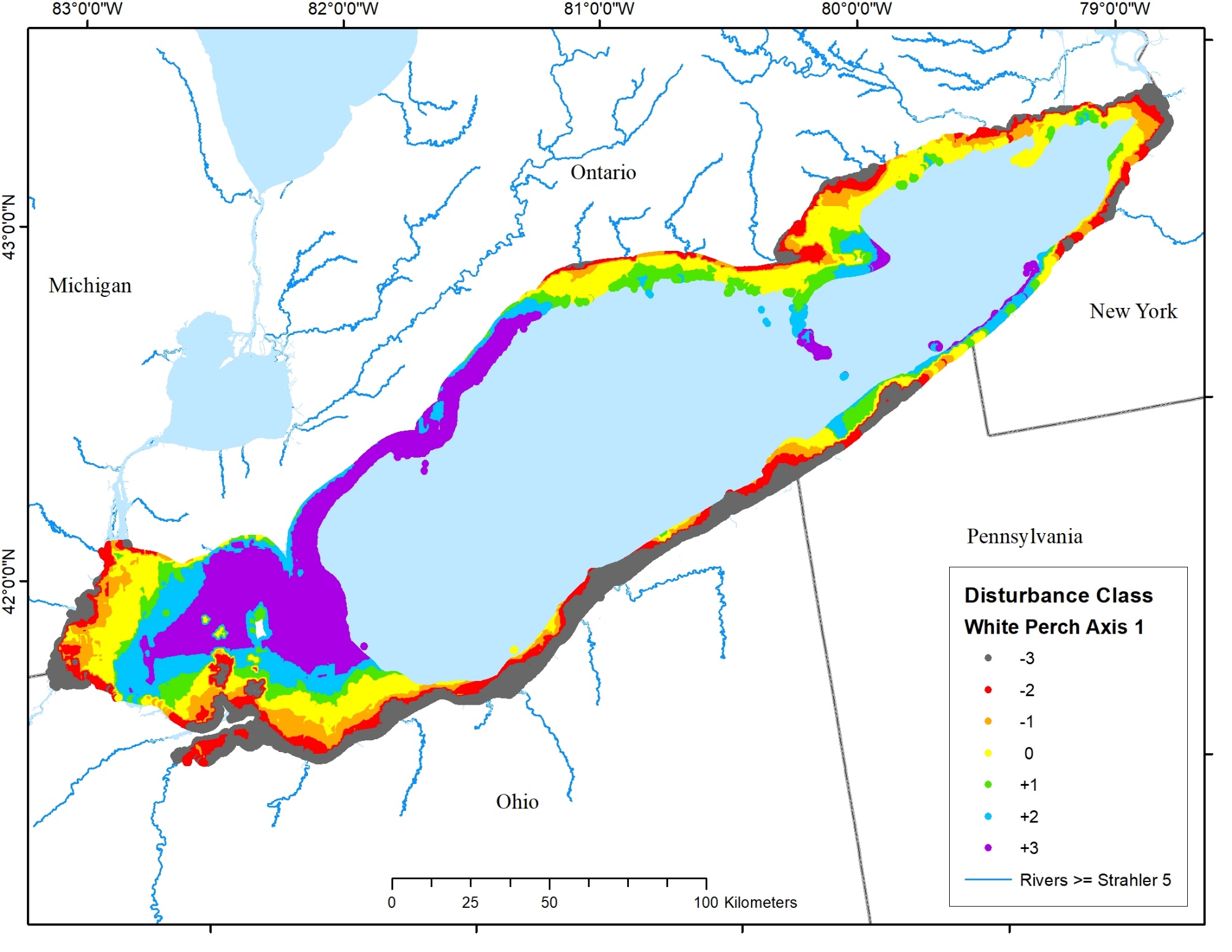
Fig. 5.i.


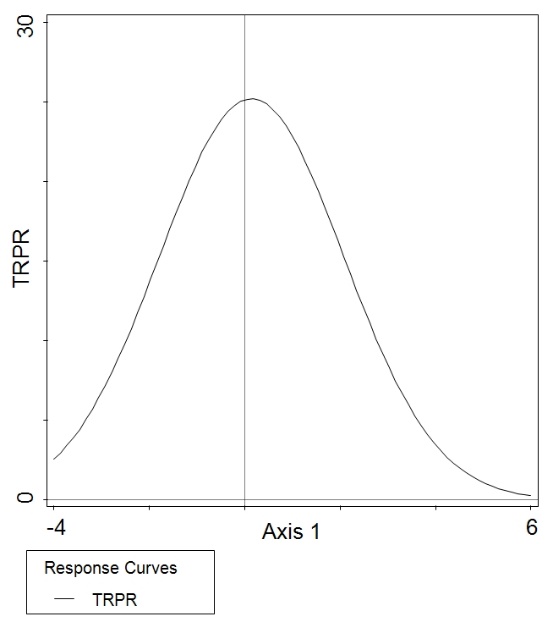

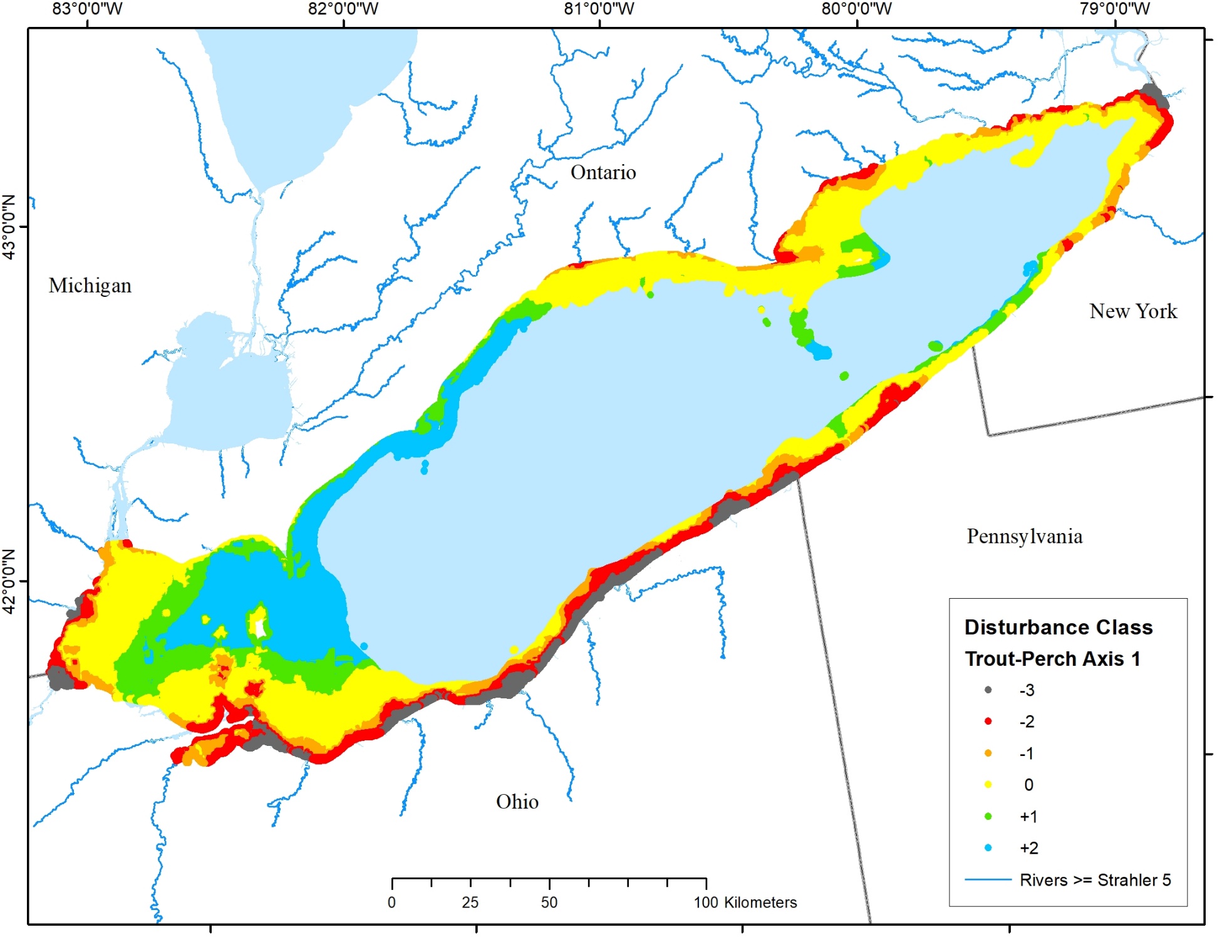
Fig. 5.j.

Fig. 5.k.


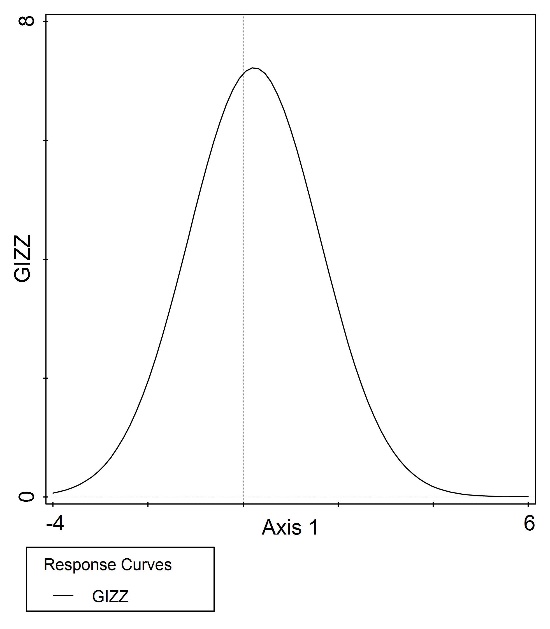

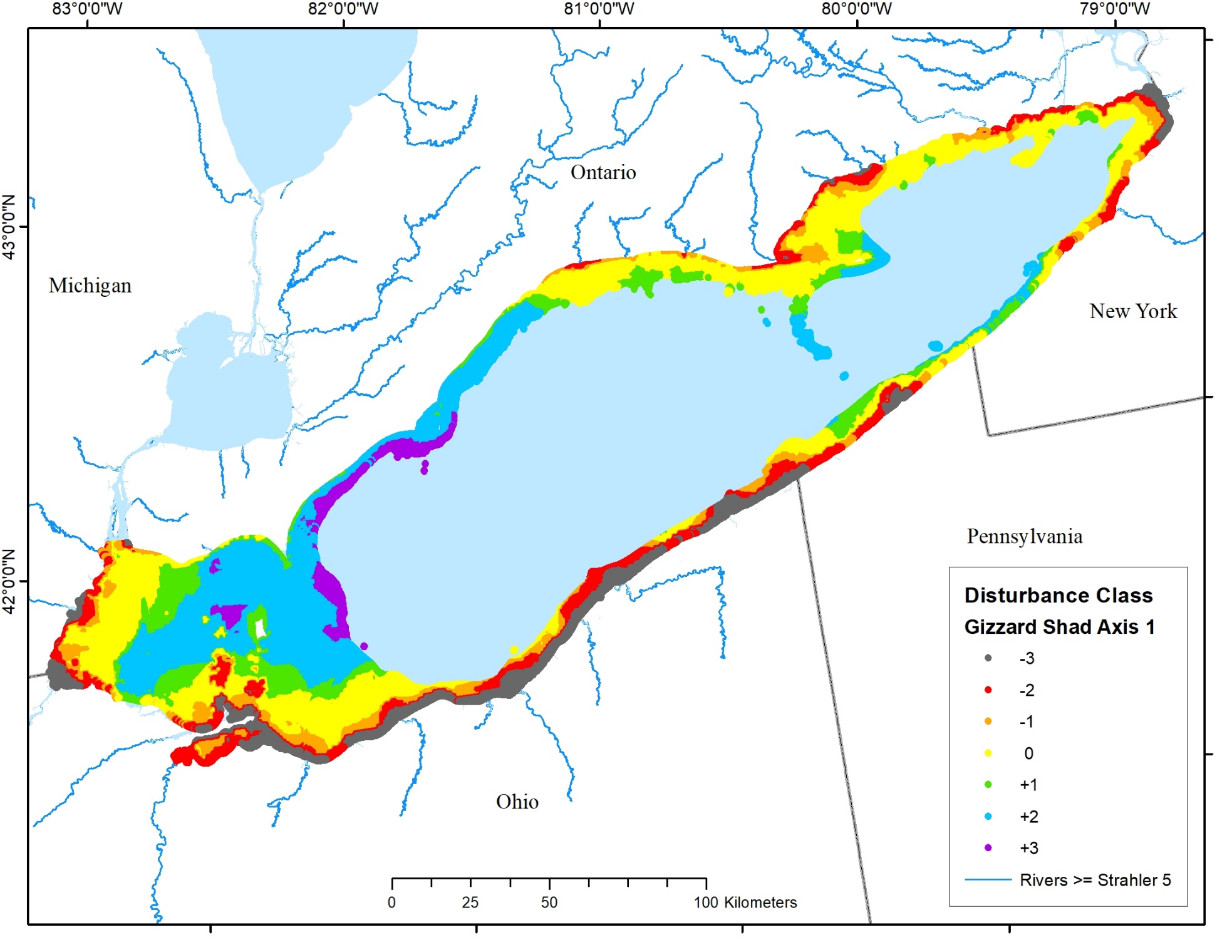


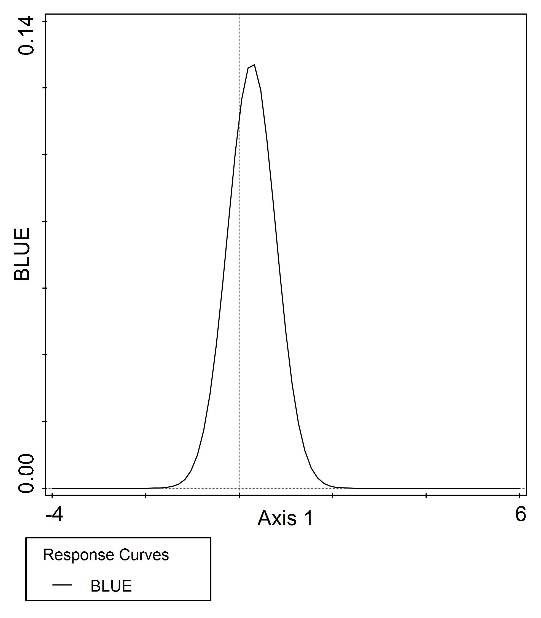

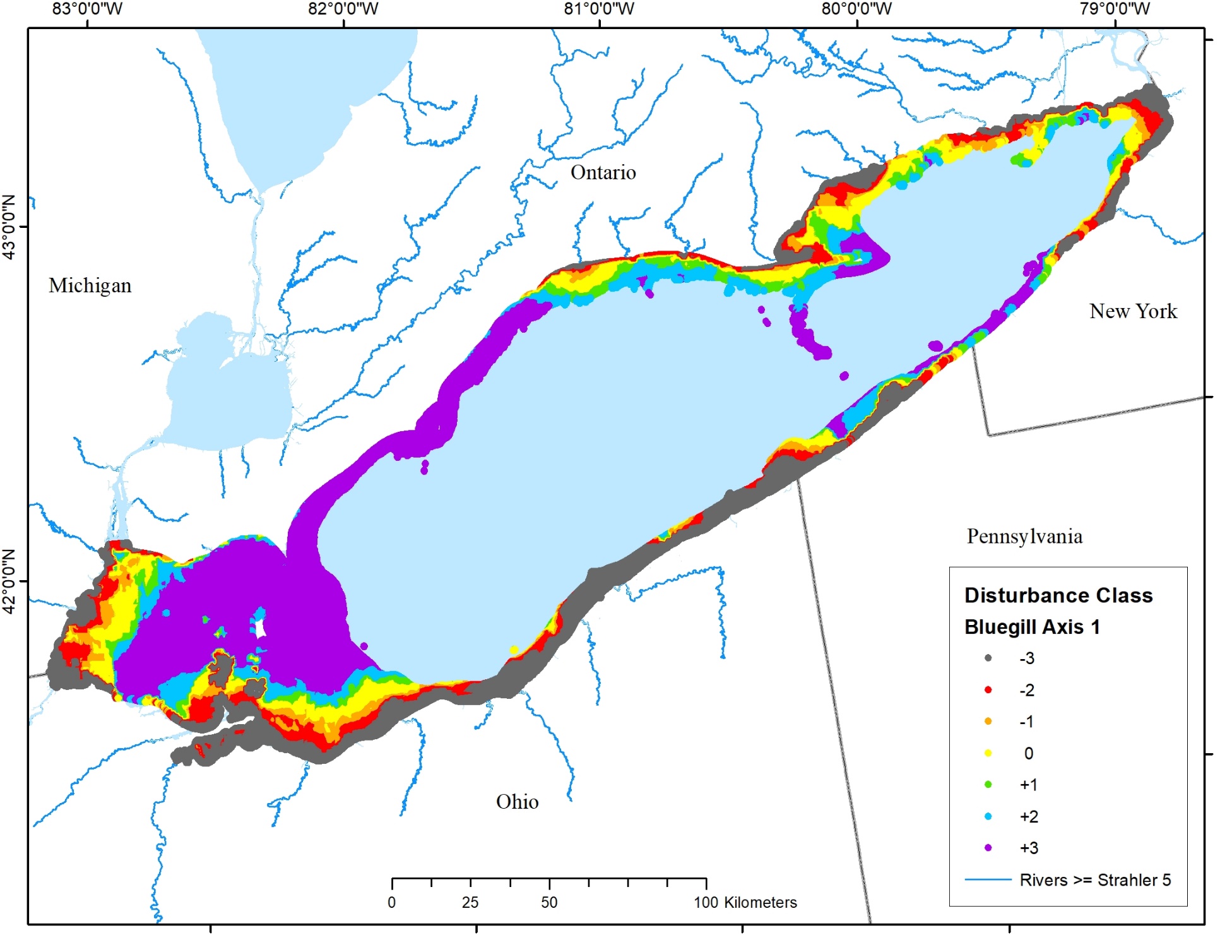
Fig. 5.l.

Fig. 5.m.


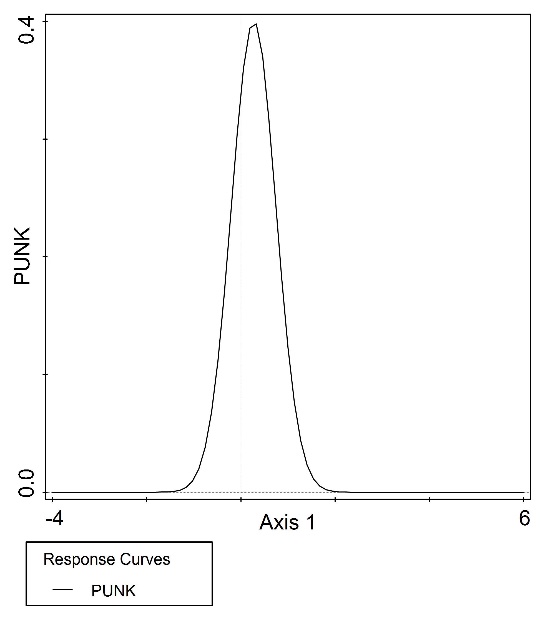

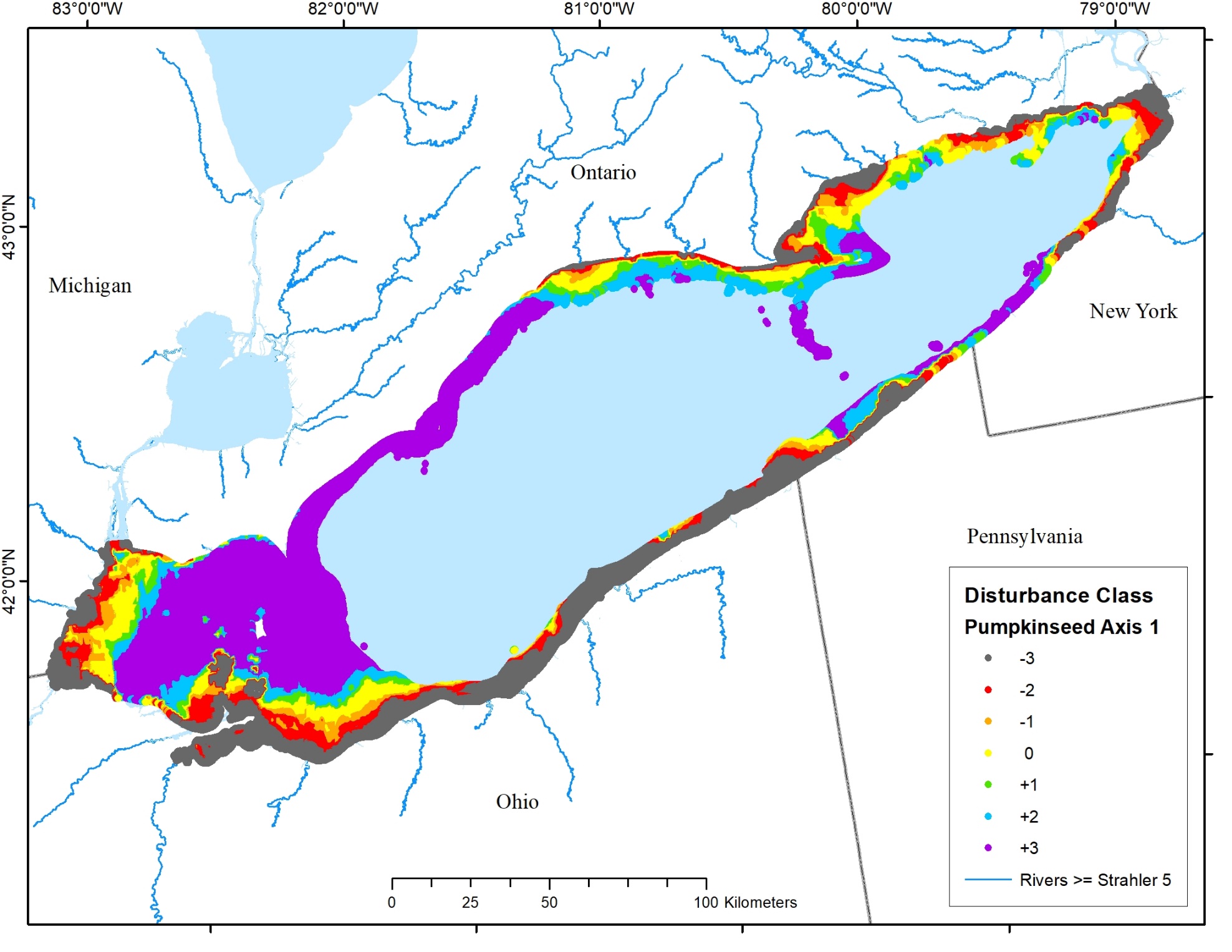


Fig. 5.n.


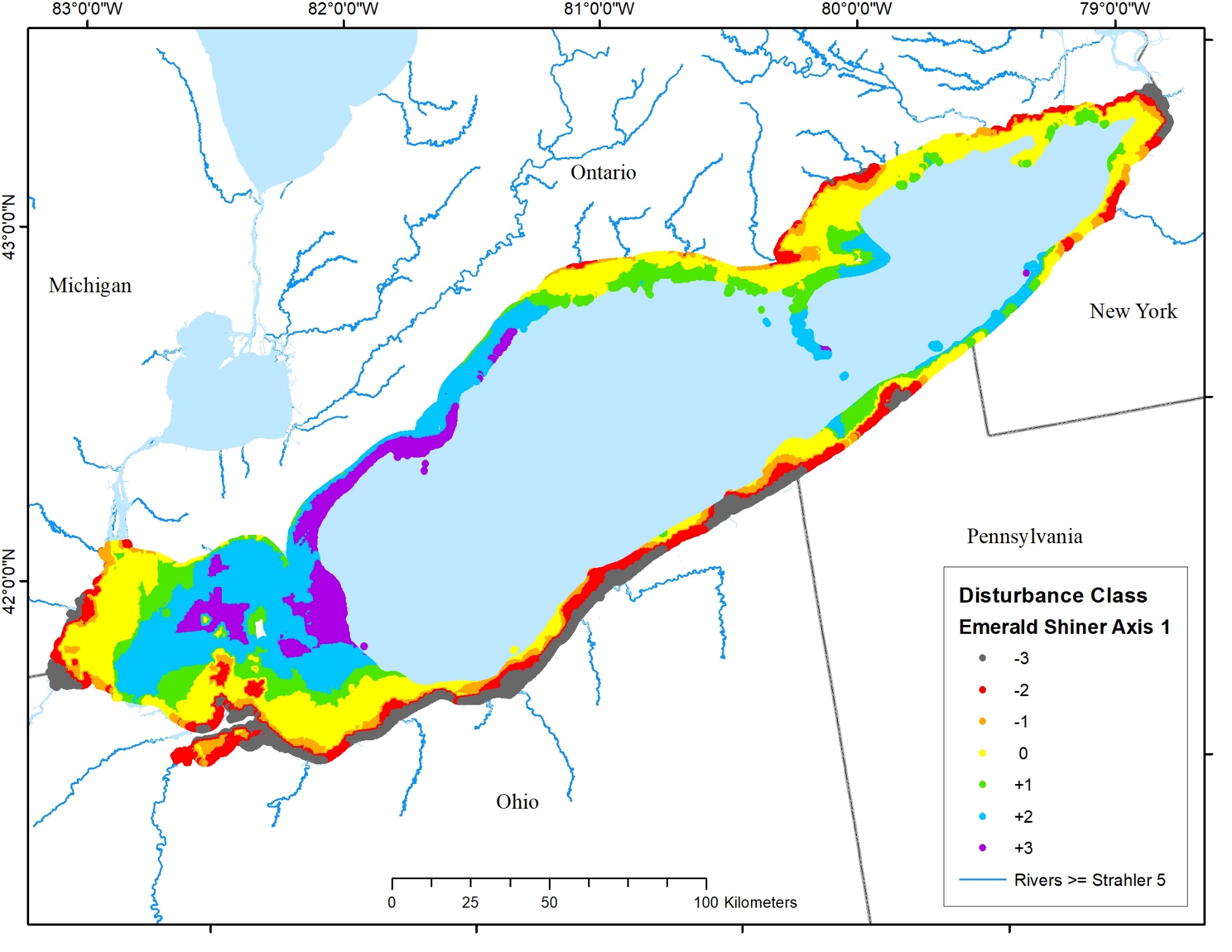

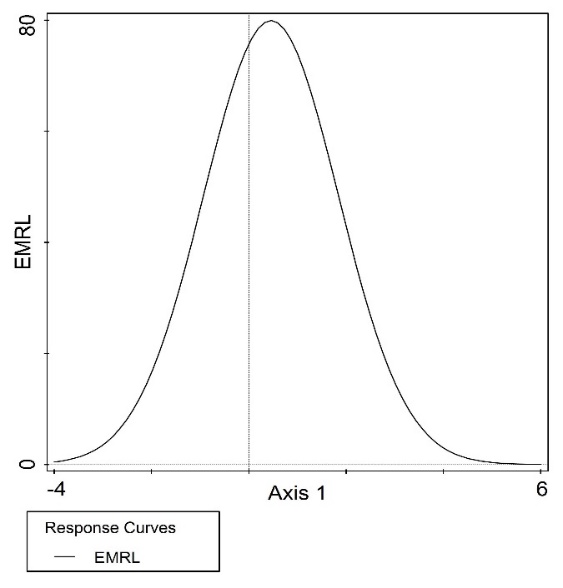


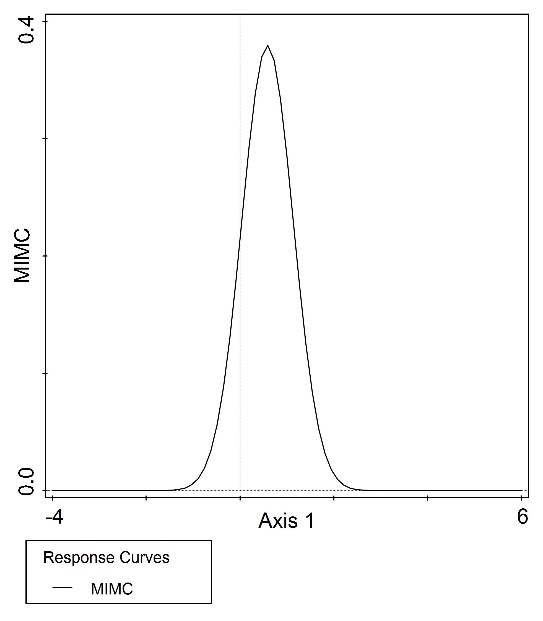

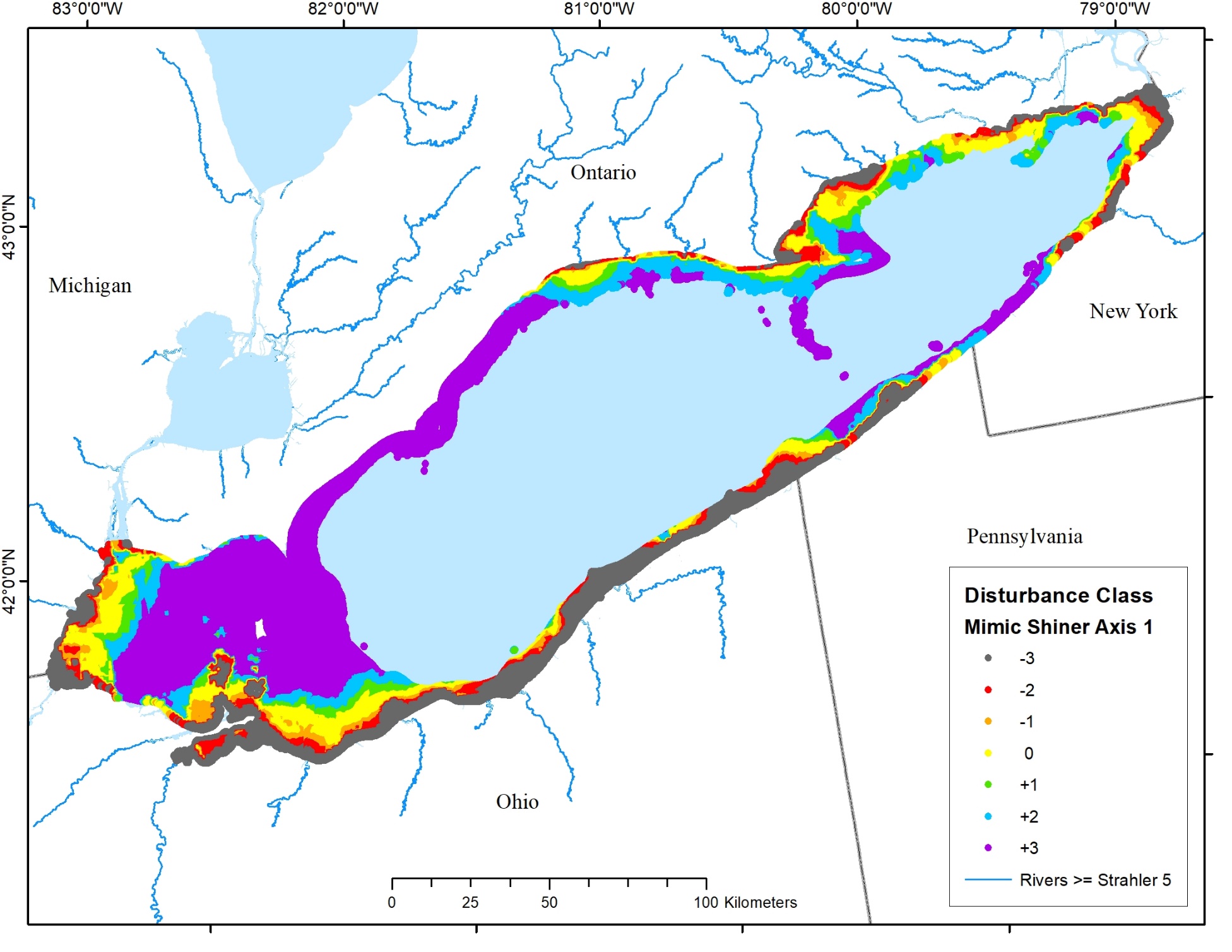
Fig. 6.a.


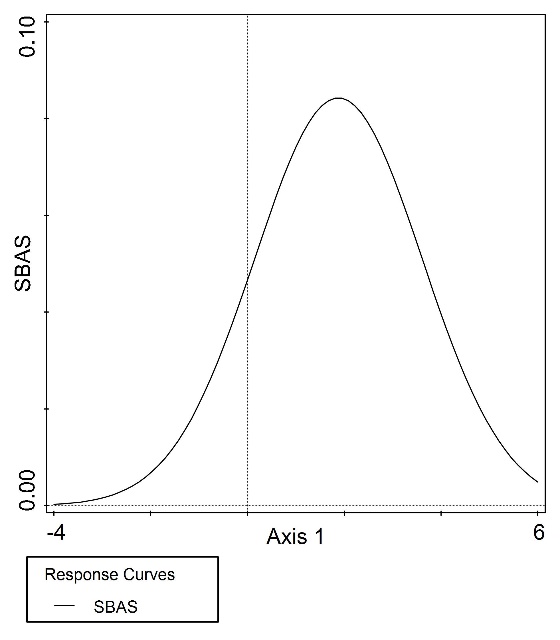

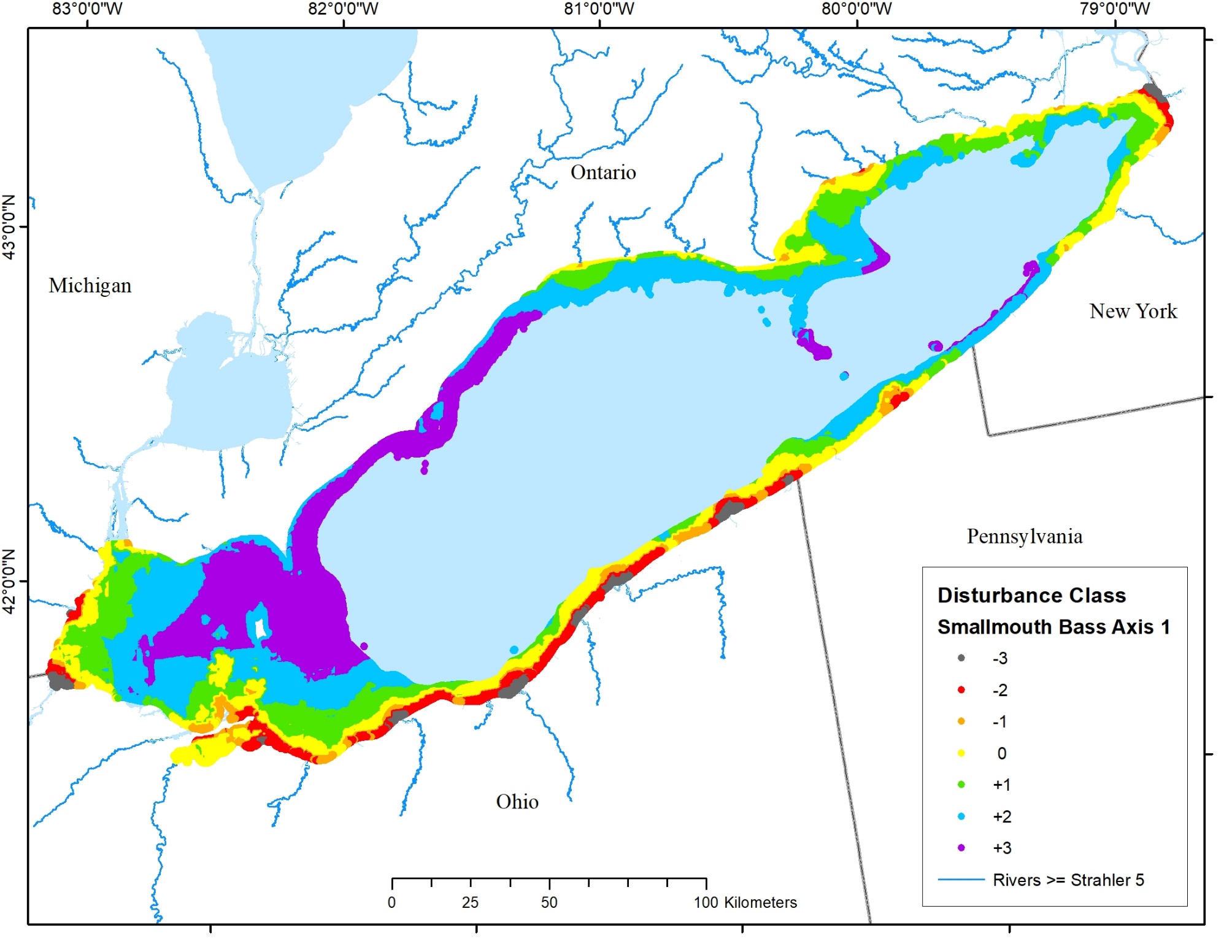
Fig. 6.b.

Fig. 7.a.


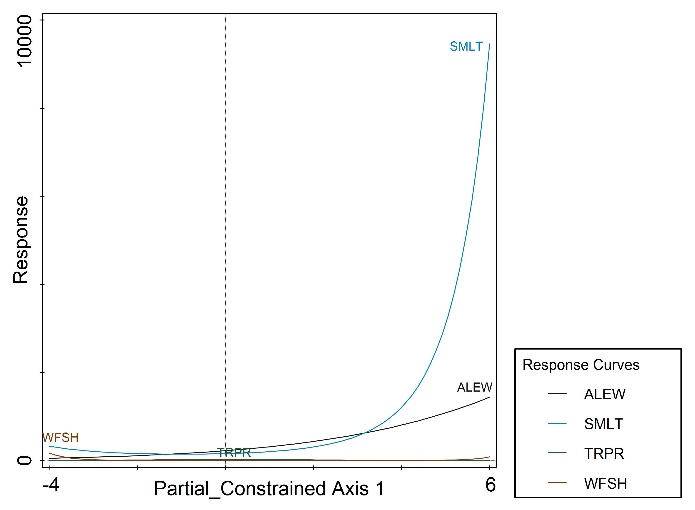

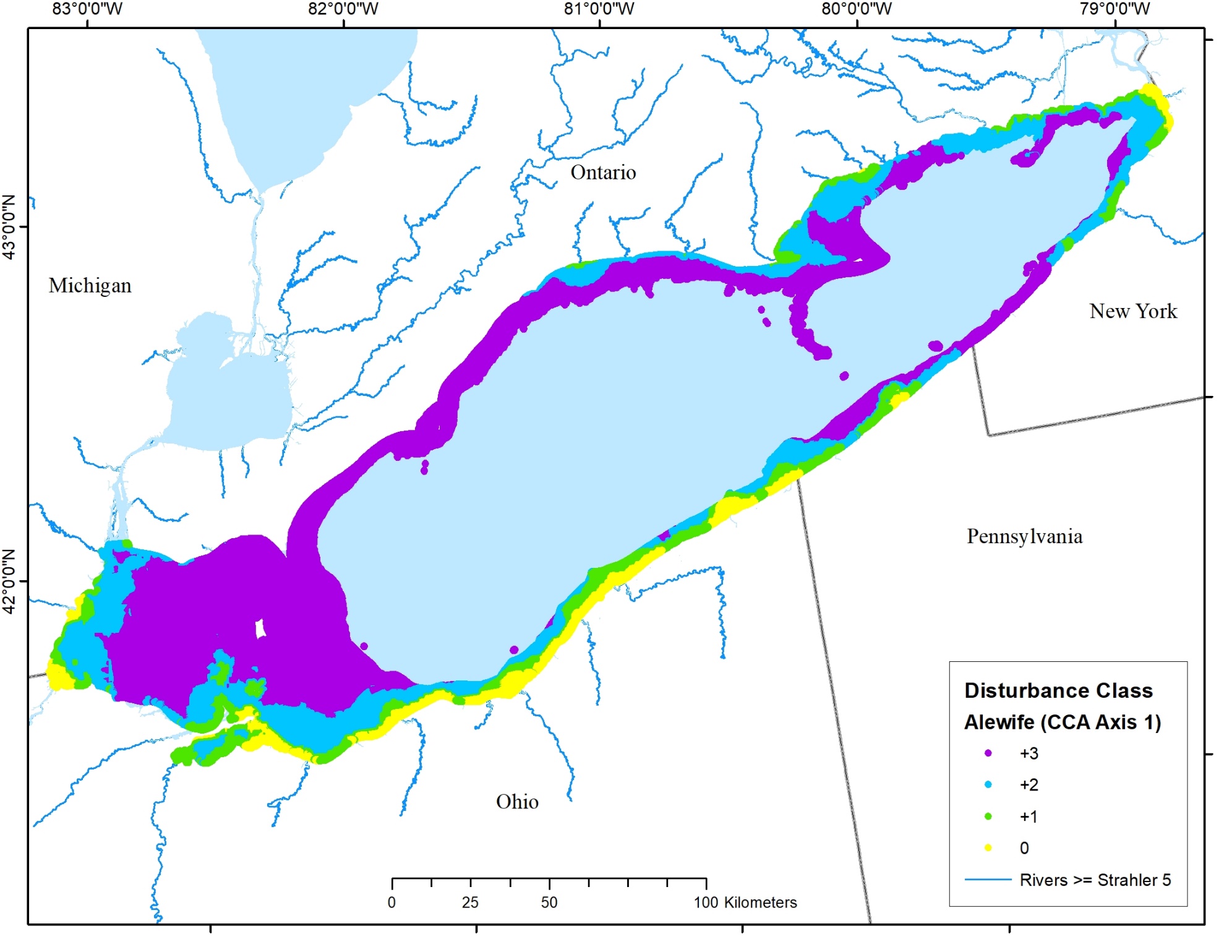


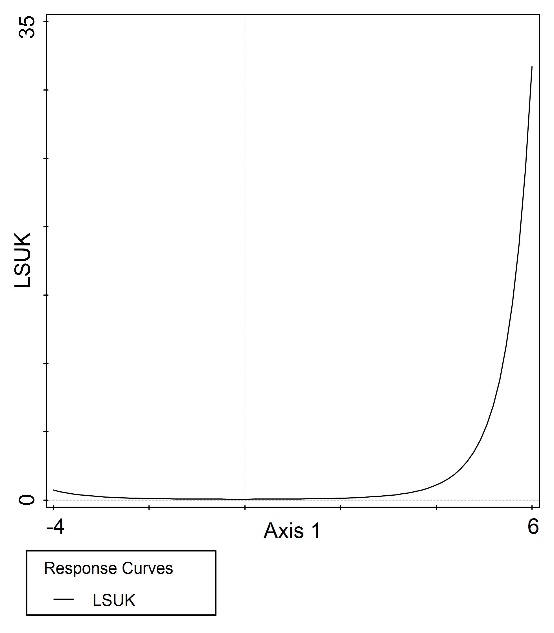

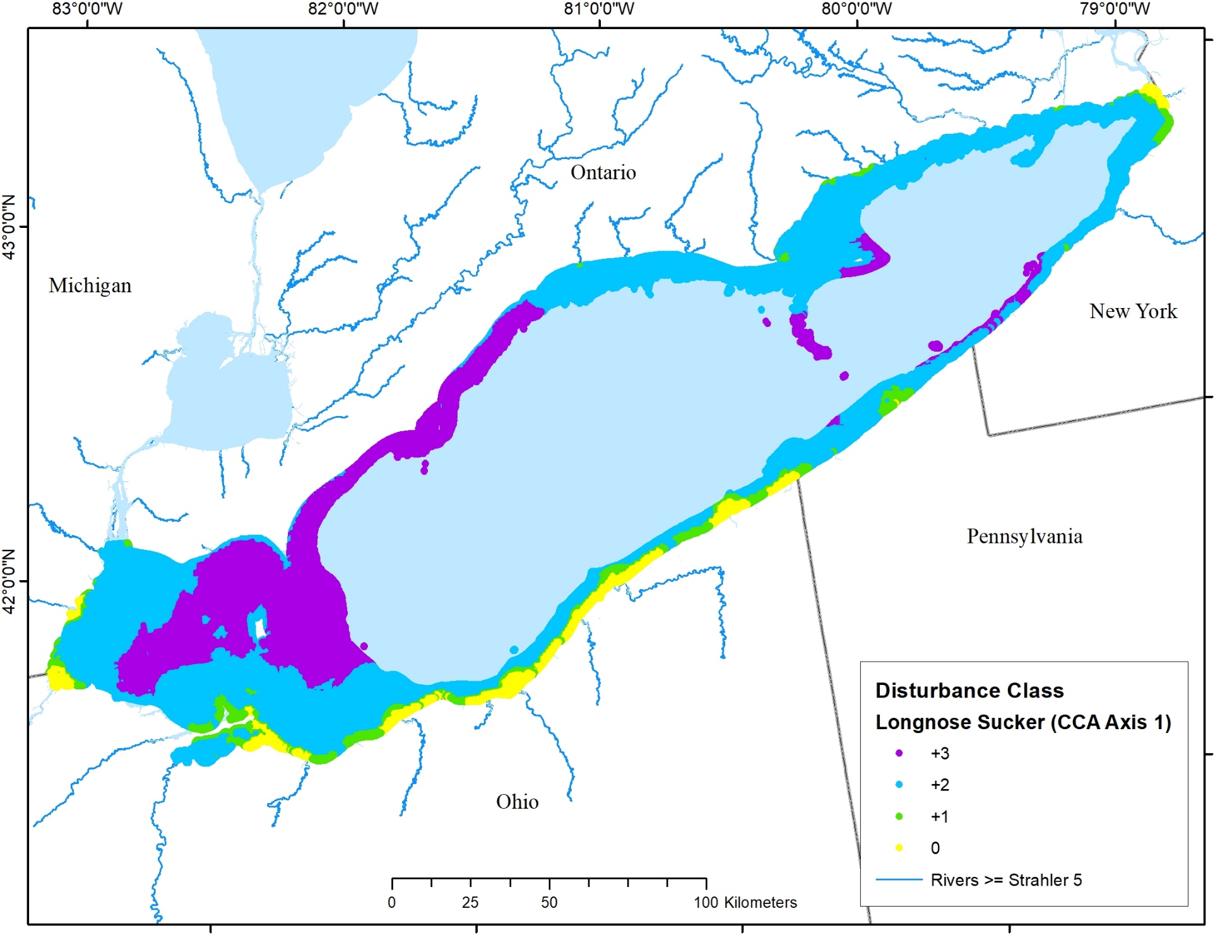
Fig. 7.b.


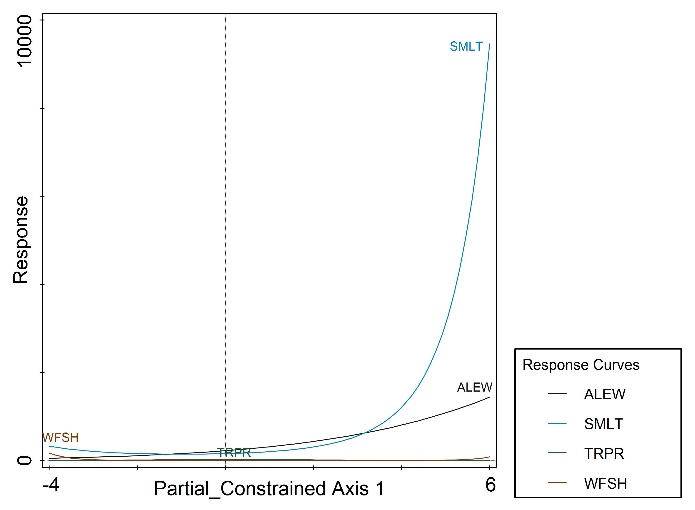

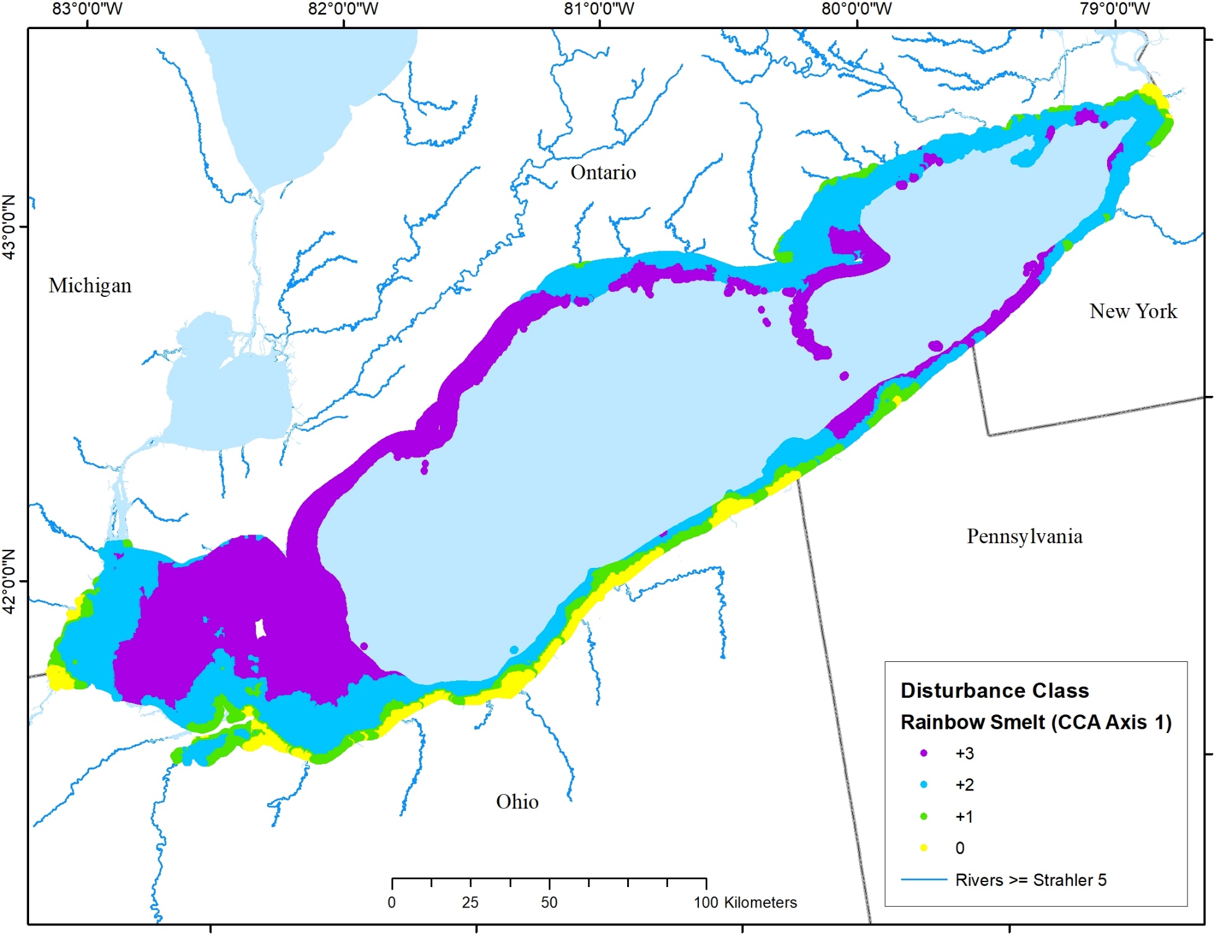
Fig. 7.c.


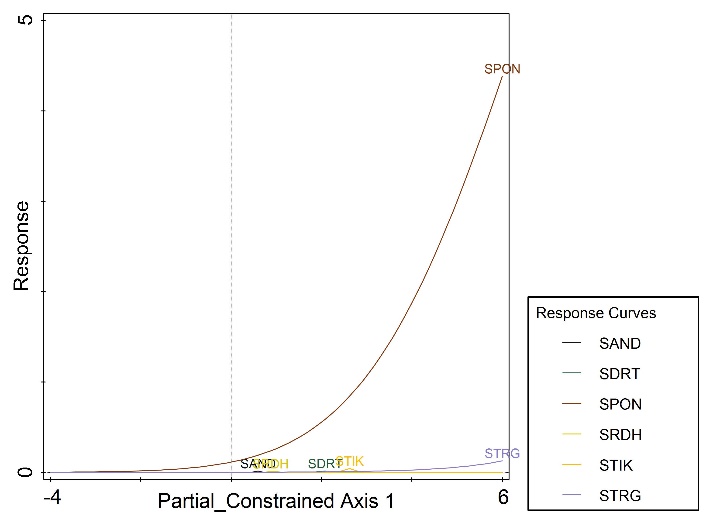

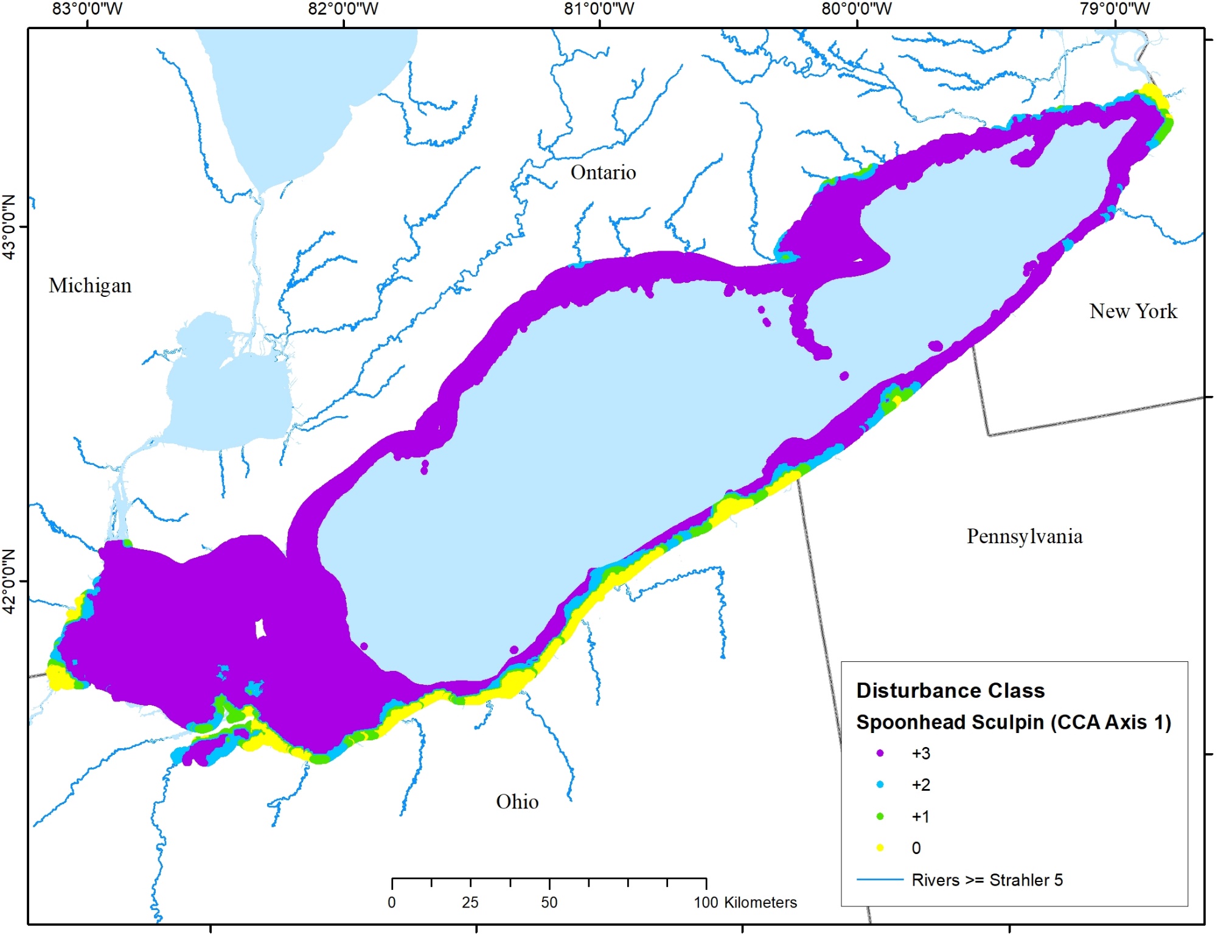
Fig. 7.d.


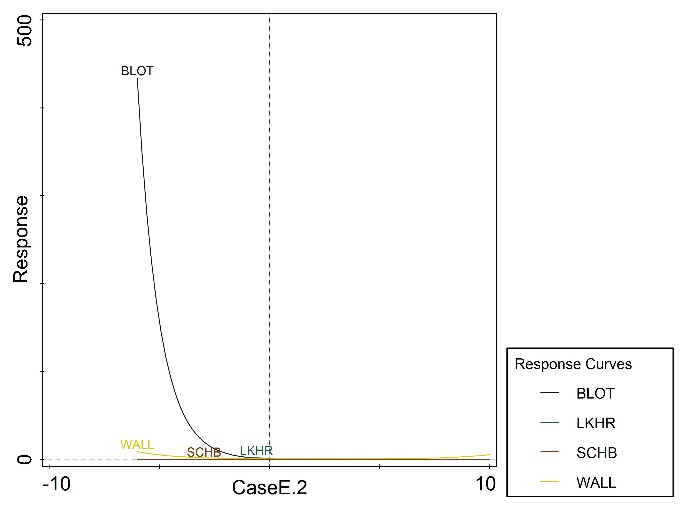

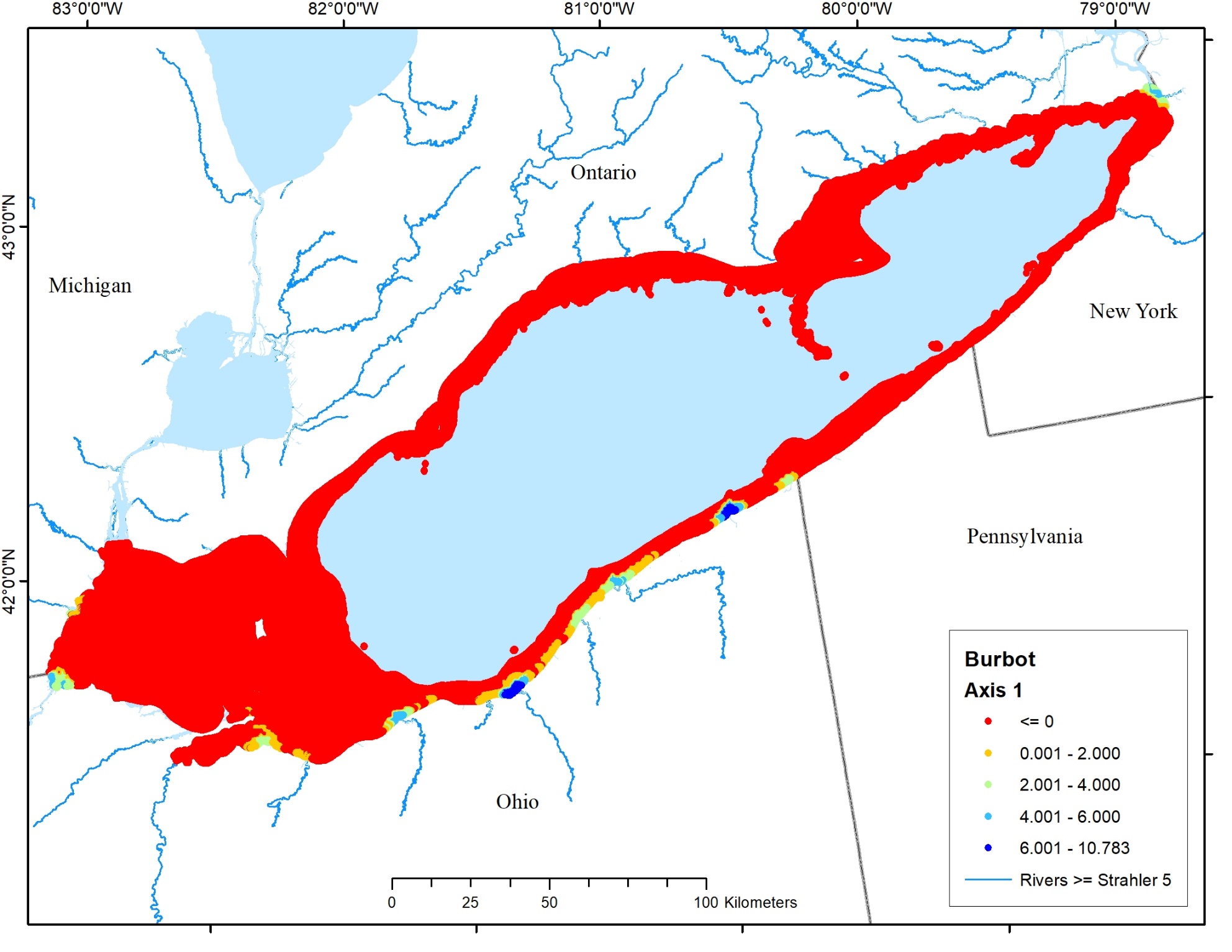
Fig. 8.a.


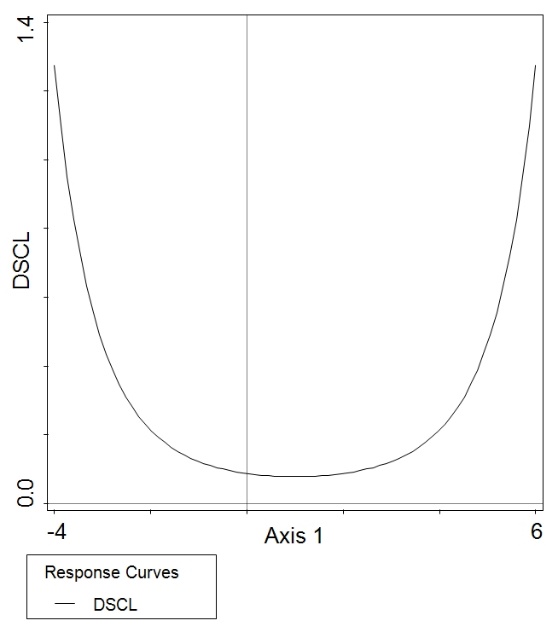

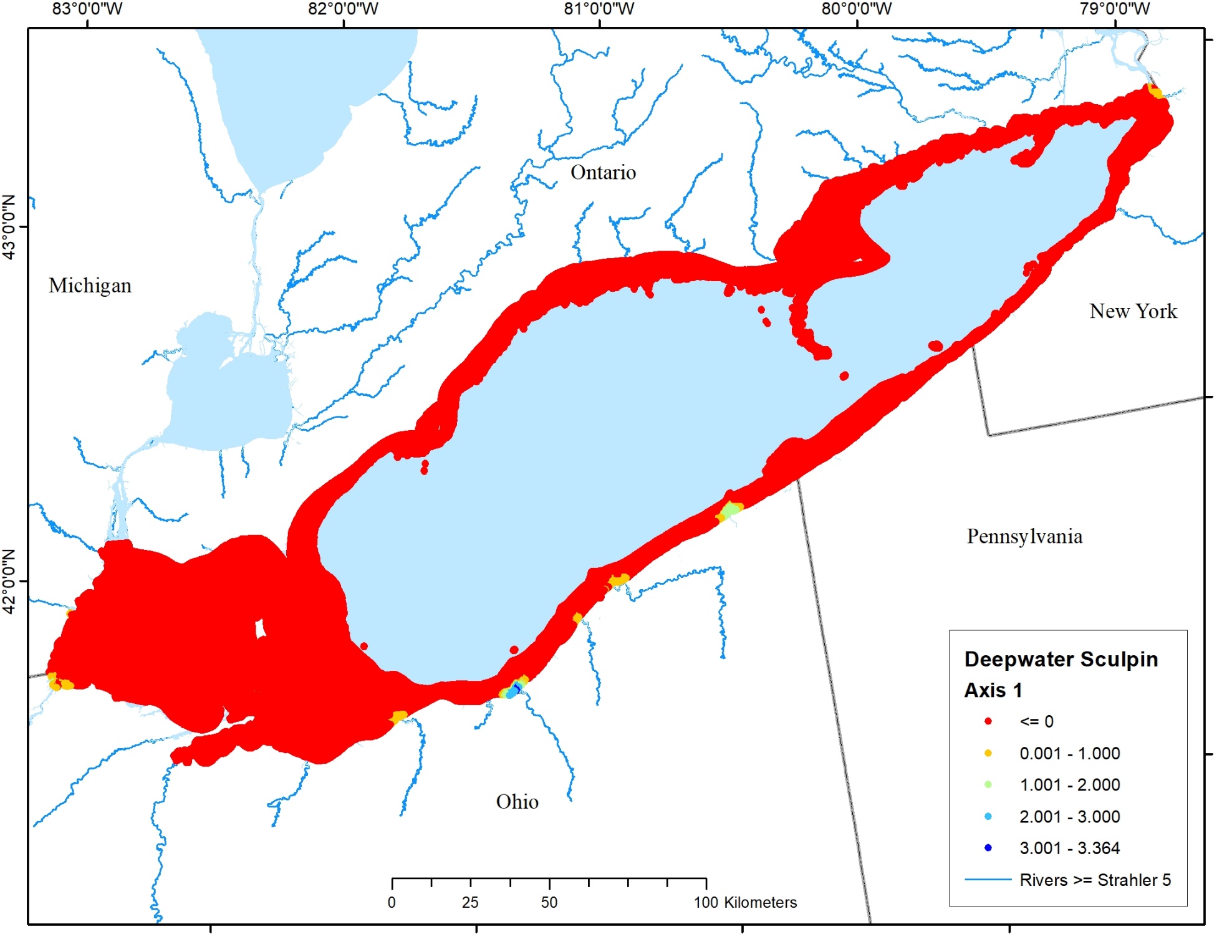
Fig. 8.b.


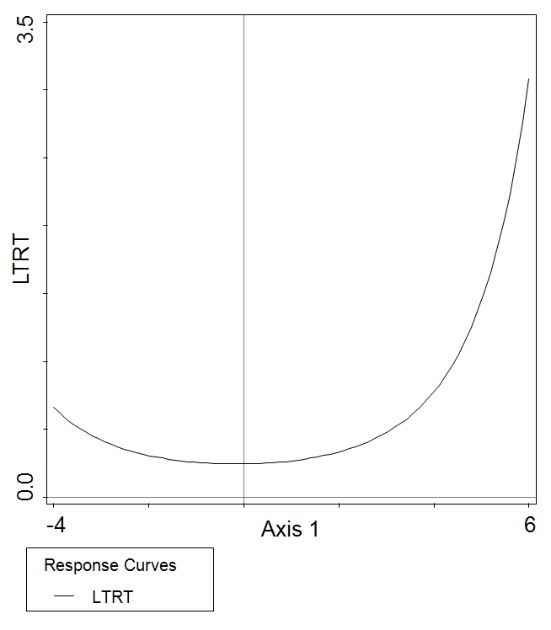

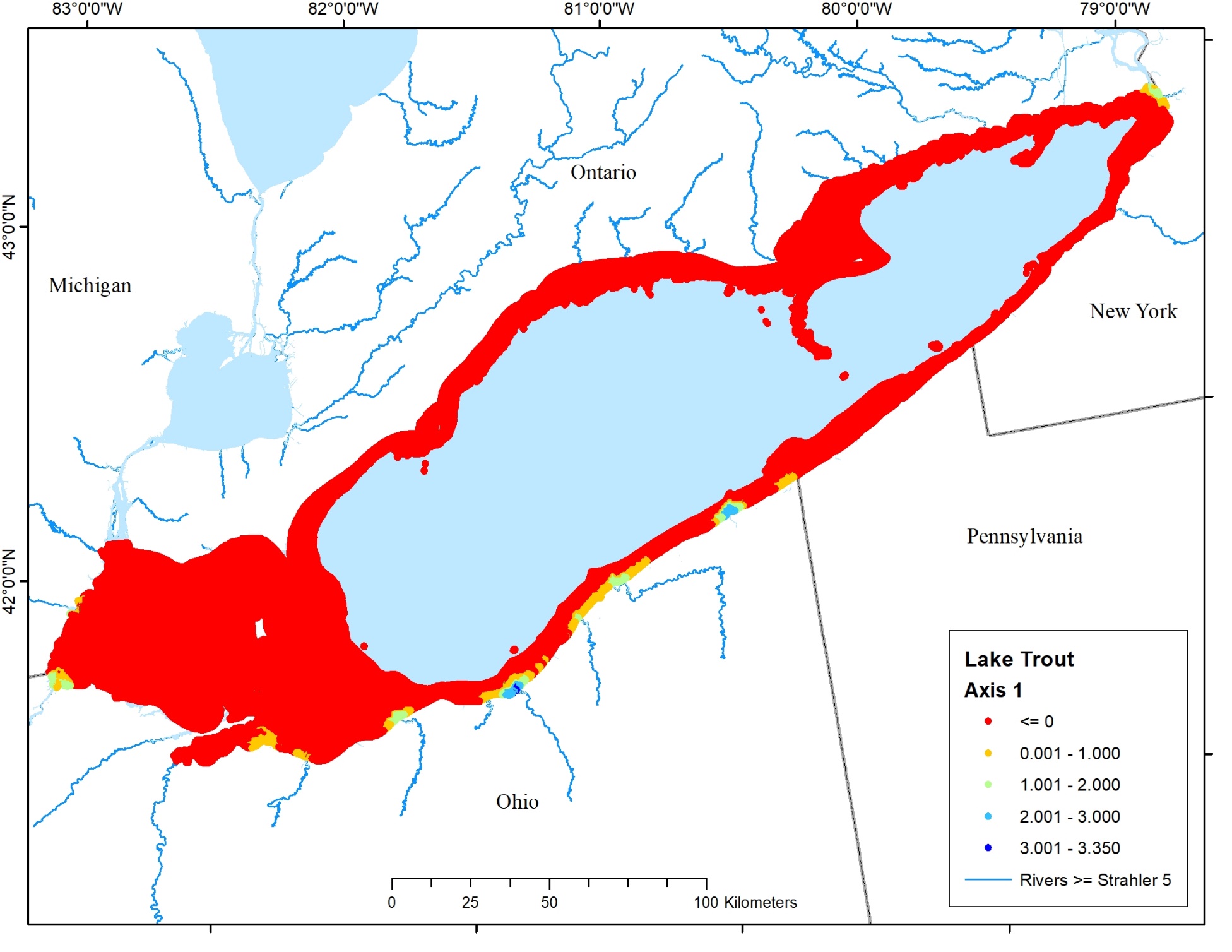
Fig. 8.c.


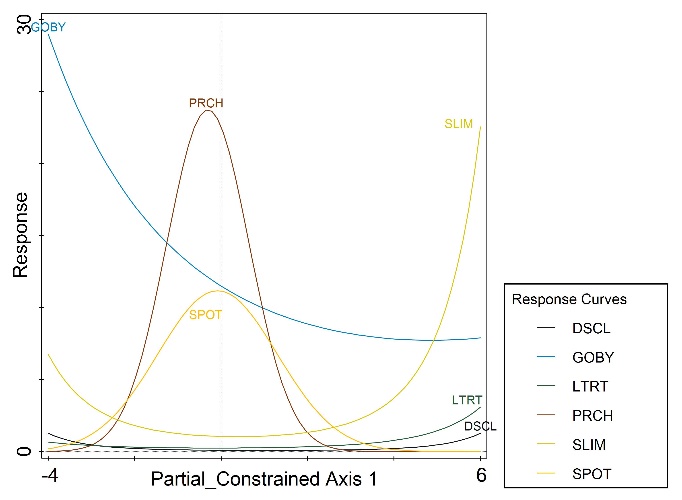

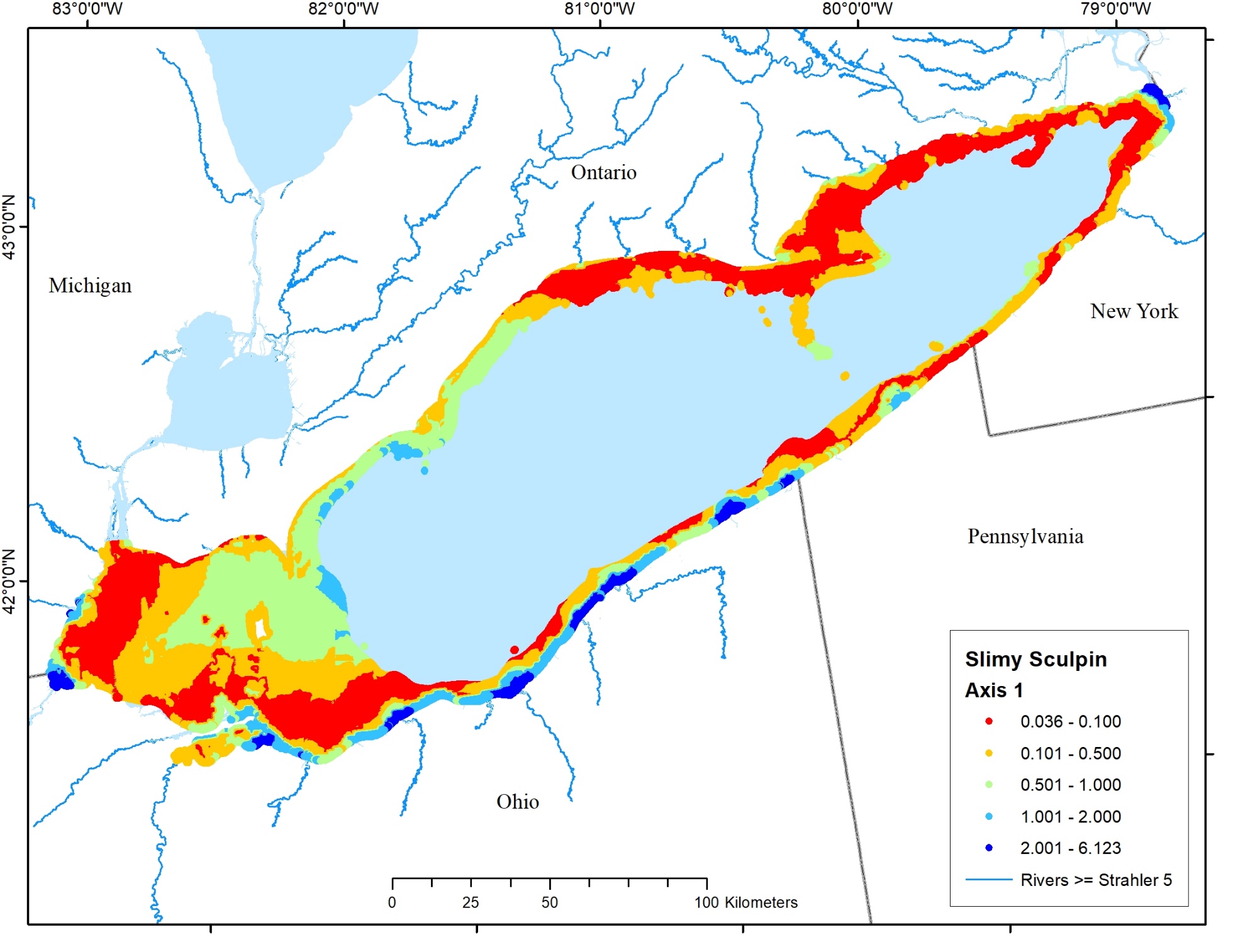
Fig. 8.d.


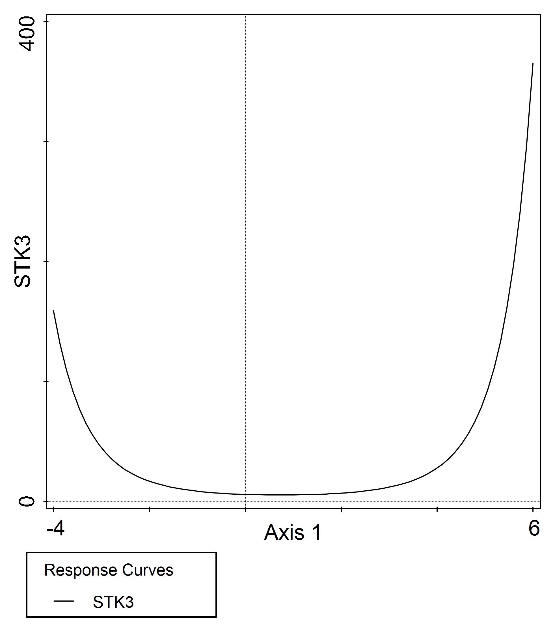

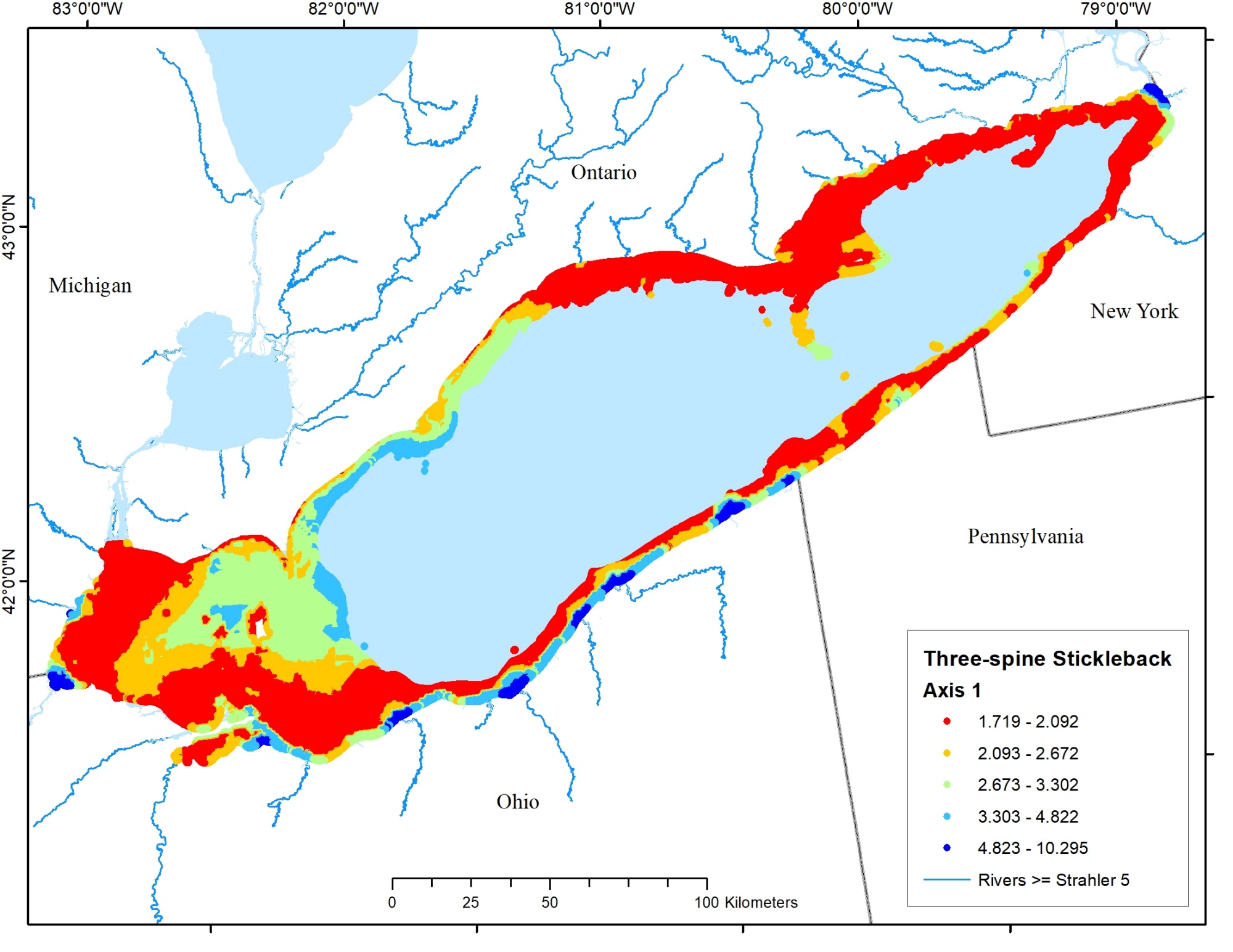
Fig. 8.e.

Fig. S9.a


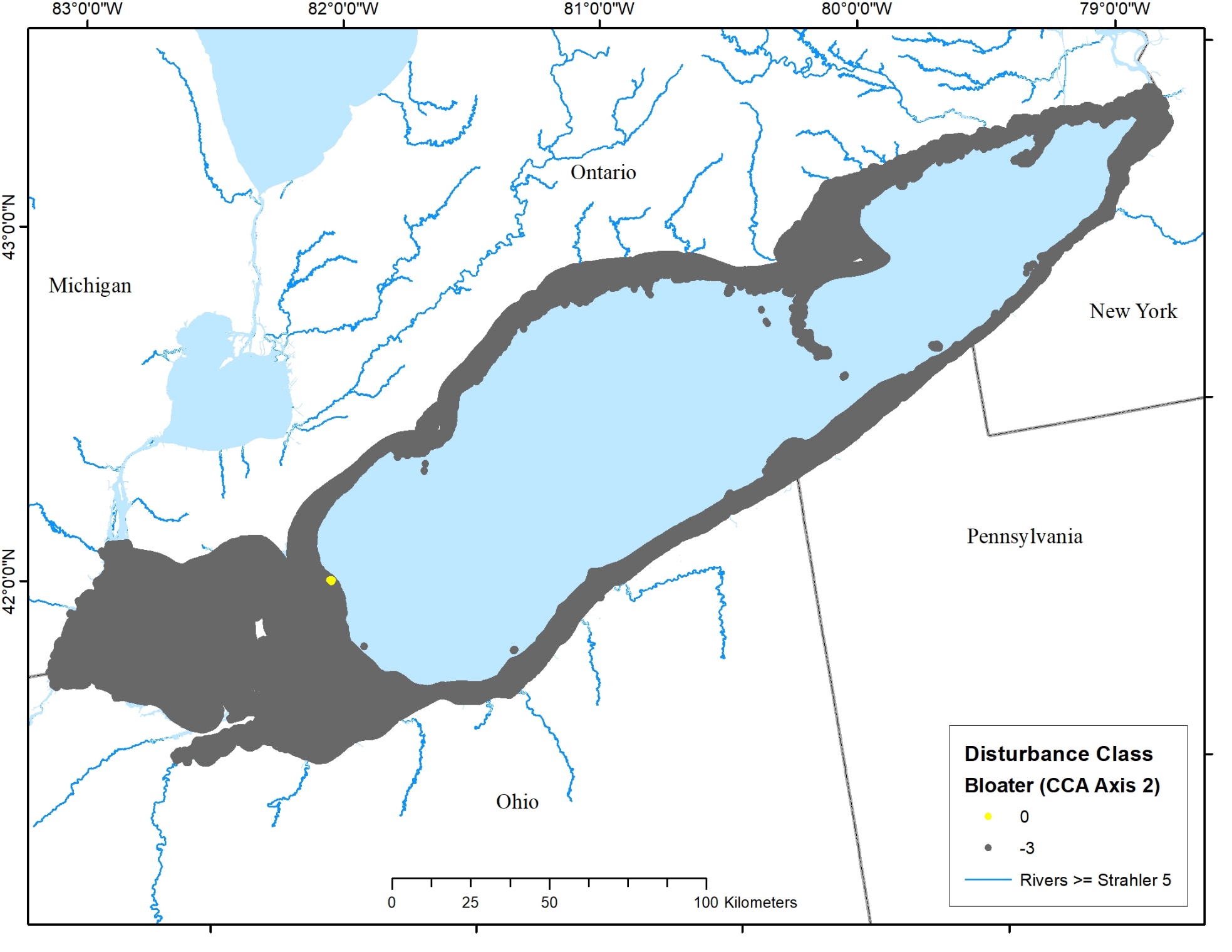

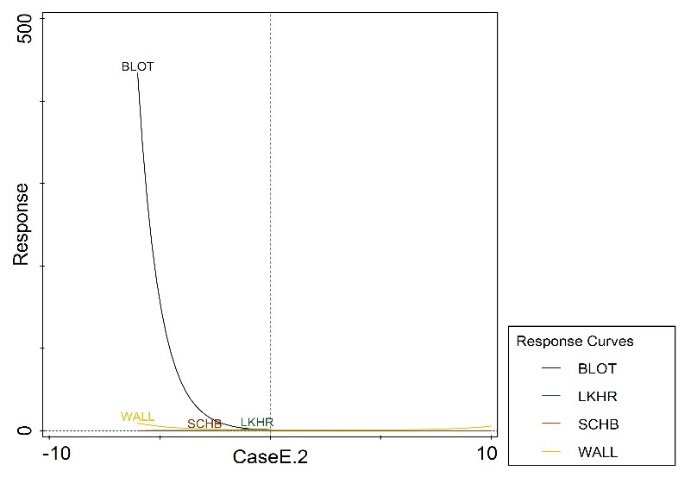


Fig. 9.b.


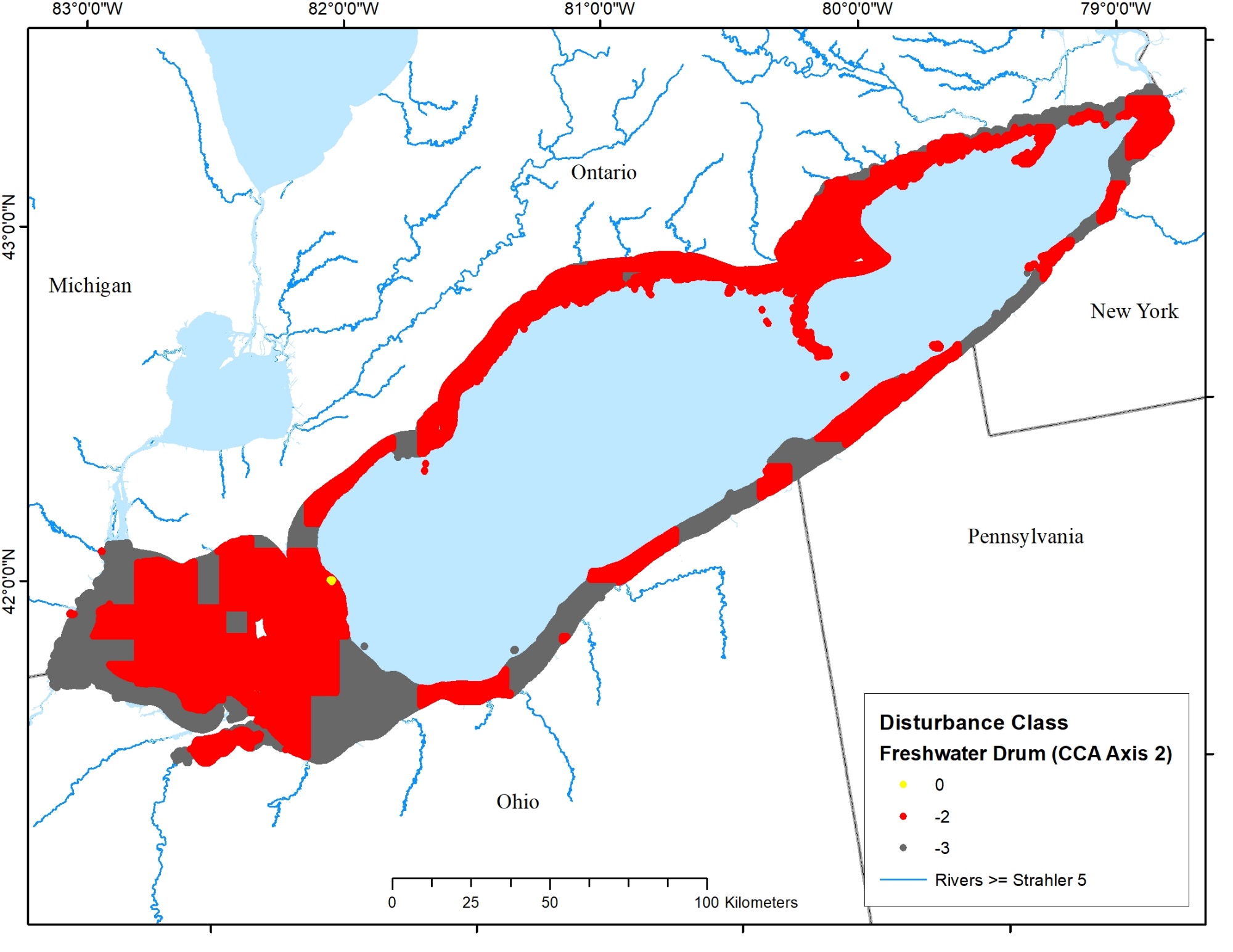


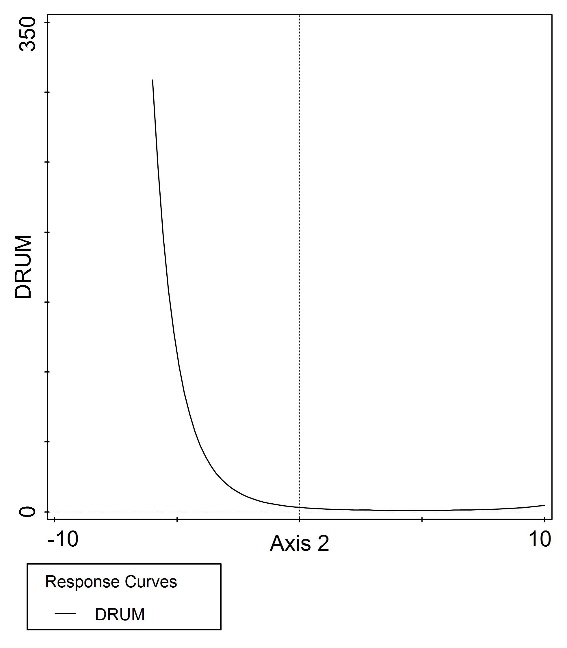

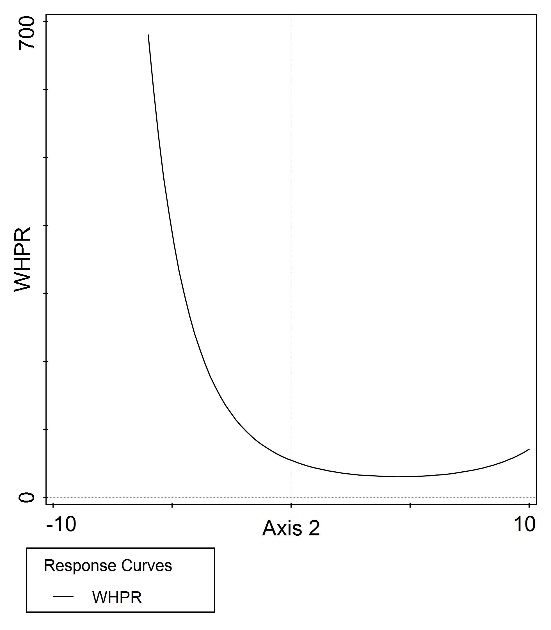

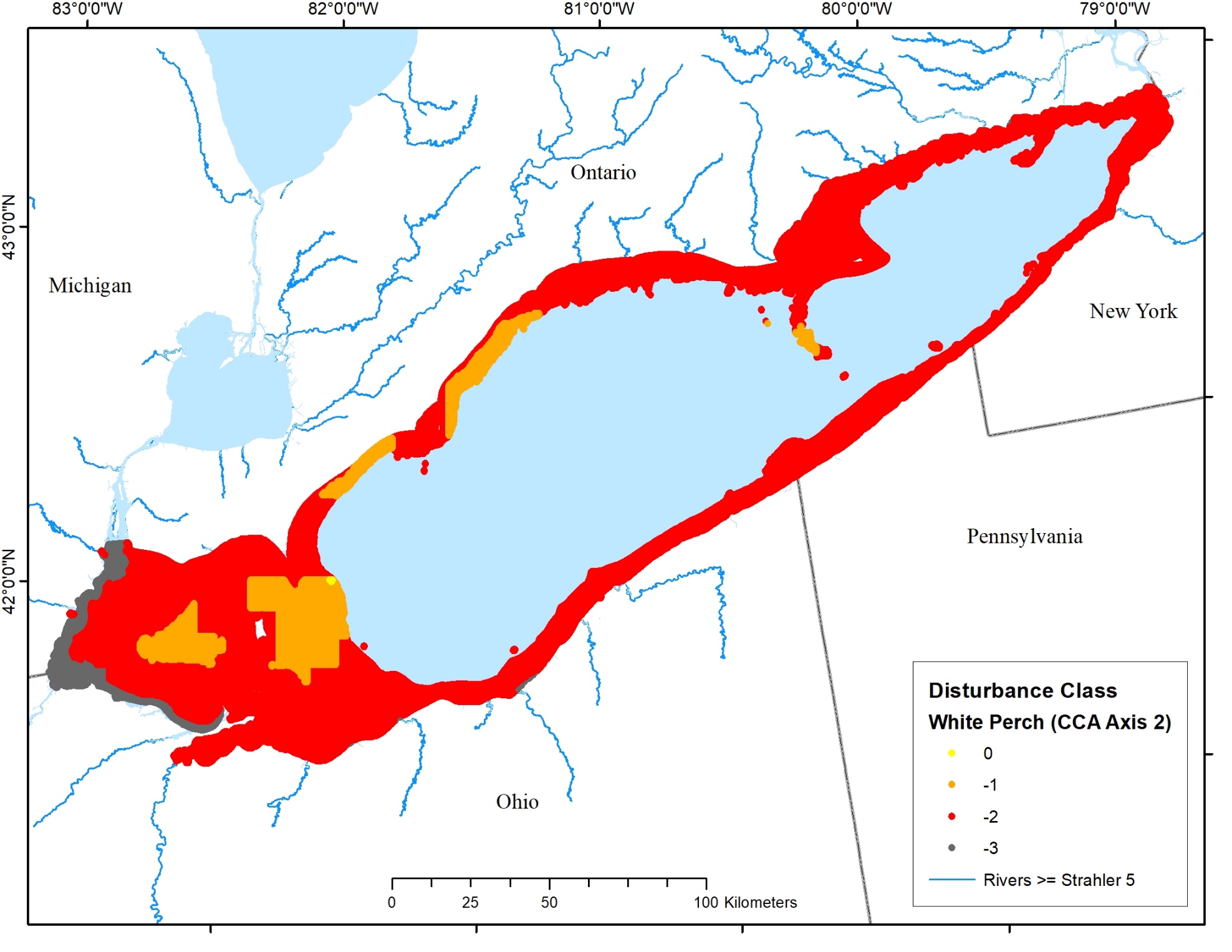
Fig. S9.c.


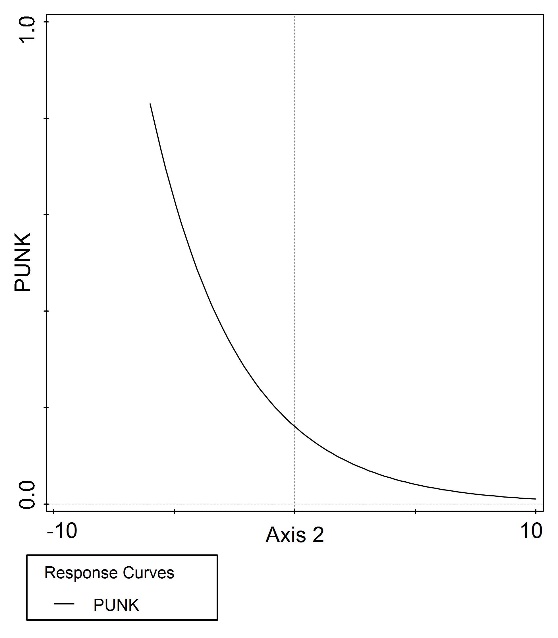

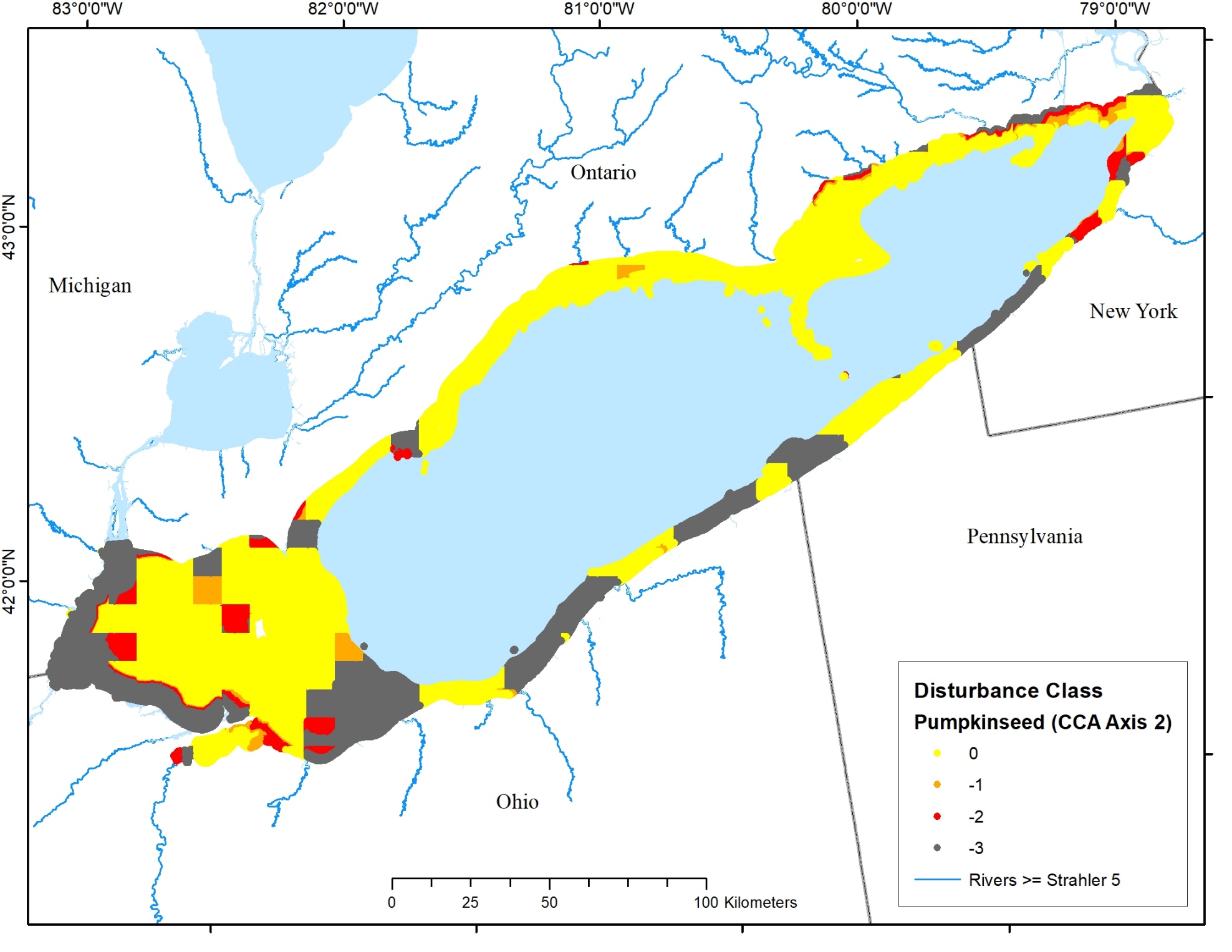
Fig. 9.d


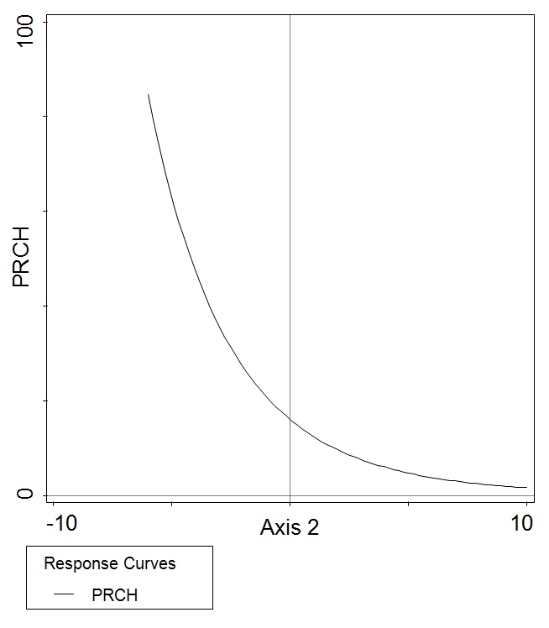

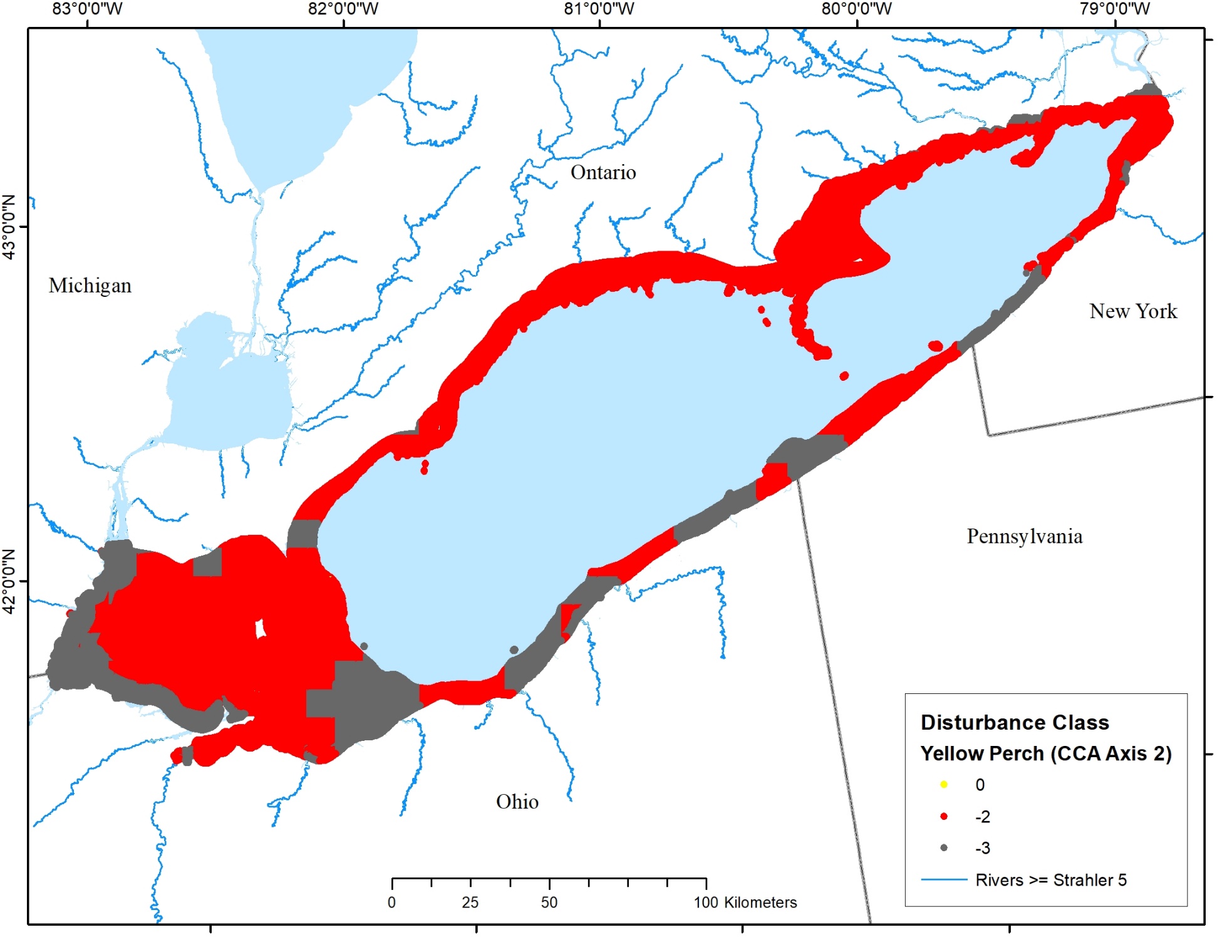
Fig. 9.e.


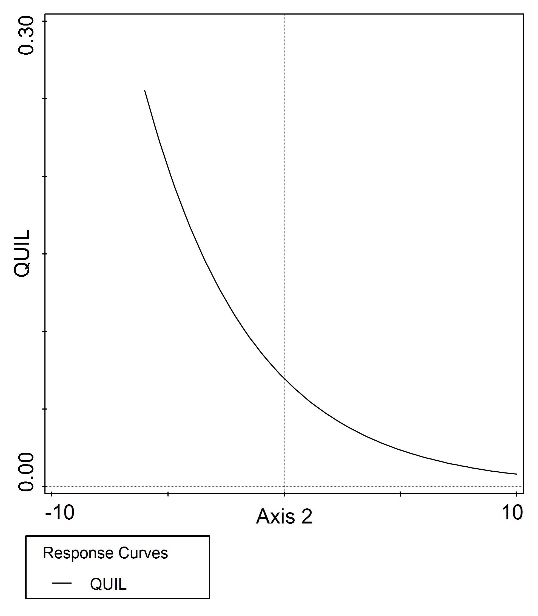

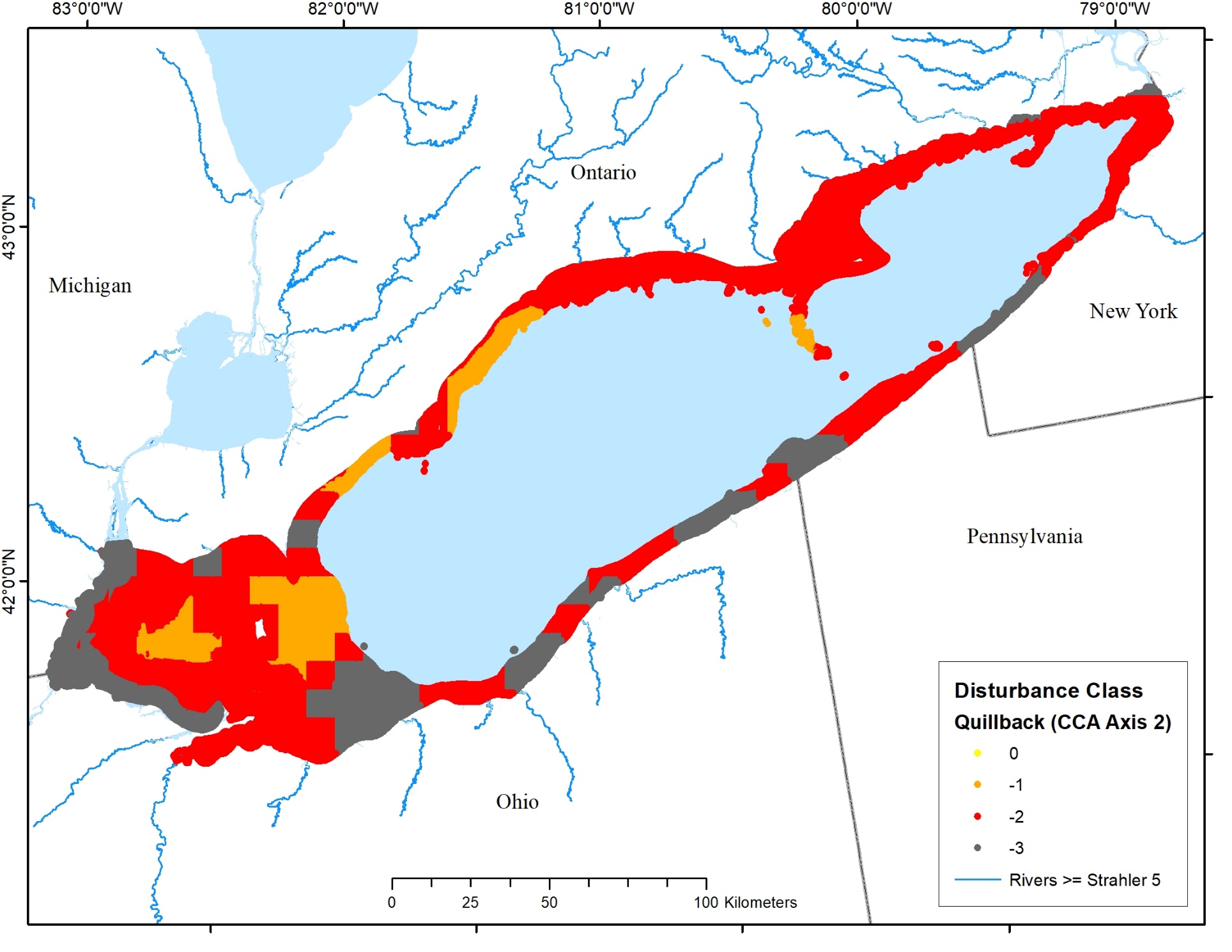
Fig. 9.f.


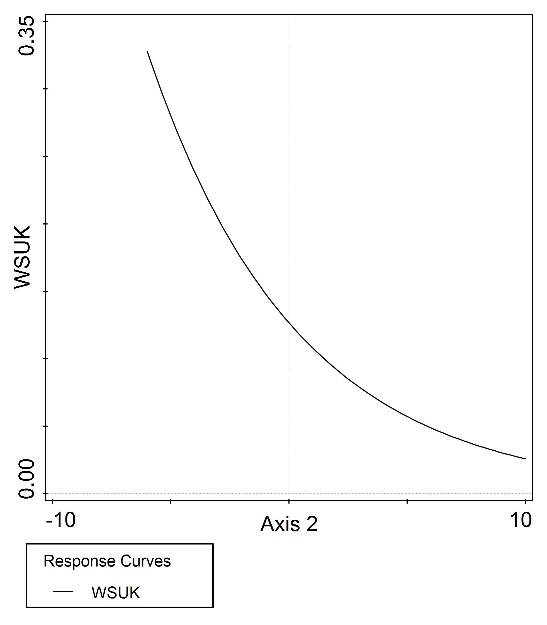

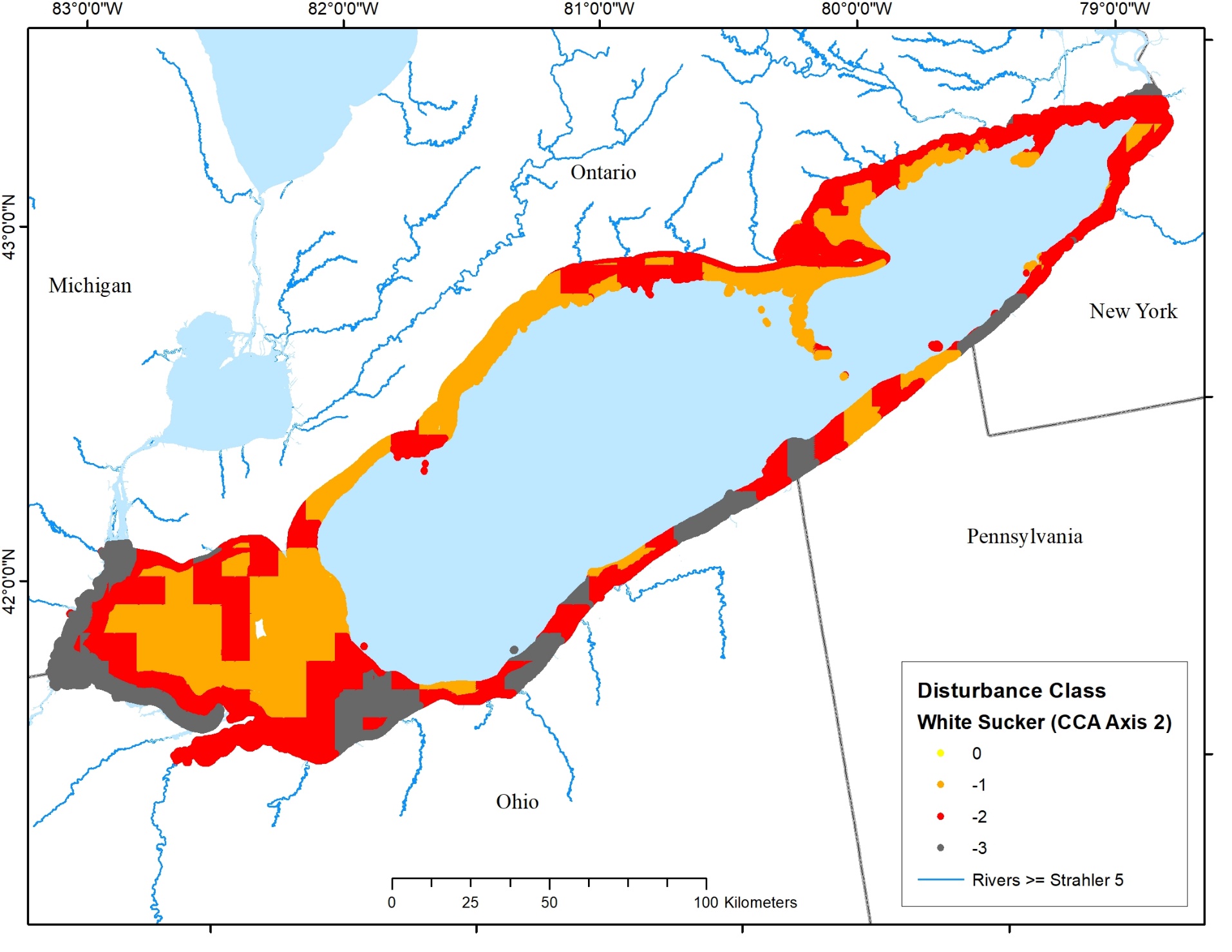
Fig. 9.g.


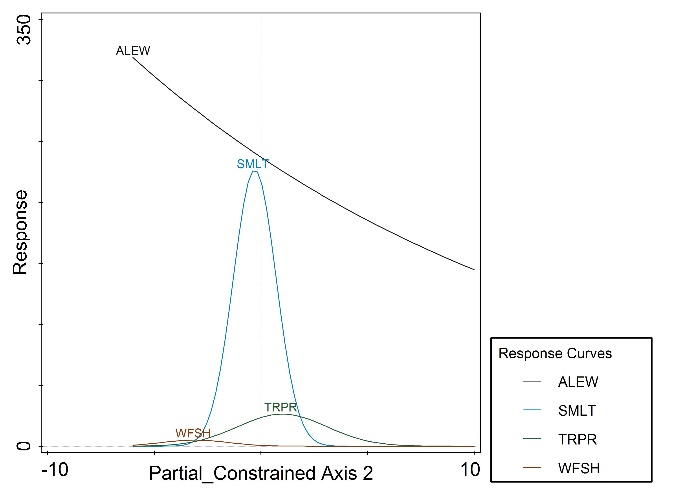

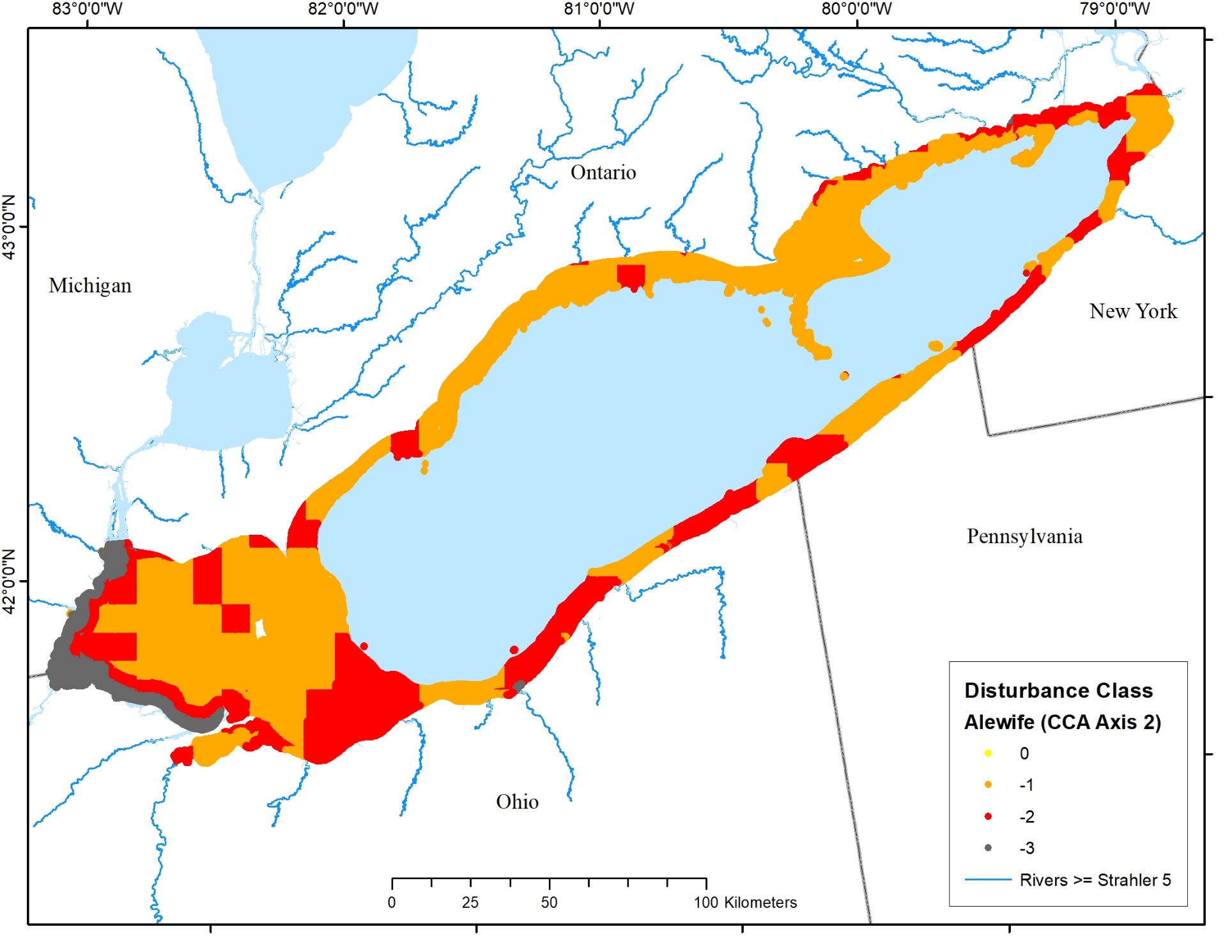
Fig. 9.h.


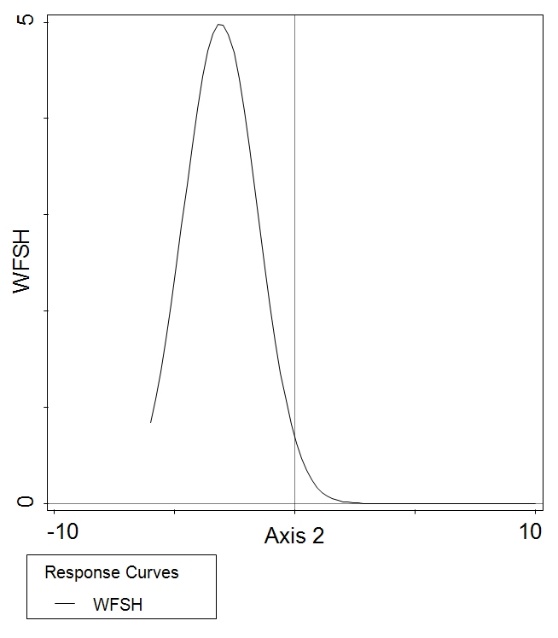

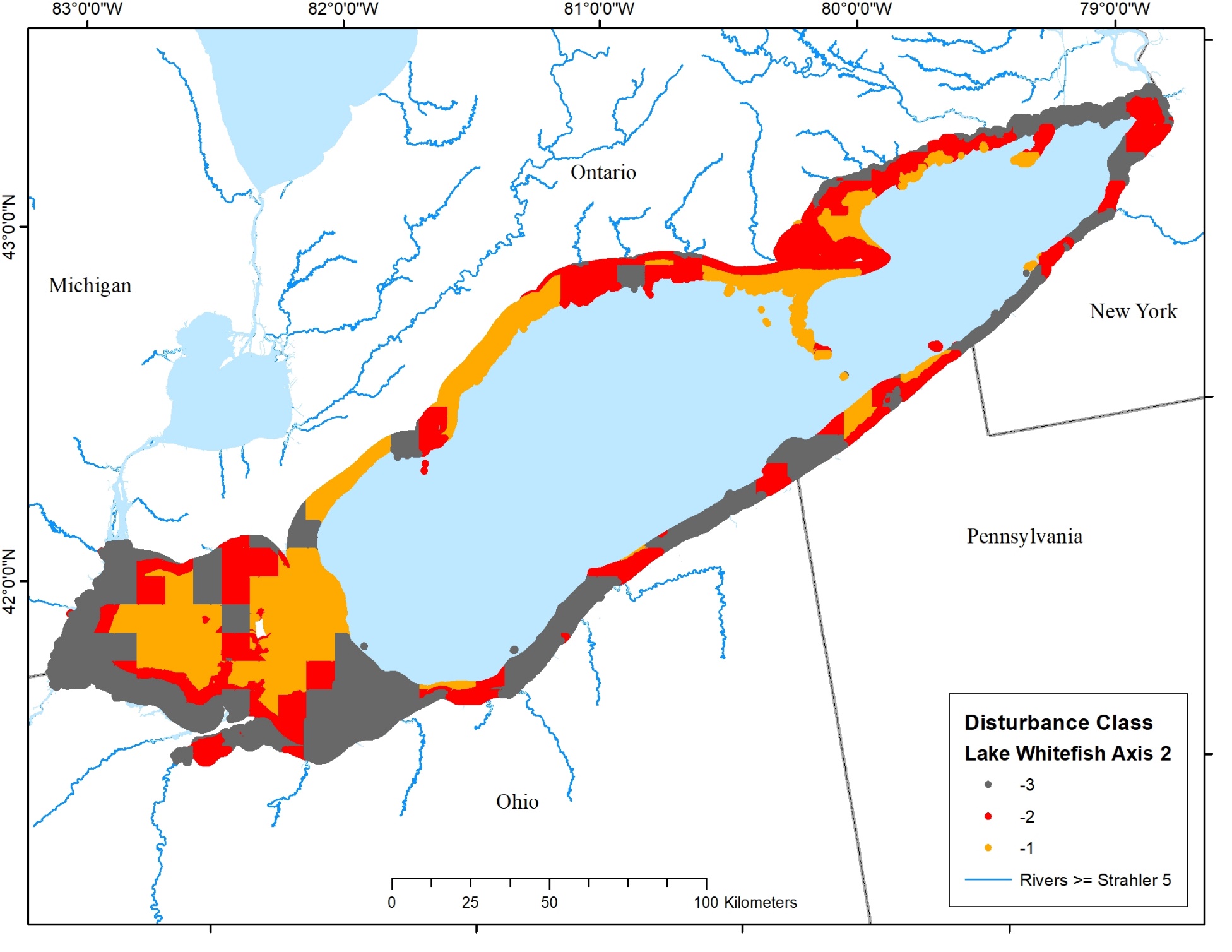
Fig. 10.a.


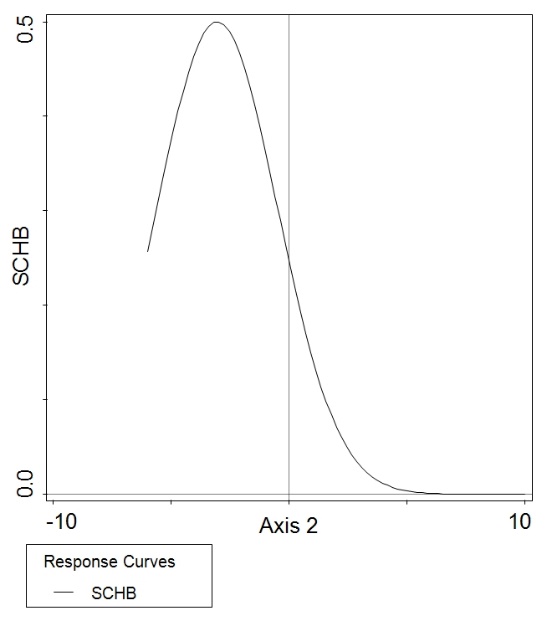

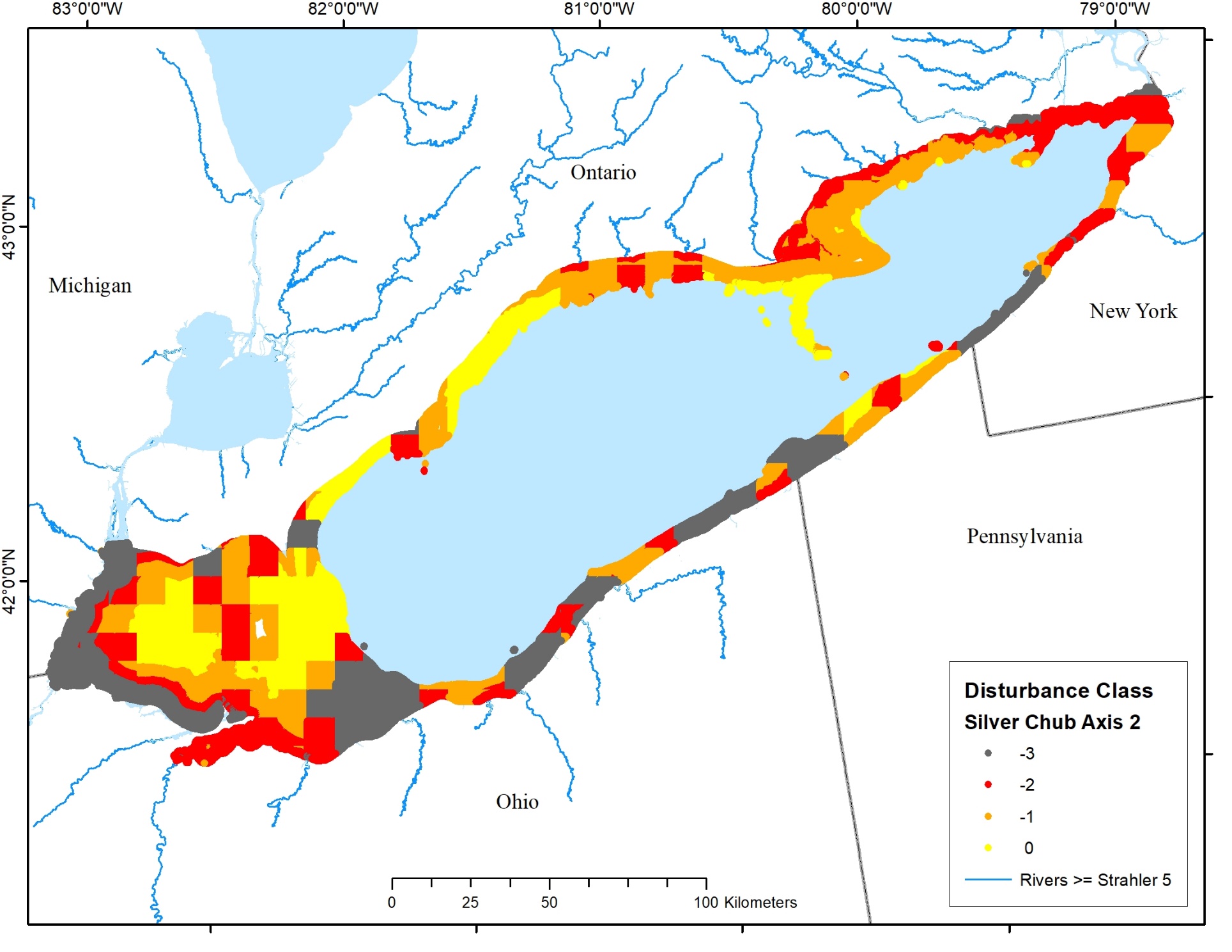
Fig. 10.b.


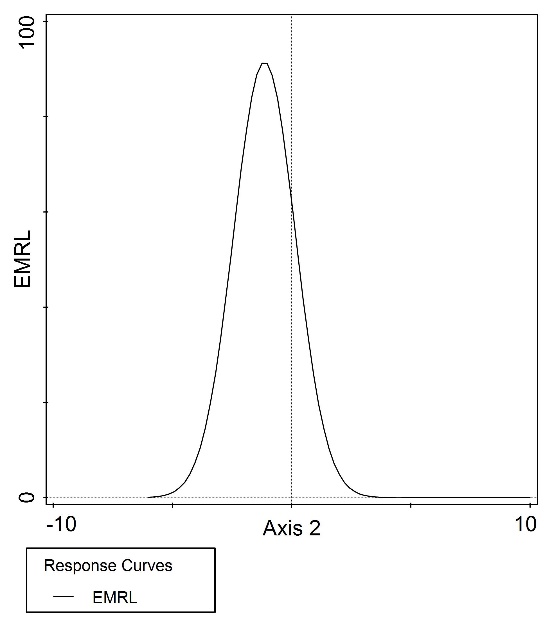

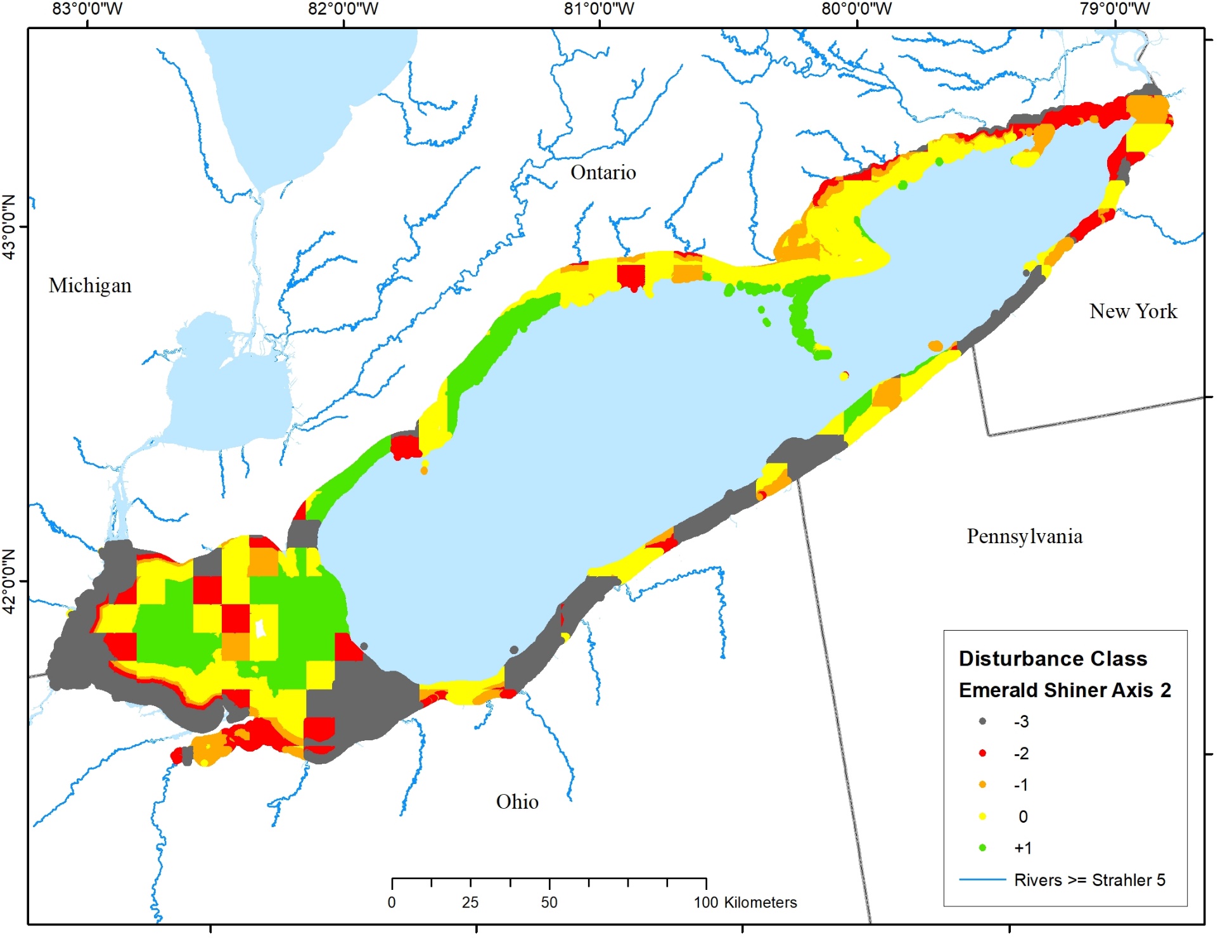
Fig. 10.c.


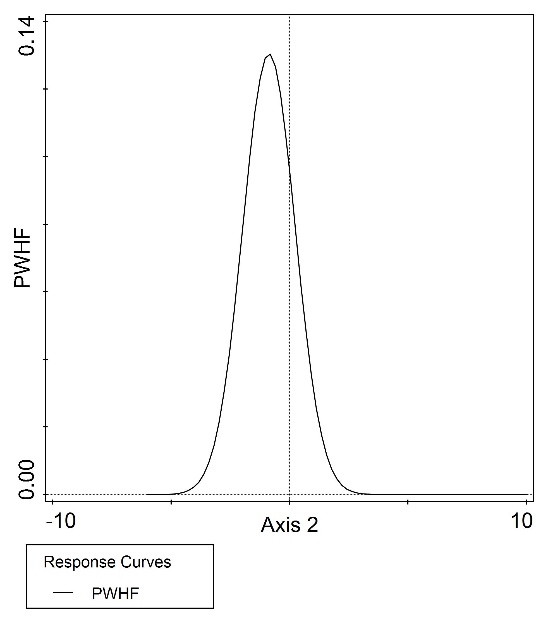

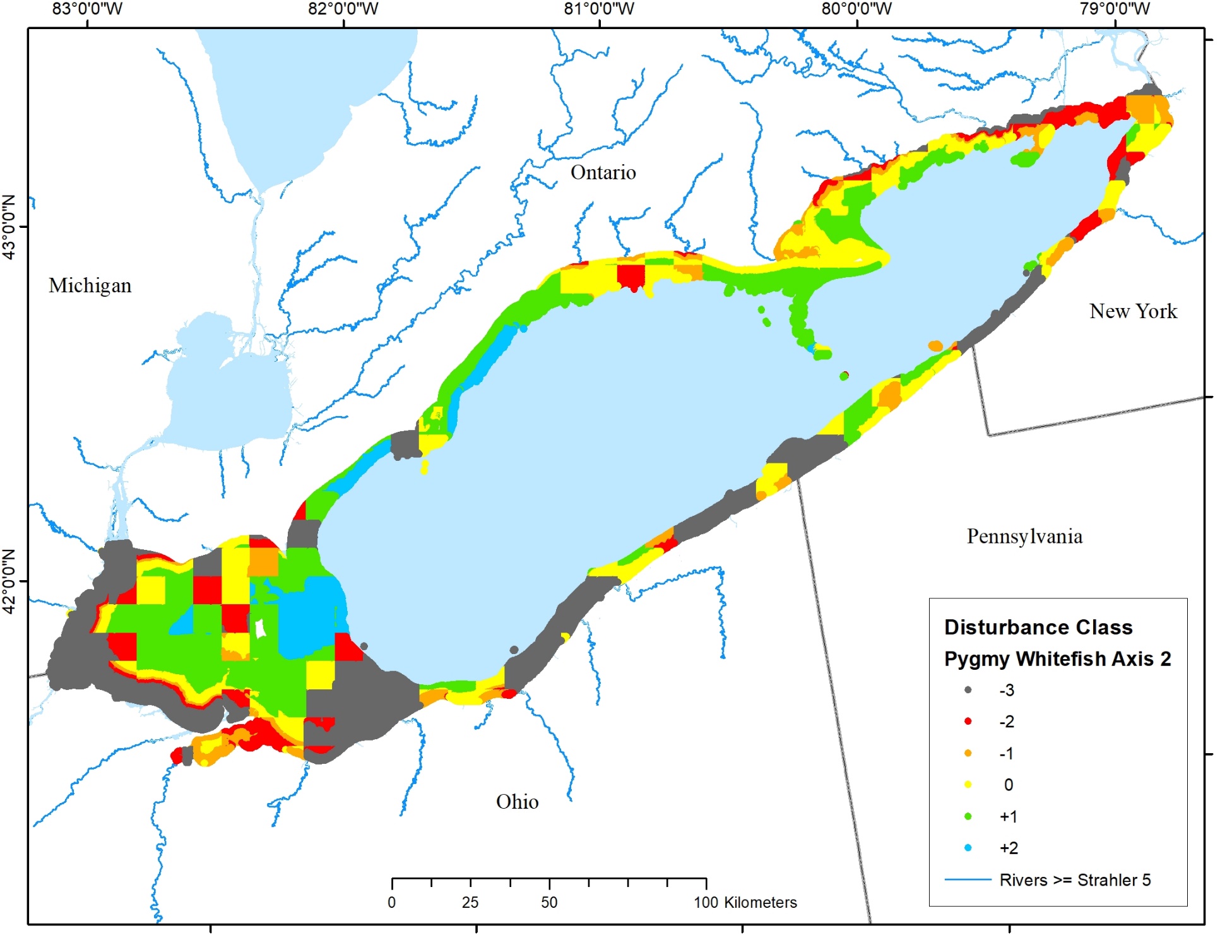
Fig. 10.d.


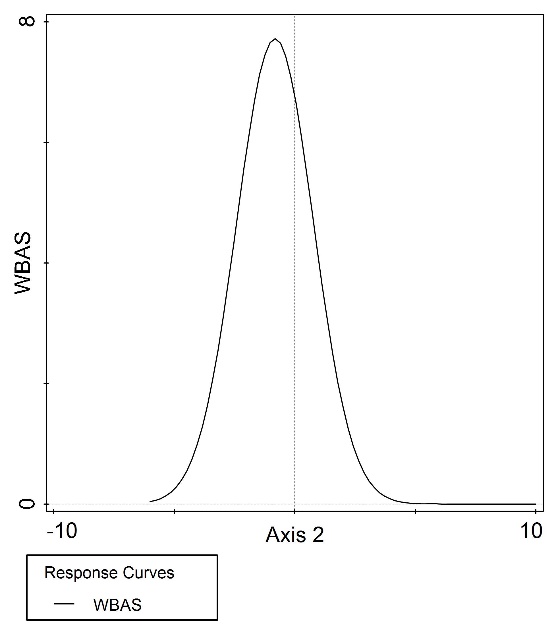

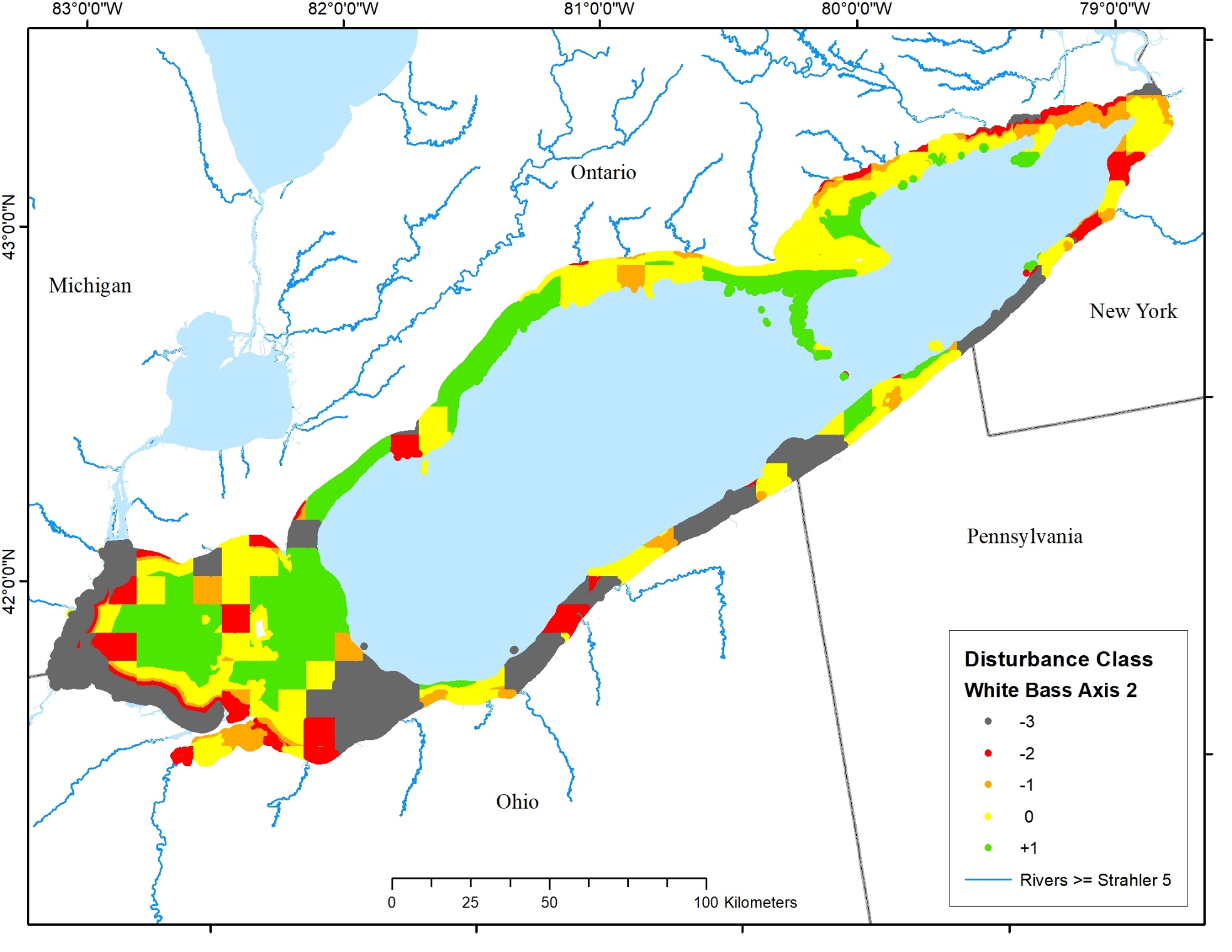
Fig. 10.e.

Fig. 10.f.


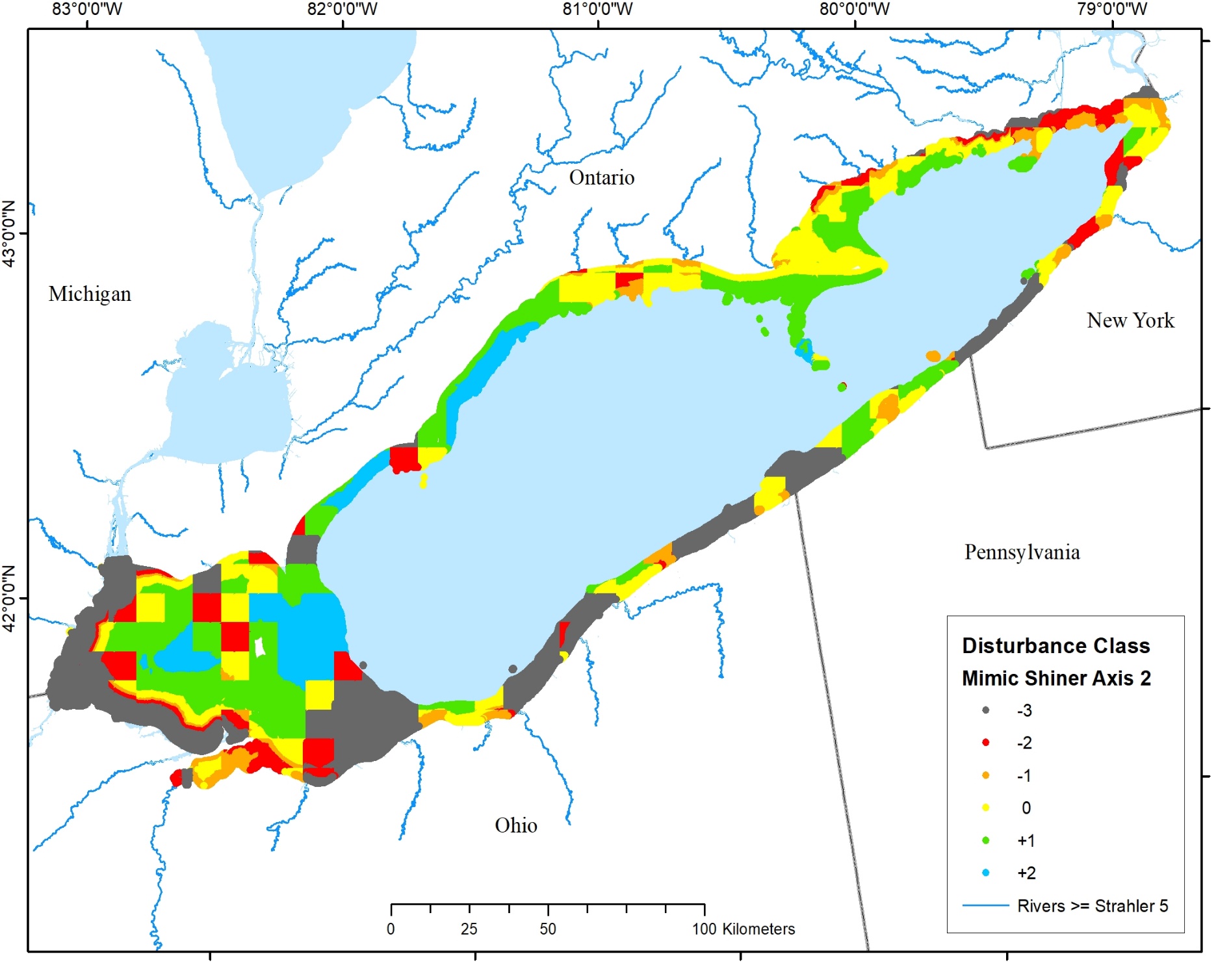

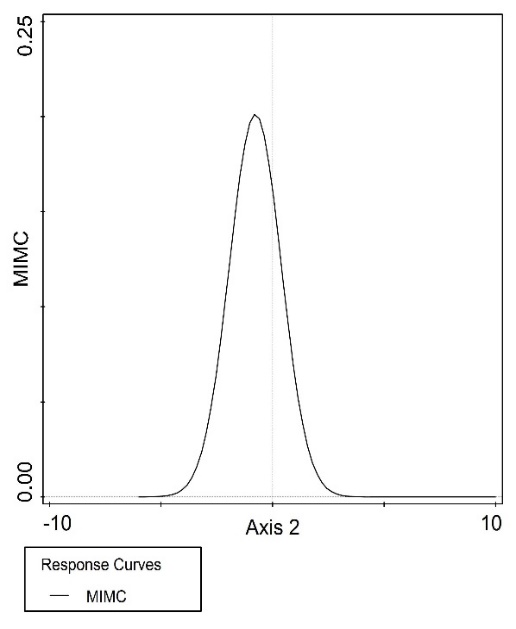


Fig. 10.g.


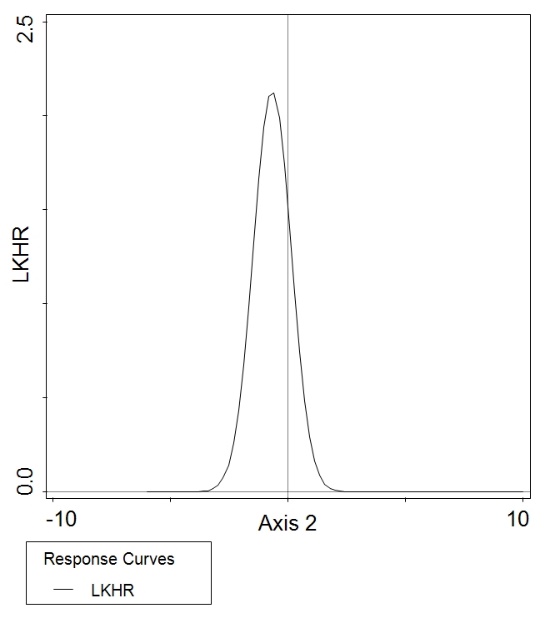

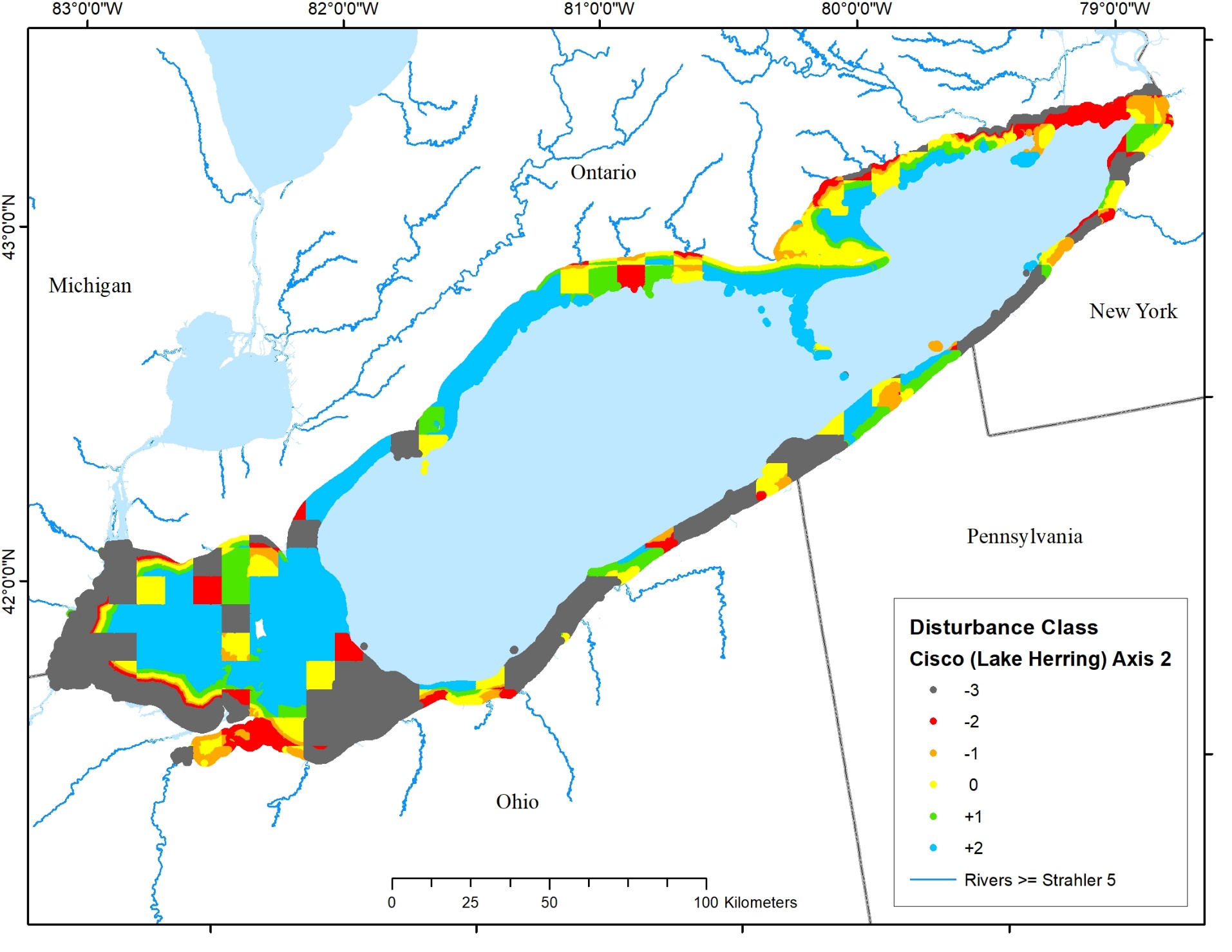
Fig. 11.a.

Fig. 11.b.

Fig. 11.c.

Fig. 11.d.

Fig. 11.e.

Fig. 11.f.

Fig. 11.g.

Fig. 11.h.

Fig. 12.a.

Fig. 12.b.

Fig. 12.c.

Fig. 12.d.

Fig. 13.a.

Fig. 13.b.

Fig. 14.a.

Fig. 14.b.

Fig. 14.c.

Fig. 14.d.

Fig. 15.a

Fig. 15.b.

Fig. 16 a.

Fig. 16.b.

Fig. 17.a.

Fig. 17.b.

Fig. 18.a.

Fig. 18.b.

Fig. 19.a.

Fig. 19.b.
